# High-resolution plasma metabolomics and thiamine status in critically Ill adult patients

**DOI:** 10.1007/s11306-024-02144-9

**Published:** 2024-07-27

**Authors:** Kursat Gundogan, Mary M. Nellis, Nurhayat T. Ozer, Serap S. Ergul, Gulsah G. Sahin, Sahin Temel, Recep C. Yuksel, Sami Teeny, Jessica A. Alvarez, Murat Sungur, Dean P. Jones, Thomas R. Ziegler

**Affiliations:** 1 Division of Intensive Care Medicine, Department of Internal Medicine, Erciyes University School of Medicine, Melikgazi, 38039 Kayseri, Turkey; 2 Division of Clinical Nutrition, Erciyes University Health Sciences Institute, Kayseri, Turkey; 3 Clinical Biomarkers Laboratory, Department of Medicine, Emory University, Atlanta, GA 30322 USA; 4 Division of Endocrinology, Metabolism, and Lipids, Department of Medicine, Emory University School of Medicine, Atlanta, GA USA; 5 Department of Medicine, Emory Center for Clinical and Molecular Nutrition, Emory University School of Medicine, Atlanta, GA USA; 6 Division of Pulmonary, Allergy, Critical Care and Sleep Medicine, Department of Medicine, Emory University School of Medicine, Atlanta, GA USA

**Keywords:** Critical illness, Energy metabolism, ICU patients, Metabolomics, Micronutrients, Thiamine, Vitamin

## Abstract

**Introduction:**

Thiamine (Vitamin B1) is an essential micronutrient and is classically considered a co-factor in energy metabolism. The association between thiamine status and whole-body metabolism in critical illness has not been studied.

**Objectives:**

To determine association between whole blood thiamine pyrophosphate (TPP) concentrations and plasma metabolites and connected metabolic pathways using high resolution metabolomics (HRM) in critically ill patients.

**Methods:**

Cross-sectional study performed at Erciyes University Hospital, Kayseri, Turkey and Emory University, Atlanta, GA, USA. Participants were critically ill adults with an expected length of intensive care unit stay longer than 48 h and receiving chronic furosemide therapy. A total of 76 participants were included. Mean age was 69 years (range 33–92 years); 65% were female. Blood for TPP and metabolomics was obtained on the day of ICU admission. Whole blood TPP was measured by HPLC and plasma HRM was performed using liquid chromatography/mass spectrometry. Data was analyzed using regression analysis of TPP levels against all plasma metabolomic features in metabolome-wide association studies (MWAS). MWAS using the highest and lowest TPP concentration tertiles was performed as a secondary analysis.

**Results:**

Specific metabolic pathways associated with whole blood TPP levels in regression and tertile analysis included pentose phosphate, fructose and mannose, branched chain amino acid, arginine and proline, linoleate, and butanoate pathways.

**Conclusions:**

Plasma HRM revealed that thiamine status, determined by whole blood TPP concentrations, was significantly associated with metabolites and metabolic pathways related to metabolism of energy, carbohydrates, amino acids, lipids, and the gut microbiome in adult critically ill patients.

**Supplementary Information:**

The online version contains supplementary material available at 10.1007/s11306-024-02144-9.

Thiamine, an essential water-soluble vitamin, was the first B vitamin to be discovered and thus was named vitamin B_1_ (Whitfield et al., 2018). Thiamine pyrophosphate (TPP) is the active form of thiamine in the human body (Frank, 2015). TPP is involved in a wide variety of biochemical pathways required to maintain normal tissue and organ function, including as a co-factor in glycolysis, the tricarboxylic acid (TCA) cycle and pentose phosphate pathway metabolism. For example, the pentose phosphate pathway has a fundamental role in glucose metabolism and is also a major route for the synthesis of many neurotransmitters, nucleic acids, lipids, amino acids, steroids, and glutathione (Polegato et al., 2019).

Critical illnesses are severe, life-threatening disease states with heterogeneous clinical presentations (Ferrario et al., 2016). Critically ill individuals exhibit variable levels of inflammation due to infection, organ dysfunction, and other factors that increase counter-regulatory hormones and cytokines which contribute to a hypercatabolic state associated with insulin resistance and muscle wasting (Cyr et al., 2021; Rousseau et al., 2021; Wendt et al., 2021). Disturbed metabolic pathways associated with such hypercatabolic conditions in critically ill patients may contribute to and/or result in mitochondrial/energetic dysregulation, cellular damage, and organ failure (Christopher, 2018; Flower et al., 2021; Gourd & Nikitas, 2020; McClave et al., 2019; Puthucheary et al., 2013; Siddiqui et al., 2020). Several reports, including our previous study, have shown that thiamine depletion may be common in intensive care unit (ICU) patients, especially those receiving chronic diuretic therapy prior to ICU admission (Attaluri et al., 2018; Gundogan et al., 2019). Since thiamine is involved in energy metabolism and the metabolic processing of other essential nutrients, low thiamine status may be associated with significant alterations in key metabolic pathways in ICU patients.

High-resolution metabolomics (HRM) analysis is a rapidly developing science that utilizes liquid chromatography and ultra-high-resolution mass spectrometry (LC–MS) to detect small molecules, including nutrient-related metabolites, in plasma, urine, tissue, and other biosamples (Jones, 2016; Jones et al., 2012). Current HRM methods use advanced data extraction and bioinformatics methods to detect tens of thousands of metabolic features in plasma derived from endogenous and exogenous sources, including dietary nutrients, intermediates of macro-and micronutrient metabolism, the gut microbiome, environmental exposures, commercial products, and drugs (Jones et al., 2012; Uppal et al., 2016). As examples, our recent studies have utilized plasma HRM to determine metabolites and linked metabolic pathways (e.g., amino acid metabolism, TCA cycle intermediates) associated with lean mass in working adults and in individuals with active pulmonary tuberculosis infection, respectively (Bellissimo et al., 2021; Collins et al., 2021). These and other studies have focused on a hybrid approach using targeted and untargeted (discovery)-based HRM detection of plasma metabolites and specific metabolic pathways enriched in significant metabolites to provide new insight into human metabolism and disease-associated pathophysiology (Alvarez et al., 2017). High-resolution metabolomics may be important for providing personalized and precision nutrition support for critical illness (Christopher, 2018; Siddiqui et al., 2020). Metabolomics data can be useful for assessing the risk related to nutritional deficiencies (Lasky-Su et al., 2017), clarifying the metabolic mechanism behind nutritional treatments (Amrein et al., 2021), comparing the use of enteral and parenteral nutrition (Gonzalez-Granda et al., 2021), and discovering new targets for nutritional interventions (Stolarski et al., 2022; Viana et al., 2021).

Nutritional metabolomics using HRM thus represents a novel tool to explore nutrition-related physiology and pathophysiology in catabolic states such as critical illness.

The aim of this pilot study was to determine metabolites and metabolic pathways linked to whole blood TPP concentrations in adult ICU patients on chronic diuretic therapy, who may be at particular risk for thiamine depletion.

## Materials and methods

The current study was conducted in the Erciyes University Medical ICU center in Kayseri, Turkey and in the Clinical Biomarkers Laboratory at Emory University, Atlanta, GA, USA.

The present clinical research was performed in accordance with the ethical standards of the responsible committees on human experimentation and with the Helsinki Declaration of 1975 and approved by the Erciyes University Ethics Committee (Date:15.01.2020, Number: 2020/35). Written informed consent was obtained from all patients or their legal representatives.

### Study participants

A total of 76 participants were enrolled who were treated with furosemide for at least 6 months before entering the ICU. The mean age was 69.3 ± 13.3 years (range 33–92 years). Females comprised 65% of the participants.

#### Inclusion criteria

(1) Participants were at least 18 years of age, (2) were deemed to require ≥ 48 h of ICU treatment, and (3) received furosemide therapy for 6 months or longer before ICU admission.

#### Exclusion criteria

(1) Participants were receiving high-dose oral thiamine (≥ 50 mg/day within 14 days prior to ICU admission).

## Demographic and clinical data

### At ICU admission

Demographic and anthropometric characteristics (age, gender, body mass index (BMI), reason for ICU admission, comorbidities and Acute Physiology and Chronic Health Evaluation II (APACHE II) score of the 76 study participants were recorded. The type, dose, and duration of diuretic use of all participants prior to admission to the Medical ICU were noted. Nutritional support information (i.e., type, volume, energy and macronutrients support), as well as insulin administration, were documented at ICU admission.

## Sample preparation and analysis

### Whole blood thiamine concentrations

Non-fasted whole blood samples were obtained on the day of ICU admission. TPP concentrations were determined by high-performance liquid chromatography (HPLC; Shimadzu, 8040, Immuchrom, Japan). The reference range for whole blood TPP is 28–78 μg/L (66.5–200 nmol/L) and thiamine deficiency was considered < 28 ng/mL (Evliyaoglu et al.,^2019^) The blood samples were stored at − 80 °C until completion of the study for batch analysis in the laboratory.

### Plasma for metabolomics analysis

Blood samples obtained on the day of ICU admission were centrifuged at 1500×*g* at 4 °C 10 min. EDTA plasma samples were separated and stored at − 80 °C until all participant samples were collected. Samples were shipped on dry ice to Atlanta, GA for metabolomics batch analysis in the Clinical Biomarkers Laboratory at Emory University, Atlanta.

### High-resolution metabolomics

We compared patients with lower versus higher TPP concentrations using both targeted plasma HRM (pathways known to be dependent upon thiamine as a co-factor, such as the pentose phosphate pathway and the TCA cycle) and untargeted plasma HRM analytical approaches.

Plasma was analyzed by high-resolution mass spectrometry as previously described (Liu et al., 2016; Soltow et al., 2013). Plasma samples (50 µL) were mixed with 100 µL acetonitrile containing 1.25µL internal standard solution with eight stable isotopic chemicals representing multiple small-molecule classes ([13C6]-d-glucose, [15N,13C5]-l-methionine, [13C5]-l-glutamic acid, [15N]-l-tyrosine, [3,3-13C2]-cystine, [trimethyl-13C3]-caffeine, [U-13C5, U-15N2]-l-glutamine, [15N]-indole) (Soltow et al., 2013). Samples were then equilibrated on ice for 30 min and centrifuged at 16000×*g* for 10 min to remove precipitated proteins. The supernatant was added to an autosampler vial and maintained at 4 °C until analysis. Liquid chromatography was performed on triplicates of each sample using both C18 (Higgins C18 stainless steel column, 2.1 ×  50 mm) and hydrophilic interaction liquid chromatography (HILIC, Waters XBridge BEH Amide XP HILIC column, 2.1 × 50 mm) liquid chromatography columns followed by negative (C18) or positive (HILIC) electrospray ionization (ESI) and high-resolution mass spectrometry (Thermo Orbitrap Fusion Tribrid).

Mass spectral data were collected over a 5-min run period at a resolution of 120,000 and mass-to-charge (*m/z*) scan range of 85–1275. Each batch of 40 samples also included six triplicates of a pooled normal human plasma reference standard (Qstd3) (Go et al., 2015). The raw data files were extracted and aligned using apLCMS (Yu et al., 2009) and xMSanalyzer (Uppal et al., 2013). The resulting feature table consisted of unique metabolic features (metabolites) defined by accurate mass *m/z*, retention time, and ion intensity. Data was filtered to exclude *m/z* features having a median coefficient of variation within technical replicates ≥ 75%, and only samples with Pearson correlation within technical replicates ≥ 0.7 were used for downstream analysis (Go et al., 2015). Triplicates were median summarized with the condition that at least two replicates had non-missing values.

Metabolite annotation was performed using xMSannotator, an R program that uses a clustering algorithm to provided tentative annotations of features using publicly available databases, including the Kyoto Encyclopedia of Genes and Genomes (KEGG) and the Human Metabolome Database (HMDB) (Uppal et al., 2017).

### Statistical analysis

Prior to the analysis, the feature table was filtered to retain the features having non-zero values in at least 50% of the samples overall and in at least 80% of the samples in a group. After filtering, the feature intensities were log2 transformed and quantile normalized. Multiple linear regression analysis was used to conduct metabolome-wide association analysis (MWAS) to determine the associations of metabolic features (metabolites) with TPP concentrations, adjusting for age, sex, BMI, and APACHE II score as a priori covariates.

Given the relatively low number of participants with TPP levels below the lower limit of normal, we also performed a separate secondary regression analysis to compare participants with TPP concentrations in the lowest versus the highest tertile of whole blood TPP concentrations. This tertile analysis was also adjusted for age, sex, BMI, and APACHE II score. We adjusted for APACHE II score in HRM analysis given that inflammation during critical illness may alter blood concentrations of several micronutrients, with redistribution from blood to tissues (Berger et al., 2022).

Pathway enrichment analysis was performed using mummichog (v2.0.6), which uses permutation analysis to map features based on both *m/z* and retention time to specific pre-identified human metabolic pathways (Li et al., 2013). For the MWAS, all features associated with thiamine with a raw P-value < 0.05 were included in the pathway analysis. For the tertile analysis, all features that discriminated between tertiles with a raw p-value < 0.05 were included. Using a raw P-value < 0.05 (compared to the much more stringent false discovery rate correction) protects against type II error and prevents information loss when performing pathway analysis. The mummichog algorithm maps the significant features to pathways and determines the P-values for these enriched pathways after adjusting for the null distribution of pathway p-values established using 500 permutations, which protects against type I error. Thus, running mummichog using metabolomic features with P< 0.05 effectively provides a compromise to handle both type I and type II error (Uppal et al., 2016). As additional protection against type I error, only the pathways enriched with at least four overlapping metabolites and one Level 1 confirmed metabolite (see below) were considered as significantly relevant to thiamine status.

Metabolites within specific pathways were identified and confirmed by comparison to an in-house library of metabolites previously validated using ion-dissociation tandem mass spectrometry (MS/MS) and co-elution with authentic standards(Liu et al., 2020). Identification scores were assigned for metabolites based on an adaptation of the criteria proposed by Schymanski et al. (Schymanski et al., 2014): Level 1: confirmed by matching to an in-house library of MS/MS validated metabolites established using authentic standards Level 2: confirmed by matching to validated adducts from our in-house library and matches with online databases; Level 3: matches online databases and correlated with metabolites in pathways via mummichog; Level 4: computationally assigned annotation using the biostatistical program xMSannotator (medium or high confidence); Level 5: accurate mass (*m/z*) match only.

## Results

The median age of study patients was 69 (62.5–79.5) years and 65% were female. The median APACHE II score on ICU admission was 14 (11–20). The most common reasons for ICU admission were acute respiratory failure (62.3%) and metabolic disorders, including renal and hepatic failure, severe hyperglycemia, and hepatic encephalopathy (63.6%). Some participants had more than one of these conditions upon ICU admission. Chronic liver cirrhosis (53.2%) and congestive heart failure (50.6%) were the most frequent co-morbid diseases of the study participants and were the primary reason for chronic diuretic use prior to ICU admission. Their median duration of ICU stay was 5 days (3–8) and ICU mortality rate was 23.4%. The median TPP concentration was 41.4 (32.2–57.5) with a mean value of 48.5 ± 24.8 (SD) ng/mL. The whole blood TPP normal reference value: 28–85 ng/mL. The proportion of patients with below normal TPP levels was 14.3%. Additional demographic and clinical characteristics of the participants are listed in Table 1.

**Table 1:** Study subject demographic and clinical characteristics

Variable	Total (n = 76)
Age (years, median (IQR))	69.0 (62.5.0–79.5)
Mean ± SD	69.3 ± 13.3
Gender, n (%)	
Male	26 (35.0)
Female	50 (65.0)
Body mass index (kg/m^2^, median (IQR)) mean ± SD	27.5 (24.4–33.1) 29.0 ± 6.2
Admit to ICU, n (%)	
From emergency department	41 (53.2)
From general floor	31 (40.3)
From outside hospital	5 (6.5)
Reason for admission in ICU*, n (%)	
Acute respiratory failure	48 (62.3)
Metabolic disorder (renal failure, hyperglycemia and/or and hepatic failure)	49 (63.6)
Cardiovascular disorders	11 (14.2)
Neurological disorders	8 (10.4)
GI bleeding	8 (10.4)
Trauma	8 (10.4)
Sepsis/Septic shock	7 (9.1)
Comorbidities*, n (%)	
Liver cirrhosis	41 (53.2)
Heart failure	39 (50.6)
Renal failure	28 (36.4)
COPD	26 (33/8)
APACHE II score (median (IQR)) mean ± SD	14.0 (11.0–20.0) 15.6 ± 6.1
TPP whole blood concentrations, mean ± SD	48.6 ± 24.9
TPP whole blood concentrations with APACHE II score < 14, mean ± SD	46.3 ± 19.5
TPP whole blood concentrations with APACHE II score ≥ 14, mean ± SD	50.6 ± 28.9
SOFA score (median (IQR)), mean ± SD	4.0 (3.0–6.0) 4.7 ± 2.7
Glasgow coma score (median (IQR)) mean ± SD	15.0 (12.0–15.0) 12.8 ± 3.7
Treatment with mechanical ventilation, n (%)	12(15.6)
Treatment with vasopressors, n (%)	18 (23.4)
Total calorie intake on ICU admission day (median (IQR) (n = 52)	625 (400–1000)
TPP levels (ng/mL, median (IQR) and mean ± SD	41.4 (32.2–57.5) 48.5 ± 24.8

*APACHE II* The acute physiology and chronic health evaluation, *COPD* chronic obstructive pulmonary disease, *CRP* C-reactive protein, *ICU* intensive care unit, *SOFA* sequential organ failure assessment, *TPP* thiamine pyrophosphate

*IQR* Interquartile range (25th quartile–75th quartile)

^*^Some participants had multiple comorbidities and primary reasons for ICU admission

Whole blood TPP normal reference value: 28 –78 ng/mL

## HRM analysis

A total of 18,189 plasma metabolomic features were detected in the C18/ESI negative mode of analysis, with 16,598 features used in downstream analysis after pre-processing. There were 21,737 total plasma metabolomic features detected in the HILIC/ESI positive mode, with 19,576 features used in downstream analysis after pre-processing.

**Fig. 1:**
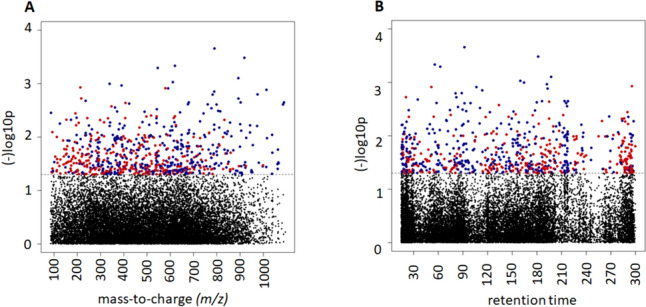
Manhattan plots of significant metabolites related to TPP levels in the 76 critically ill participants on admission to the ICU using a metabolome wide association study (MWAS) of metabolites from the C18/ESI negative column (A and B). **A** Type 1 Manhattan plot where each dot corresponds to one metabolic feature plotted according to mass-to-charge (*m/z*) ratio on the x-axis and–log10 P-value on the y-axis. **B** Type 2 Manhattan plot where each dot corresponds to one metabolic feature plotted according to chromatographic MS retention time in seconds. Blue dots represent negative associations with whole blood TPP concentrations and red dots indicate a positive association with whole blood TPP levels, adjusted for age, APACHE II score, sex, and BMI. Black dots (below the P < 0.05 dotted line) represent metabolites not significantly related to TPP levels in the participants. Of the total of 672 significant metabolites, 368 were negatively associated and 304 were positively associated with whole blood TPP levels at raw P < 0.05. When comparing the highest TPP tertile to the lowest tertile (not shown), there were 718 features that varied between tertiles at raw P < 0.05

Type 1 (*m/z*) and Type 2 (retention time) Manhattan plots were used to visualize the metabolomic features associated with TPP status for both MWAS and tertile analysis, respectively **(**Fig. 1 , tertile analysis not shown). In the C18/ESI-, data, there were 672 features (368 negatively, and 304 metabolites positively) associated with TPP levels at raw P < 0.05 based on MWAS analysis, and in the HILIC/ESI + data, 1033 features were associated with TPP levels (505 negatively associated, and 528 positively associated) (Suplemental Tables 1 and 2, respectively). When comparing the highest TPP tertile to the lowest tertile, there were 718 features in the C18/ESI-data and 1063 features in the HILIC/ESI + data that varied between tertiles at raw P < 0.05 (Supplemental Tables 3 and 4, respectively).

**Table 2:** C18/ESI- and HILIC/ESI + MWAS analysis in association with TPP concentrations

Metabolic Pathway	*m/z*	Time (s)	Adduct	Putative annotation	Metabolite P-value	Association with TPP levels	ID score
Pentose phosphate pathway	195.0511	19	M^-^H [−]	Gluconate	0.043	Negative	1
	115.0399	269	M^-^H2O^-^H [−]	Deoxyribose	0.030	Negative	3
Linoleate metabolism	295.2267	23	M^ + ^H [+]	OxoODE	0.028	Negative	3
	261.222	214	M^-^H20-H [−]	Linoleate	0.034	Negative	2
Bile acid biosynthesis	401.3423	28	M ^+ ^H [+]	α-Hydroxy-cholestenone	0.028	Positive	3
Pyrimidine metabolism	263.0455	19	M^ +^ Cl [−]	Deoxyuridine	0.005	Negative	2

*ESI* electrospray ionization, *HILIC* hydrophilic interaction liquid chromatography, *ID* identification score, *MWAS* metabolome-wide association study, *Octenoyl-CoA* Octenoyl-Coenzyme A, *OxoODE* Oxooctadecadienoic acid

Raw P-values < 0.05

**Fig. 2A:**
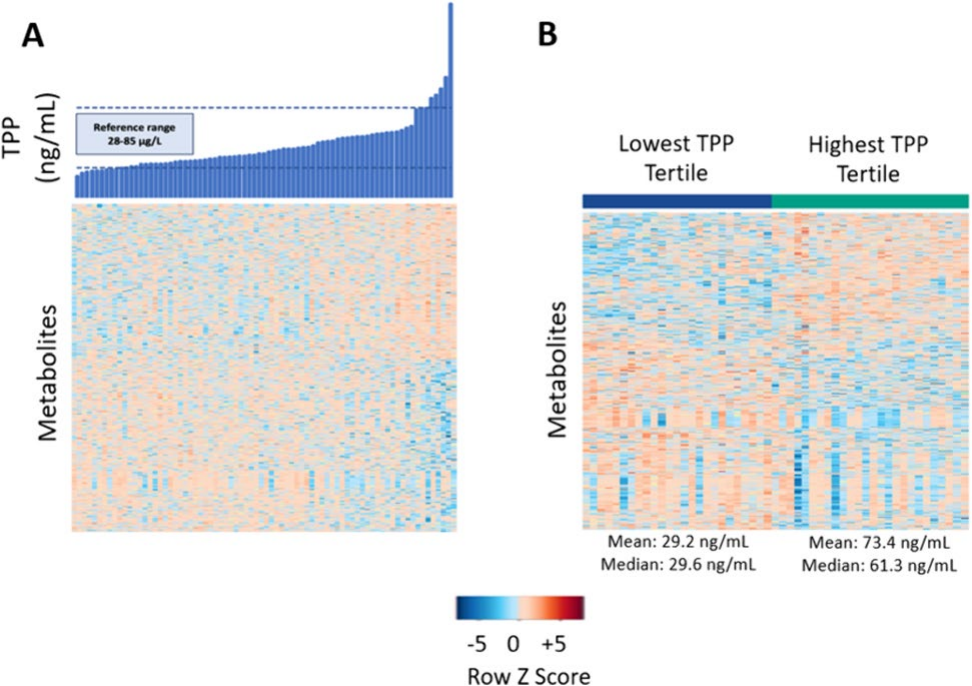
One-way unsupervised hierarchical clustering analysis (HCA) plot of significant metabolites associated with whole blood TPP concentrations at P< 0.05. Individual participant values are in blue (top) along the x-axis and linked metabolites for each participant are shown as color-coded rows along the y-axis. The normal range for whole blood TPP concentrations is shown with the dashed lines. The metabolite relationship to TPP levels reveals the distinct clusters of metabolites that are down-regulated as a function of whole blood TPP concentrations (blue coloration) in contrast to those up-regulated as a function of whole blood TPP (orange-red coloration). **B** One-way HCA of significant metabolites associated with the lowest versus the highest whole blood TPP concentration tertile at P < 0.05. The data show distinct metabolite clustering as a function of TPP concentration tertile. The mean and median values with standard deviations for whole blood TPP is shown. Color scheme representations are as outlined in A description

Features associated with whole blood TPP concentrations were plotted using one-way Hierarchical Cluster Analysis (HCA). Features that were increased or decreased in relation to TPP levels reveal distinct clustering and regulation (Fig. 2A). Metabolomic feature clustering was more distinct in the tertile analysis shown in Fig. 2B.

### Pathway HRM analysis

**Fig. 3A:**
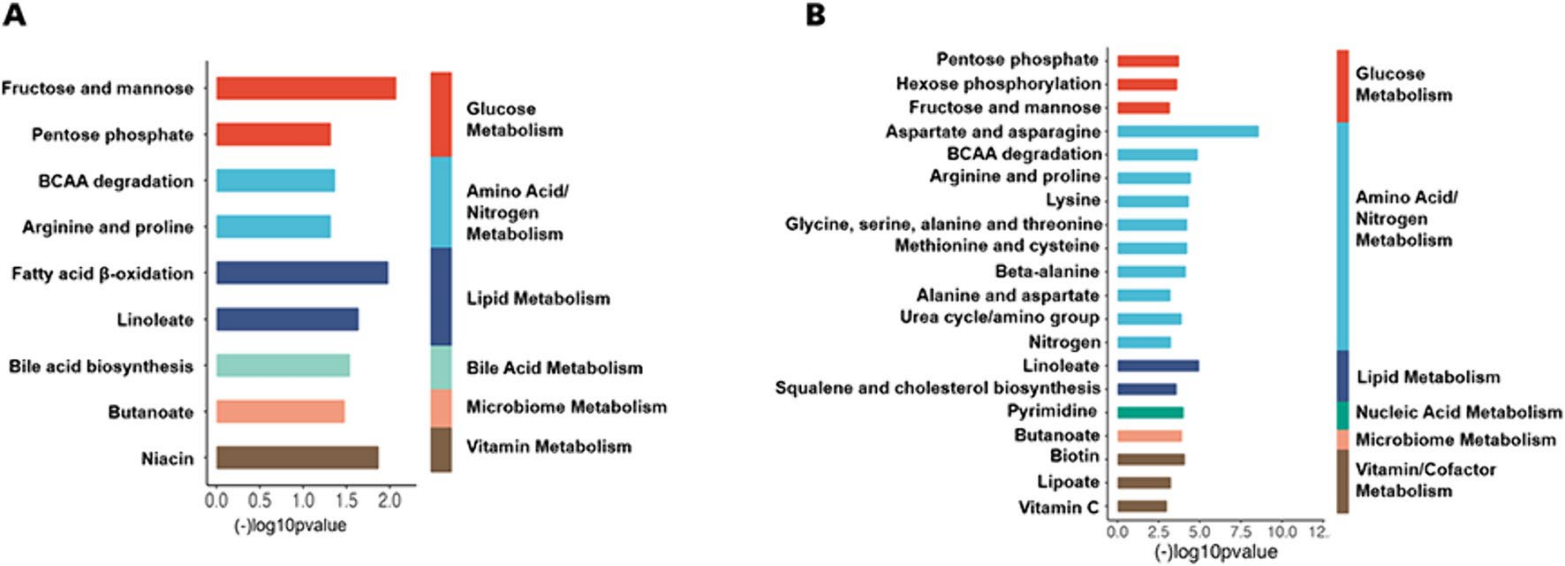
Metabolic pathway analysis for the whole blood TPP concentration metabolome-wide association analysis (MWAS) in the 76 critically ill study participants upon admission to the ICU. Significant pathways spanned glucose metabolism, amino acid and nitrogen metabolism, lipid metabolism, bile acid metabolism, microbiome metabolism, and vitamin metabolism, respectively. Specific pathways enriched in association with TPP levels were determined using the mummichog pathway enrichment analysis program (P < 0.05).** B** Metabolic pathway analysis comparing the lowest versus highest TPP whole blood concentration tertile. Numerous metabolic pathways, spanning amino acid/nitrogen metabolism, glucose metabolism, and vitamin/cofactor metabolism were significantly different comparing the lowest tertile versus the highest TPP concentration tertile (P < 0.05)

Metabolites associated with whole blood TPP concentrations according to MWAS analysis were significantly enriched in nine metabolic pathways (Fig. 3A). The enriched pathways included glucose metabolism (fructose and mannose, pentose phosphate pathway), amino acid and nitrogen metabolism (BCAA degradation, arginine, and proline), lipid metabolism (fatty acid β-oxidation and linoleate metabolism), bile acid metabolism (bile acid biosynthesis), microbiome (butanoate metabolism), and vitamin metabolism (niacin metabolism), respectively.

Metabolites that differed between the lowest and highest TPP tertiles were enriched in 20 metabolic pathways (Fig. 3B). Most of the significant pathways were related to amino acid/nitrogen metabolism (e.g., aspartate and asparagine, BCAA degradation, arginine and proline, lysine, and others), glucose metabolism (pentose phosphate, hexose phosphorylation, fructose, and mannose), and vitamin/cofactor metabolism (biotin, lipoate and vitamin C).

The relationship between whole blood TPP concentrations and selected significant metabolites from enriched pathways found using MWAS analysis are shown in Table 2. There is a significant negative correlation between TPP concentrations and plasma gluconate, deoxyuridine and linoleate levels (p = 0.043, P = 0.005 and P = 0.034 respectively). We also focused on the TCA cycle-related metabolites pyruvate, citrate/isocitrate, succinate, α-ketoglutarate, and malate, which we have previously validated using our HRM workflow (Liu et al., 2020). None of these TCA cycle metabolites in plasma were linked to TPP levels. Selected metabolites that differentiate patients in the lowest TPP tertile from those in the highest TPP tertile are listed in Supplemental Table 5.

**Fig. 4A:**
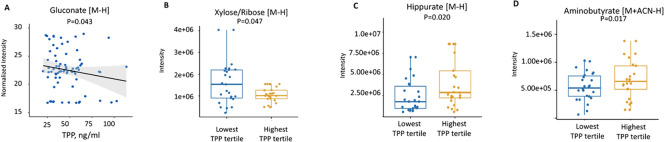
Regression plot of gluconate showing the association with whole blood TPP concentrations from the metabolome-wide association analysis (MWAS). **B–D** Box plots of selected metabolites that were altered in the lowest whole blood TPP concentration tertile compared to highest TPP tertile

**Fig. 5:**
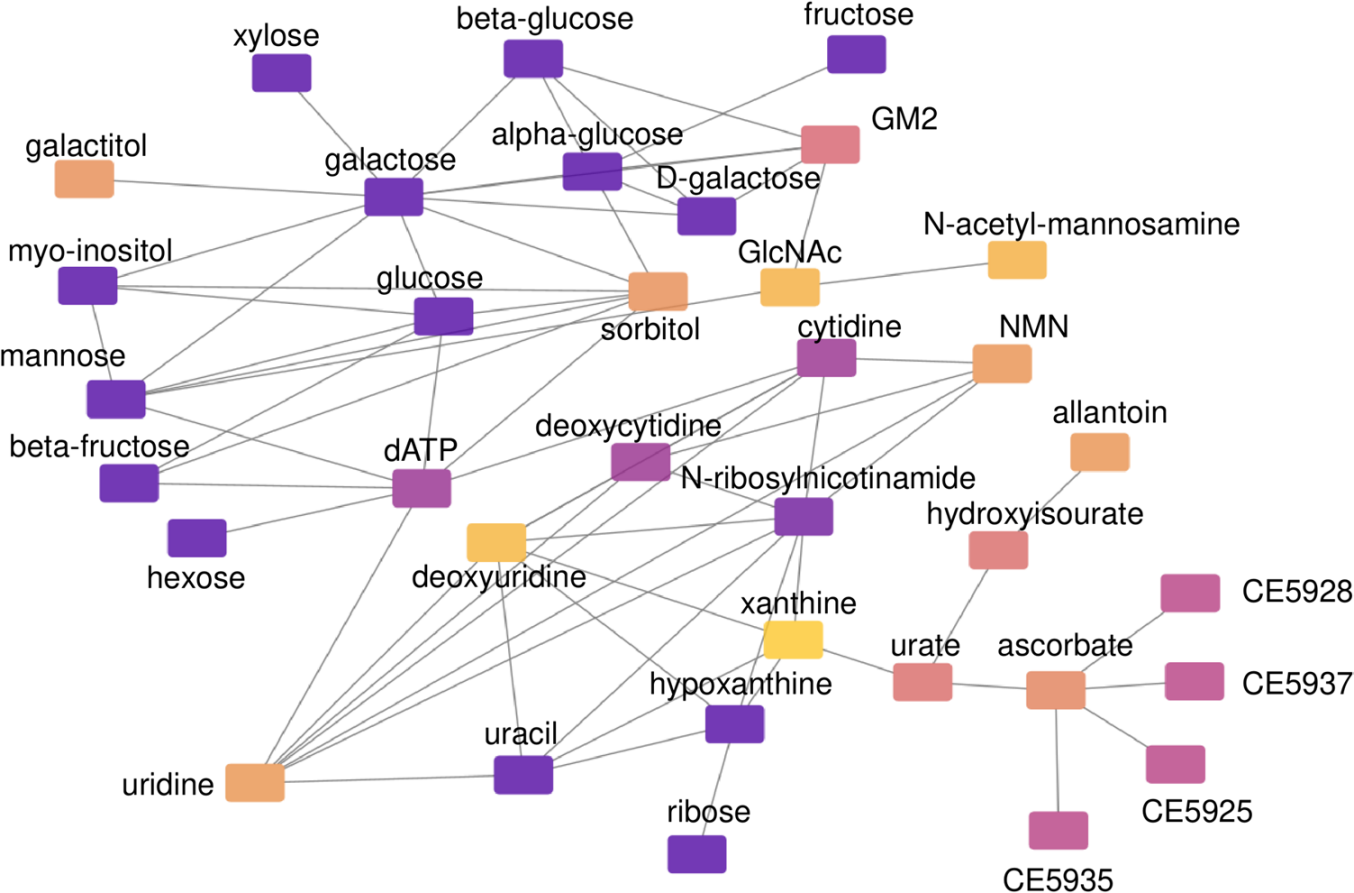
Metabolic network module of metabolites associated with whole blood TPP concentrations. Nodes represent metabolites annotated by mummichog that are correlated with other connected metabolites. Multiple carbohydrate-related metabolites are a central part of the network module. All metabolites are significantly related to whole blood TPP levels at P < 0.05, ranging from P = 0.003 (yellow) to P =  0.043 (purple). CE5925, CE5928, CE5935, & CE5937 are annotated as isomers of peroxy-eicosatetraenoate according to https://metabolicatlas.org/. *GM2 * N-Acetyl-D-galactosaminyl-(N-acetylneuraminyl)-D-galactosyl-D-glucosylceramide, *NMN* nicotinamide mononucleotide

Plots in Fig. 4A and 4B show the relationship between TPP and two metabolites involved in the pentose phosphate pathway, gluconate (P = 0.043) and xylose/ribose (P = 0.047), which are both negatively associated with TPP levels. The association between gut microbiome metabolites hippurate and aminobutyrate and TPP are shown in Fig. 4C and 4D (P = 0.02 and P 0.017, respectively). Both metabolites were positively associated with TPP (increased in the highest TPP tertile). Metabolites that were correlated with each other across multiple pathways can be seen in the network/module plot in Fig. 5. The metabolites that were significantly linked as a function of whole blood TPP levels were largely carbohydrates, but the module also included ascorbic acid and glycosylated nucleosides (e.g., uridine, cytidine).

## Discussion

In this pilot study, high-resolution plasma metabolomics analysis showed that whole blood TPP concentrations are significantly associated with metabolites and pathways related to energy (gluconate, deoxyribose) amino acid (arginine, and proline), lipid (lineolate) and gut microbiome metabolism (hippurate, aminobutyrate) in adult critically ill patients.

Our review of the literature did not reveal any prior papers linking thiamine status with metabolism in critical illness using metabolomics analysis. Thiamine plays an essential role in the energy metabolism in the human body (Frank, 2015; Whitfield et al., 2018). It acts as a co-factor for pyruvate dehydrogenase in glycolysis, alpha-ketoglutarate dehydrogenase in the TCA cycle and transketolase in the pentose phosphate pathway, among its more important functions (Frank, 2015; Whitfield et al., 2018).

We found that thiamine status, as assessed by whole blood TPP concentrations were significantly related to two key metabolites involved in function of the pentose phosphate pathway, namely, the hydrogen adducts (M-H) of gluconate and xylose/ribose, which were both increased in individuals with lower TPP concentrations.

The pentose phosphate pathway is critical for a variety of key biochemical functions, including maintenance of carbon homoeostasis, generation of precursors for nucleotide and amino acid biosynthesis, and protection from oxidative stress via production of NADPH (Krüger et al., 2011; Nalos et al., 2016; Sigurdsson et al., 2022) from the reduced form of nicotinamide adenine dinucleotide phosphate (NADP +). Thus, our data suggests the possibility that low TPP concentrations may contribute to decreases in transketolase activity within the pentose phosphate pathway that, in turn, may disrupt energy generation via the tightly linked pathways of glycolysis and the TCA cycle (Frank, 2015; Whitfield et al., 2018).

Thiamine deficiency has been previously found to disrupt energy metabolism in rats by affecting glucose transport and fatty acid β-oxidation in mitochondria (Gralak et al., 2019). In addition, the peroxisomal α-oxidation of 3-methyl fatty acids has been shown to be dependent on TPP (Casteels et al., 2003). However, to our knowledge, no data to date has associated thiamine status with fatty acid oxidation or lipid metabolism in humans. In the current study, pathway analysis and targeted assessment of specific metabolic features (see Table 2 and Supplemental Table 5, Figs. 3 and 4) identified fatty acid β-oxidation, linoleate metabolism, and squalene and cholesterol biosynthesis as lipid-related metabolic pathways linked to thiamine status. These unexpected hypothesis-generating data suggest the possibility that thiamine nutriture may be involved in lipid metabolism in critically ill adults and should be further explored in larger, prospective cohorts.

Thiamine also plays an important role in amino acid metabolism. TPP is a critical coenzyme for branched-chain α-ketoacid dehydrogenase (BCKDH), which is essential for the catabolism of branched-chain amino acids (BCAA) and subsequent utilization in the TCA cycle, among other functions (Depeint et al., 2006; Duran & Wadman, 1985). Our study revealed significant associations of TPP levels with numerous amino acid metabolic pathways, including BCAA metabolism, arginine and proline metabolism, aspartate and asparagine metabolism methionine and cysteine metabolism, and the urea cycle (Fig. 3). As shown in Supplemental Table 5, the TPP concentrations were negatively associated with asparagine, lysine and valine.

Our data expand the metabolomic results of several studies in critically ill patients with or without sepsis where a variety of alterations in amino acids and amino acid pathways have been identified, but in whom thiamine status was not determined (Chen et al., 2022; Ohlstrom et al., 2022; Rogers et al., 2014). Given the critical role of amino acid-derived metabolites in the TCA cycle (e.g., via α-ketoglutarate, oxaloacetate, succinate, etc.), it is possible that the broad impact of TPP status on amino acid metabolism we observed may impact energy generation indirectly via the TCA cycle. Further translational studies are needed to confirm such an effect, particularly since we did not observe any direct impact of TPP status on the TCA cycle metabolites pyruvate, citrate/isocitrate, succinate, α-ketoglutarate or malate. It is also possible, though speculative, that depletion of TPP has adverse effects on skeletal muscle or other tissues which utilize amino acids or are highly involved in amino acid metabolism, a hypothesis that requires further study.

It is known that the gut microbiota can generate TPP in mice, although the nutritional significance of this is unclear (Sabui et al., 2021). Studies have shown that the gut microbiome composition and diversity is disrupted in human critical illness (Lamarche et al., 2018; Haak et al. 2021). Therefore, it is possible that these disruptions may contribute to circulating TPP concentrations. Another study found that hypoxia in human colonic epithelial cells inhibited colonic uptake of gut microbiota generated TPP (Sabui et al., 2022). We did not study the gut microbiome directly in our participants, but we observed that two major gut microbiome-derived metabolites, hippurate and aminobutyrate, were each decreased in patients in the lowest TPP tertile, and the butanoate (butyrate) metabolic pathway was significantly affected by TPP status (Figs. 3 and 4C–D). Thus, it is possible that thiamine status may be related to gut microbiome dysregulation by currently unknown mechanisms. Future studies should further explore the possible link between TPP status and gut microbiome in humans.

Using untargeted high-resolution metabolomics, we detected over 20,000 metabolomic features and were able to look for associations of these features with whole blood TPP in critically ill patients. We used our established computational methods to predict how these features are related to metabolic changes in patients relative to thiamine status (Jones, 2016; Uppal et al., 2016). While we consider this methodology to be a strength of our study, we are aware that there were also several limitations of this pilot study. These include the relatively small sample size and the cross-sectional study design, which precludes cause and effect relationships between thiamine status and the metabolic associations observed. Our study population was derived from a single Turkish medical center and did not include non-critically ill control participants or a control group of critically ill patients not receiving furosemide treatment. The purpose of our study was to associate thiamine status with metabolomic profiles for the first time using HRM. We do not have sufficient power to the link thiamine status and metabolomic profiles with clinical outcomes. That question would be important for subsequent larger prospective studies that could be informed by our data. We believe that our data, as presented, are valid to define the relationship of thiamine status on the plasma metabolome in our cohort. Future larger studies would ideally obtain fasted blood samples, but this is difficult in many ICU patients who are receiving continuous nutrition support.

Another limitation is that the sample size did not allow comparison between different primary reasons for ICU admission or nutritional status of participants. Our data are novel in the context of thiamine status associations with specific metabolic pathways in critical illness using plasma high-resolution metabolomics; however, further studies are needed to determine whether thiamine status is linked to these specific pathways in other disease states or in different types of critically ill patients. To our knowledge, this is the first study in human critical illness to link thiamine nutritional status with systemic metabolism using metabolomics analysis. Our rigorous plasma HRM methods are state-of-the art and all samples are analyzed in triplicate with internal standards and are well validated in the literature (Jones, 2016). Data were also adjusted for illness severity (APACHE II score) on the day plasma for HRM was obtained. We found that whole blood TPP levels are linked to metabolic pathways and metabolites (e.g., lipid metabolism, gut microbiome) not generally considered in thiamine metabolism and therefore are hypothesis-generating for subsequent confirmatory and translational mechanistic studies.

## Conclusions

Using a hybrid approach of targeted and untargeted plasma metabolomics, we found changes in numerous metabolites and metabolic pathways associated with energy metabolism, amino acid metabolism, lipid metabolism and gut microbiome metabolism as a function of whole blood TPP concentrations in critically ill adults. Plasma HRM as utilized in this study provides new understanding of how the status of a single micronutrient, TPP, may impact whole body metabolism.

### Supplementary Information

Below is the link to the electronic supplementary material.

Supplementary file1 (PDF 5943 KB)

## Data Availability

Data available upon request.
