# Supplementary material for: High-resolution plasma metabolomics and thiamine status in critically Ill adult patients
Source: Metabolomics. 2024 Jul 27;20(4):83. doi: 10.1007/s11306-024-02144-9 (PMC11283406; doi:10.1007/s11306-024-02144-9)
Supplement: Supplementary file 1 — Supplementary file1 (PDF 5943 KB) [file 11306_2024_2144_MOESM1_ESM.pdf]

Supplemental Digital Content

High-Resolution Plasma Metabolomics and Thiamine Status in Critically Ill Adult Patients

Kursat Gundogan, MD; Mary M. Nellis, PhD; Nurhayat T. Ozer, PhD; Serap S. Ergul, PhD; Gulsah G. Sahin, PhD; Sahin Temel, MD; Recep C. Yuksel, MD; Sami Teeny, MS; Jessica A. Alvarez, PhD, RD; Murat Sungur, MD; Dean P. Jones, PhD; and Thomas R. Ziegler, MD

Table of Contents

Supplemental Table 1.. Page 4

Supplemental Table 2.. Page 22

Supplemental Table 3.. Page 49

Supplemental Table 4.. Page 68

Supplemental Table 5.. Page 97

Supplemental Table 1. Significant metabolic features linked to TPP concentrations from the C18 negative ESI column: MWAS

m/z	Time (sec)	Differential expression rank	P value
87.045	152	37	3.52E-03
89.9984	120	588	4.50E-02
92.0141	293	602	4.59E-02
92.8397	207	88	8.07E-03
93.0457	288	494	3.88E-02
96.0454	287	562	4.32E-02
100.9645	146	360	2.94E-02
105.0334	281	302	2.32E-02
111.0199	297	571	4.38E-02
112.0039	297	123	9.94E-03
112.0736	261	534	4.15E-02
112.9991	295	323	2.57E-02
115.0399	269	374	3.04E-02
123.0086	34	439	3.48E-02
125.097	158	62	5.59E-03
127.0011	101	641	4.80E-02
127.0398	164	329	2.62E-02
139.0261	17	153	1.16E-02
141.0554	161	406	3.25E-02
142.0954	31	300	2.31E-02
144.0315	291	220	1.68E-02
149.0244	296	473	3.68E-02
149.0547	191	471	3.67E-02
150.0018	41	612	4.66E-02
151.0148	293	276	2.12E-02
151.0705	282	381	3.08E-02
153.0168	28	225	1.70E-02
153.0668	286	50	4.73E-03
154.0507	156	269	2.05E-02
157.0156	296	191	1.48E-02
157.1232	49	365	2.96E-02
158.0821	151	154	1.16E-02

162.0502	288	351	2.88E-02
163.0343	298	594	4.54E-02
163.1127	172	306	2.33E-02
166.0223	31	155	1.17E-02
171.0313	287	495	3.88E-02
172.088	282	349	2.83E-02
172.9609	149	51	4.74E-03
173.058	291	556	4.28E-02
174.0309	283	432	3.40E-02
176.9291	117	324	2.57E-02
180.0441	296	627	4.75E-02
181.0004	18	209	1.61E-02
182.0319	17	305	2.33E-02
183.0159	17	240	1.80E-02
184.0194	18	294	2.27E-02
185.082	22	380	3.08E-02
188.0771	295	603	4.60E-02
189.0893	284	164	1.24E-02
192.0527	290	38	3.60E-03
192.089	282	317	2.48E-02
195.0511	19	575	4.39E-02
195.0525	291	68	6.01E-03
196.0544	19	581	4.42E-02
196.9034	268	44	4.34E-03
197.0663	286	168	1.27E-02
197.083	226	377	3.07E-02
197.0971	165	105	9.15E-03
199.0268	16	170	1.30E-02
200.0467	293	536	4.16E-02
201.0714	19	567	4.35E-02
201.8022	20	569	4.37E-02
202.0816	287	525	4.12E-02
203.1052	287	255	1.97E-02
206.0573	288	60	5.40E-03
207.0427	294	661	4.94E-02
209.0043	126	334	2.72E-02
209.1548	155	390	3.14E-02
210.9317	32	231	1.71E-02
213.0533	288	353	2.89E-02

213.0615	296	9	1.18E-03
214.1534	295	144	1.09E-02
217.0365	21	16	1.89E-03
218.1048	230	396	3.18E-02
219.1002	294	304	2.32E-02
219.9535	28	617	4.70E-02
221.0247	17	270	2.06E-02
222.0786	230	278	2.13E-02
222.1137	21	598	4.56E-02
222.1625	203	399	3.21E-02
223.0978	157	204	1.53E-02
227.2084	182	643	4.81E-02
228.1257	285	375	3.05E-02
231.0335	21	445	3.50E-02
234.1	280	112	9.28E-03
234.9813	28	658	4.93E-02
235.9849	35	18	2.10E-03
236.078	19	94	8.39E-03
236.0945	235	282	2.16E-02
237.1137	294	110	9.25E-03
239.0934	287	343	2.79E-02
239.1292	22	651	4.89E-02
241.9998	25	274	2.10E-02
242.069	17	414	3.33E-02
242.08	289	424	3.36E-02
242.1245	294	465	3.61E-02
243.197	24	47	4.42E-03
245.0795	288	218	1.66E-02
247.9849	30	336	2.73E-02
249.0248	17	271	2.07E-02
249.9641	50	70	6.07E-03
252.1088	284	179	1.39E-02
254.08	289	387	3.11E-02
254.0883	294	590	4.50E-02
254.1246	291	310	2.40E-02
255.1424	237	99	8.76E-03
255.9715	170	480	3.75E-02
256.0549	16	192	1.48E-02
257.1218	153	159	1.20E-02

257.9976	132	522	4.11E-02
259.1319	287	141	1.08E-02
260.1772	117	114	9.48E-03
261.2228	214	291	2.24E-02
263.0455	20	80	7.14E-03
263.1057	290	497	3.91E-02
263.2387	235	247	1.88E-02
264.8371	169	672	5.00E-02
268.1615	129	428	3.38E-02
273.0924	21	481	3.76E-02
274.0855	289	670	4.98E-02
277.1048	293	517	4.03E-02
280.0089	26	152	1.15E-02
280.1159	295	243	1.81E-02
280.9276	151	483	3.77E-02
280.975	19	669	4.97E-02
282.2717	246	350	2.83E-02
286.0654	294	319	2.50E-02
286.9542	156	91	8.26E-03
286.9821	44	338	2.74E-02
286.9933	24	322	2.54E-02
287.1507	283	205	1.53E-02
287.2235	25	256	1.98E-02
289.0631	295	167	1.26E-02
289.0634	20	413	3.33E-02
289.2177	188	145	1.11E-02
290.9981	80	389	3.13E-02
291.1244	19	446	3.50E-02
291.1592	125	293	2.25E-02
292.0969	16	214	1.64E-02
292.1154	295	251	1.94E-02
292.2003	162	348	2.82E-02
292.9223	28	341	2.77E-02
292.9464	263	132	1.03E-02
293.0915	294	325	2.58E-02
293.1401	171	527	4.13E-02
295.1154	295	514	4.02E-02
296.2318	217	165	1.24E-02
298.1429	284	53	4.79E-03

300.9324	30	460	3.58E-02
304.1156	297	637	4.79E-02
305.0092	89	412	3.32E-02
305.1165	293	107	9.18E-03
308.1638	182	420	3.34E-02
308.87	29	552	4.25E-02
309.0687	30	582	4.44E-02
309.12	20	631	4.77E-02
310.1261	22	357	2.91E-02
310.2393	218	520	4.06E-02
311.1635	182	496	3.90E-02
313.2394	217	624	4.75E-02
314.9056	30	654	4.91E-02
315.1113	289	332	2.69E-02
315.182	273	120	9.67E-03
315.2367	143	611	4.66E-02
315.2369	259	57	5.29E-03
315.2547	265	640	4.79E-02
319.0345	280	217	1.66E-02
320.1108	287	633	4.78E-02
324.2451	190	560	4.31E-02
325.13	167	646	4.86E-02
325.1935	182	642	4.81E-02
325.2391	214	667	4.96E-02
326.1111	57	262	2.02E-02
327.9961	24	346	2.81E-02
329.1766	25	568	4.36E-02
330.0288	16	238	1.78E-02
330.9566	186	318	2.50E-02
331.9958	28	466	3.61E-02
332.0041	118	49	4.45E-03
333.2075	129	354	2.89E-02
334.9927	25	616	4.68E-02
335.1903	139	622	4.73E-02
336.9206	42	382	3.08E-02
337.2372	151	587	4.50E-02
338.976	164	7	1.01E-03
339.2356	234	72	6.19E-03
339.255	246	222	1.68E-02

340.2378	226	574	4.38E-02
341.0028	298	58	5.33E-03
341.3066	202	546	4.21E-02
343.1563	138	666	4.96E-02
343.259	193	493	3.88E-02
344.1377	283	257	1.99E-02
344.1952	127	388	3.12E-02
344.9799	118	548	4.23E-02
346.8734	134	28	2.66E-03
347.19	167	619	4.71E-02
349.1791	168	464	3.60E-02
352.9042	32	104	9.10E-03
353.2341	29	180	1.40E-02
354.9254	60	195	1.49E-02
355.1409	194	249	1.91E-02
360.1957	217	140	1.08E-02
360.9437	56	653	4.89E-02
361.1879	272	150	1.14E-02
361.2241	162	500	3.93E-02
361.2373	213	586	4.49E-02
362.2401	217	29	2.79E-03
363.0435	266	131	1.03E-02
363.1958	217	156	1.17E-02
364.1986	213	133	1.03E-02
364.986	273	632	4.78E-02
365.1461	15	335	2.73E-02
366.9128	37	171	1.30E-02
367.1281	155	290	2.23E-02
369.1655	24	272	2.08E-02
371.0086	165	142	1.09E-02
371.1213	227	409	3.30E-02
371.1717	24	34	3.32E-03
371.2449	23	376	3.05E-02
371.9884	26	273	2.08E-02
372.1744	25	116	9.62E-03
375.0368	18	544	4.18E-02
375.1953	190	662	4.95E-02
375.258	176	615	4.68E-02
377.2127	214	138	1.06E-02

378.0042	157	442	3.49E-02
378.213	213	81	7.17E-03
381.302	195	634	4.78E-02
382.2436	168	63	5.63E-03
382.9567	122	487	3.81E-02
383.0299	33	400	3.21E-02
383.056	119	363	2.95E-02
384.8696	39	279	2.15E-02
387.0124	132	584	4.47E-02
387.1857	21	146	1.11E-02
387.2584	174	519	4.05E-02
389.1817	265	128	1.02E-02
389.276	233	299	2.30E-02
390.3138	192	8	1.08E-03
391.0297	17	600	4.57E-02
391.2356	186	147	1.12E-02
393.2295	160	199	1.51E-02
393.2503	151	176	1.38E-02
395.1064	26	312	2.44E-02
396.2582	176	79	6.78E-03
396.9724	122	510	3.99E-02
397.2371	219	591	4.51E-02
397.9761	117	456	3.55E-02
398.2378	159	117	9.63E-03
398.3366	191	241	1.81E-02
398.9893	120	228	1.70E-02
401.1831	269	504	3.95E-02
402.2407	160	281	2.16E-02
403.2162	146	71	6.10E-03
403.2463	142	462	3.59E-02
403.2609	197	364	2.95E-02
405.2296	31	139	1.06E-02
406.2177	146	46	4.41E-03
406.9102	156	303	2.32E-02
406.9779	29	436	3.46E-02
407.2581	195	22	2.30E-03
409.3083	138	503	3.95E-02
411.17	167	212	1.62E-02
411.9916	125	411	3.32E-02

412.1895	21	321	2.52E-02
413.8641	46	368	3.00E-02
417.3025	127	163	1.22E-02
423.1564	180	23	2.36E-03
423.3491	228	148	1.13E-02
424.2445	128	528	4.13E-02
427.0194	125	532	4.14E-02
427.0324	39	563	4.32E-02
428.0526	22	250	1.93E-02
430.2974	22	330	2.63E-02
431.1922	195	160	1.20E-02
431.2209	127	479	3.75E-02
431.302	133	233	1.73E-02
433.3012	196	258	1.99E-02
434.9732	31	425	3.37E-02
437.2406	226	440	3.49E-02
439.2824	197	498	3.92E-02
442.2094	161	242	1.81E-02
443.9394	141	178	1.39E-02
444.9742	122	649	4.89E-02
448.2648	217	405	3.25E-02
448.3528	221	403	3.25E-02
449.1524	17	435	3.46E-02
451.0455	173	518	4.05E-02
451.1808	44	489	3.84E-02
452.2783	203	261	2.02E-02
453.2347	162	313	2.45E-02
453.3233	187	371	3.02E-02
453.3595	209	595	4.55E-02
453.992	27	143	1.09E-02
454.2844	201	524	4.12E-02
456.9837	31	234	1.74E-02
459.2142	216	295	2.27E-02
462.9322	125	101	9.01E-03
462.9936	27	639	4.79E-02
463.2355	22	235	1.75E-02
465.251	21	173	1.30E-02
465.4324	300	207	1.57E-02
467.2663	164	211	1.62E-02

467.2671	22	578	4.41E-02
467.2874	200	77	6.64E-03
471.2973	166	404	3.25E-02
471.3701	196	185	1.42E-02
471.9828	134	429	3.38E-02
472.8754	36	474	3.68E-02
475.1847	212	459	3.57E-02
477.2904	264	564	4.32E-02
478.9058	141	177	1.39E-02
479.2318	22	135	1.04E-02
479.2978	209	395	3.18E-02
479.3039	282	392	3.16E-02
480.3261	284	55	5.23E-03
481.1029	17	327	2.60E-02
482.2499	21	485	3.80E-02
483.0464	81	660	4.94E-02
484.3682	178	151	1.14E-02
484.8204	49	655	4.91E-02
484.9093	59	201	1.52E-02
487.2556	192	315	2.46E-02
489.2308	178	35	3.35E-03
489.2351	19	111	9.25E-03
489.9655	105	161	1.22E-02
492.3252	167	461	3.58E-02
492.3639	255	129	1.02E-02
492.9393	148	415	3.33E-02
493.0011	30	419	3.34E-02
493.3032	269	509	3.98E-02
494.9697	121	613	4.66E-02
495.5507	16	224	1.68E-02
496.0498	176	288	2.21E-02
497.2613	175	43	4.12E-03
497.2772	160	573	4.38E-02
497.9131	55	618	4.70E-02
501.0037	38	416	3.33E-02
501.235	169	162	1.22E-02
501.3073	170	490	3.85E-02
504.8573	148	592	4.52E-02
505.2304	149	605	4.61E-02

505.3548	124	444	3.50E-02
506.8861	163	215	1.64E-02
508.3427	228	127	1.02E-02
509.2879	143	115	9.48E-03
509.2997	197	629	4.77E-02
509.3129	156	538	4.17E-02
509.3337	180	664	4.96E-02
509.3838	136	66	5.89E-03
510.2929	143	268	2.03E-02
510.3358	180	579	4.41E-02
511.2935	150	448	3.51E-02
512.3669	158	119	9.65E-03
513.3046	196	275	2.11E-02
516.0268	17	108	9.23E-03
517.2559	154	40	3.95E-03
518.8733	46	347	2.81E-02
520.2669	202	391	3.14E-02
521.0357	46	580	4.42E-02
524.8455	157	122	9.91E-03
524.8471	34	663	4.95E-02
524.8957	54	565	4.34E-02
525.2408	217	583	4.44E-02
527.0219	157	606	4.62E-02
529.2753	225	130	1.03E-02
533.4198	68	550	4.24E-02
533.9385	77	539	4.17E-02
535.3157	209	187	1.43E-02
536.7819	154	529	4.14E-02
536.9949	74	283	2.16E-02
537.2544	167	264	2.02E-02
537.3354	278	431	3.40E-02
537.374	296	540	4.18E-02
538.3158	207	280	2.16E-02
538.3343	238	316	2.47E-02
539.3197	205	476	3.72E-02
539.3238	283	407	3.27E-02
539.9798	121	340	2.75E-02
540.2751	182	668	4.97E-02
540.348	291	48	4.43E-03

540.8819	175	434	3.44E-02
541.2162	212	42	4.10E-03
541.2674	21	61	5.45E-03
541.3752	187	287	2.19E-02
541.9757	111	31	3.01E-03
542.2704	22	252	1.95E-02
542.8775	60	352	2.88E-02
543.1595	130	297	2.27E-02
545.4004	202	359	2.93E-02
545.9221	62	4	5.09E-04
545.9496	76	551	4.25E-02
546.9394	63	289	2.21E-02
548.8437	48	244	1.81E-02
549.8391	52	292	2.24E-02
551.3483	169	408	3.29E-02
555.443	297	121	9.74E-03
556.2688	178	183	1.41E-02
556.3543	278	427	3.37E-02
556.8592	149	452	3.53E-02
557.2629	190	656	4.91E-02
558.3718	147	482	3.76E-02
558.4533	228	561	4.31E-02
561.4806	218	450	3.52E-02
562.868	137	486	3.80E-02
566.8803	57	172	1.30E-02
567.3528	190	393	3.16E-02
567.3878	188	488	3.84E-02
570.3358	151	597	4.56E-02
570.9227	181	557	4.30E-02
571.3471	201	339	2.74E-02
572.7803	38	604	4.61E-02
572.9265	89	76	6.42E-03
573.4301	228	648	4.87E-02
574.432	228	657	4.93E-02
575.0098	35	186	1.43E-02
575.8137	57	621	4.72E-02
576.8927	40	422	3.35E-02
578.3224	224	530	4.14E-02
578.8061	52	10	1.22E-03

579.3819	131	625	4.75E-02
582.8602	56	100	8.88E-03
582.8709	136	344	2.80E-02
583.0067	39	585	4.48E-02
583.4687	235	227	1.70E-02
585.1007	190	531	4.14E-02
587.0024	125	566	4.35E-02
588.0021	131	109	9.24E-03
588.4415	76	361	2.95E-02
588.8621	185	213	1.63E-02
589.0252	42	470	3.65E-02
589.3616	154	437	3.47E-02
590.854	167	576	4.40E-02
590.9213	106	11	1.23E-03
592.2895	277	659	4.93E-02
594.8561	134	320	2.51E-02
594.9202	80	309	2.40E-02
596.3578	158	650	4.89E-02
597.4315	214	451	3.52E-02
598.4352	217	558	4.30E-02
599.2278	22	620	4.72E-02
599.4346	218	113	9.38E-03
599.9065	53	85	7.56E-03
602.3265	147	372	3.03E-02
604.8887	112	423	3.36E-02
606.3777	208	75	6.31E-03
608.8279	149	84	7.46E-03
609.4086	73	369	3.01E-02
609.8935	69	555	4.28E-02
610.8591	160	6	9.36E-04
611.4247	201	307	2.35E-02
611.9666	85	236	1.77E-02
612.5218	237	543	4.18E-02
612.7905	41	630	4.77E-02
614.8718	54	45	4.39E-03
615.2837	204	157	1.18E-02
617.9073	114	665	4.96E-02
617.9198	70	56	5.25E-03
618.2642	289	553	4.25E-02

618.2869	175	542	4.18E-02
618.805	50	537	4.17E-02
618.8382	171	410	3.31E-02
619.8682	56	3	4.64E-04
619.9325	75	41	4.07E-03
620.3697	60	206	1.56E-02
621.2146	260	545	4.19E-02
623.9247	146	326	2.60E-02
624.1339	16	74	6.28E-03
624.2575	182	67	5.95E-03
626.8819	142	521	4.10E-02
627.3763	177	636	4.79E-02
628.3611	226	158	1.19E-02
629.104	17	219	1.67E-02
629.3879	172	533	4.15E-02
629.92	80	455	3.55E-02
630.3433	292	570	4.38E-02
630.8587	67	333	2.71E-02
631.1075	17	203	1.53E-02
631.9145	84	166	1.26E-02
631.9233	85	221	1.68E-02
632.8749	62	402	3.23E-02
632.8944	169	599	4.56E-02
638.8612	179	454	3.55E-02
639.8647	146	523	4.11E-02
641.2605	186	547	4.22E-02
641.9605	94	36	3.45E-03
644.8959	113	443	3.50E-02
645.888	107	378	3.07E-02
645.9091	60	223	1.68E-02
645.9516	84	398	3.20E-02
646.3332	218	601	4.57E-02
646.823	157	174	1.34E-02
647.3665	156	149	1.14E-02
648.5218	211	508	3.98E-02
648.8189	49	103	9.07E-03
648.8588	114	259	2.01E-02
648.9121	70	189	1.45E-02
652.0177	94	182	1.41E-02

652.9034	66	505	3.96E-02
653.4723	240	301	2.32E-02
653.5198	217	577	4.40E-02
655.1606	211	202	1.52E-02
661.9658	83	535	4.16E-02
662.3679	190	296	2.27E-02
663.3643	171	484	3.77E-02
665.4212	214	628	4.76E-02
668.9063	103	593	4.53E-02
670.3315	158	198	1.49E-02
671.9206	90	469	3.65E-02
672.0639	16	645	4.83E-02
672.8965	117	245	1.85E-02
672.9176	102	513	4.02E-02
673.3393	283	345	2.81E-02
674.0351	15	286	2.18E-02
674.9694	130	216	1.64E-02
675.2324	279	96	8.59E-03
675.8668	58	24	2.43E-03
675.9043	104	169	1.27E-02
678.397	208	210	1.62E-02
678.9057	103	638	4.79E-02
679.4297	214	19	2.24E-03
679.4415	217	93	8.33E-03
680.8581	47	83	7.38E-03
681.0322	16	194	1.48E-02
683.8752	114	607	4.62E-02
683.8984	70	507	3.97E-02
685.8878	97	385	3.10E-02
688.8965	75	614	4.68E-02
690.9589	82	328	2.62E-02
691.8955	79	342	2.79E-02
692.5164	236	373	3.03E-02
692.5508	269	248	1.91E-02
692.8002	143	384	3.09E-02
694.8875	64	458	3.56E-02
696.0048	89	472	3.67E-02
697.0065	16	610	4.65E-02
697.9406	89	358	2.93E-02

699.8896	79	125	9.97E-03
701.5154	211	97	8.67E-03
702.7762	51	308	2.39E-02
703.0386	77	511	4.01E-02
703.9578	82	468	3.64E-02
708.9193	86	418	3.34E-02
711.9406	87	298	2.28E-02
714.8738	171	190	1.47E-02
717.4585	90	196	1.49E-02
717.5283	216	367	2.99E-02
718.4317	83	284	2.17E-02
720.4128	192	197	1.49E-02
722.9523	81	421	3.34E-02
724.9403	84	554	4.28E-02
726.8103	157	266	2.03E-02
726.8705	59	430	3.39E-02
727.8658	58	623	4.74E-02
727.919	70	54	5.08E-03
727.9552	106	136	1.04E-02
730.0075	90	477	3.73E-02
730.89	138	193	1.48E-02
732.3665	159	331	2.65E-02
736.9322	96	355	2.89E-02
738.4984	201	226	1.70E-02
742.8562	49	635	4.78E-02
742.8582	123	39	3.61E-03
743.9471	83	463	3.59E-02
746.2863	119	106	9.16E-03
747.3326	187	516	4.03E-02
749.9794	88	124	9.95E-03
752.3223	290	337	2.73E-02
754.886	60	449	3.52E-02
760.8711	68	501	3.94E-02
760.8799	99	25	2.44E-03
764.8042	137	229	1.71E-02
766.8237	171	95	8.42E-03
766.9176	87	626	4.75E-02
766.9834	127	90	8.26E-03
770.912	91	457	3.56E-02

772.927	90	265	2.03E-02
775.9781	87	311	2.41E-02
776.611	293	512	4.02E-02
776.7523	140	314	2.45E-02
776.9016	84	30	2.89E-03
781.572	211	652	4.89E-02
781.9182	81	15	1.60E-03
782.8492	178	181	1.40E-02
785.0321	79	492	3.88E-02
785.5842	185	277	2.13E-02
786.8829	57	263	2.02E-02
788.8225	131	82	7.24E-03
788.8517	110	65	5.87E-03
788.8567	114	13	1.41E-03
789.9088	92	1	2.20E-04
790.5421	293	491	3.87E-02
790.9241	97	401	3.21E-02
796.8952	94	379	3.08E-02
798.8884	102	246	1.86E-02
798.9519	92	27	2.46E-03
800.8462	58	285	2.17E-02
802.8882	151	208	1.57E-02
805.9683	90	32	3.23E-03
808.8101	155	175	1.36E-02
808.8746	58	433	3.41E-02
810.5416	203	78	6.65E-03
811.2914	292	188	1.44E-02
814.8568	74	33	3.30E-03
818.9366	86	386	3.10E-02
826.9297	90	126	9.99E-03
827.2552	291	506	3.96E-02
832.6133	195	52	4.77E-03
832.9749	79	230	1.71E-02
834.5096	200	92	8.33E-03
836.9894	131	267	2.03E-02
840.9387	102	475	3.69E-02
842.7823	138	260	2.02E-02
842.8928	102	98	8.73E-03
846.831	62	87	7.78E-03

846.9302	95	239	1.79E-02
847.6338	191	394	3.17E-02
850.597	196	356	2.90E-02
856.8638	105	572	4.38E-02
856.9409	96	59	5.35E-03
857.6081	193	526	4.12E-02
857.6215	188	515	4.03E-02
859.5155	197	253	1.96E-02
862.54	215	118	9.65E-03
869.6274	288	502	3.94E-02
870.8778	104	237	1.77E-02
878.585	198	644	4.82E-02
882.9738	97	549	4.24E-02
888.5281	179	559	4.30E-02
892.6224	197	5	7.89E-04
892.959	82	17	1.90E-03
895.0215	131	441	3.49E-02
899.6427	214	200	1.51E-02
899.6571	218	608	4.64E-02
900.5554	207	64	5.73E-03
902.5356	186	21	2.25E-03
904.9271	82	86	7.59E-03
904.9346	80	447	3.50E-02
906.863	60	609	4.64E-02
908.886	98	671	4.99E-02
918.5674	182	2	3.29E-04
918.5711	183	417	3.34E-02
921.9466	93	362	2.95E-02
922.9354	89	478	3.74E-02
924.6045	186	438	3.47E-02
926.566	179	134	1.03E-02
942.8715	72	89	8.19E-03
942.9412	91	69	6.03E-03
948.5463	185	589	4.50E-02
948.9896	93	541	4.18E-02
952.5738	182	596	4.55E-02
954.961	91	184	1.41E-02
973.1129	16	73	6.20E-03
974.9078	89	14	1.59E-03

980.5778	173	370	3.02E-02
997.5626	173	453	3.53E-02
998.5658	181	426	3.37E-02
1012.532	194	467	3.62E-02
1013.5403	193	12	1.30E-03
1015.8179	236	137	1.05E-02
1037.5367	187	647	4.86E-02
1037.806	217	102	9.06E-03
1038.7938	217	254	1.96E-02
1060.794	216	397	3.20E-02
1061.7962	217	499	3.93E-02
1062.8047	217	232	1.71E-02
1064.8209	214	366	2.98E-02
1065.8331	214	383	3.09E-02
1084.7937	216	26	2.45E-03
1088.828	218	20	2.25E-03

Raw p values < 0.05
ESI=electrospray ionization
MWAS= metabolome-wide association study
TPP = thiamine pyrophosphate

Supplemental Table 2. Significant metabolic features linked to TPP concentrations from the HILIC positive ESI column: MWAS

m/z	Time (sec)	Differential expression rank	P value
95.0493	293	136	7.89E-03
96.0445	205	646	3.25E-02
96.9611	289	180	9.96E-03
98.0714	286	3	9.07E-05
98.9615	297	164	9.20E-03
100.9569	295	470	2.39E-02
102.0551	91	501	2.55E-02
104.1072	41	107	6.45E-03
104.1091	107	29	1.80E-03
105.0336	28	495	2.51E-02
105.1101	42	980	4.76E-02
109.9892	290	160	9.08E-03
111.0805	24	589	3.02E-02
111.9685	71	248	1.35E-02
112.0475	11	892	4.39E-02
112.0588	285	383	1.99E-02
113.0599	294	681	3.43E-02
114.03	284	21	1.28E-03
114.9717	295	615	3.13E-02
115.0867	294	553	2.84E-02
115.9642	297	478	2.45E-02
116.972	297	332	1.75E-02
117.9596	297	142	8.38E-03
118.0823	70	580	2.99E-02
118.9675	297	299	1.57E-02
119.0832	69	833	4.14E-02
119.0896	71	609	3.11E-02
119.0923	69	582	2.99E-02
119.9572	295	521	2.69E-02
120.0237	298	911	4.47E-02
120.0655	91	204	1.14E-02
120.0832	61	68	4.00E-03

121.0761	12	742	3.69E-02
124.0393	27	503	2.57E-02
125.9723	295	275	1.46E-02
127.9668	290	552	2.84E-02
128.0819	273	223	1.22E-02
129.8313	152	704	3.53E-02
129.9513	290	341	1.77E-02
129.9542	290	960	4.68E-02
130.008	77	1018	4.91E-02
131.0929	154	412	2.11E-02
131.9699	292	588	3.02E-02
133.1082	65	854	4.24E-02
133.8247	158	764	3.79E-02
133.9747	295	391	2.02E-02
136.0216	75	708	3.55E-02
137.9875	290	238	1.30E-02
138.022	284	824	4.09E-02
138.0743	121	484	2.48E-02
139.0295	296	327	1.74E-02
139.9633	289	929	4.55E-02
139.988	297	533	2.75E-02
140.03	295	563	2.88E-02
140.0706	31	734	3.65E-02
140.0788	289	557	2.86E-02
140.9913	297	195	1.10E-02
140.9958	297	185	1.05E-02
141.0659	11	836	4.14E-02
141.0699	25	386	2.01E-02
141.1274	87	586	3.01E-02
141.9834	296	224	1.23E-02
142.1226	28	725	3.62E-02
142.9837	295	337	1.76E-02
142.9868	295	205	1.14E-02
142.9912	295	116	6.77E-03
143.959	295	258	1.40E-02
143.9809	297	213	1.18E-02
143.9969	293	536	2.76E-02
145.9544	294	369	1.92E-02
145.9805	294	169	9.47E-03

147.0636	62	751	3.76E-02
147.1128	9	230	1.25E-02
148.0474	45	703	3.52E-02
149.0138	296	442	2.26E-02
149.054	293	140	8.29E-03
150.0464	26	696	3.50E-02
151.039	20	214	1.18E-02
151.0866	277	541	2.79E-02
151.1481	83	110	6.53E-03
153.0546	80	124	7.19E-03
153.0546	25	561	2.87E-02
153.1022	275	385	2.00E-02
153.1273	70	963	4.69E-02
153.9492	96	811	4.02E-02
155.0702	24	843	4.16E-02
155.0855	22	629	3.18E-02
156.9906	297	247	1.35E-02
157.076	63	876	4.31E-02
157.9989	295	847	4.19E-02
158.0133	288	608	3.11E-02
158.1175	223	719	3.60E-02
158.986	296	485	2.48E-02
159.0651	275	352	1.81E-02
159.1168	24	92	5.13E-03
160.0757	289	702	3.52E-02
160.1332	40	810	4.01E-02
161.1002	70	613	3.12E-02
161.9696	295	109	6.46E-03
162.0454	298	760	3.77E-02
163.0294	294	953	4.64E-02
163.1117	83	822	4.07E-02
163.965	294	267	1.43E-02
165.9829	73	144	8.45E-03
167.0243	294	392	2.03E-02
167.982	293	127	7.34E-03
168.1383	273	862	4.27E-02
169.0356	55	206	1.14E-02
169.0608	11	338	1.77E-02
169.1011	24	770	3.81E-02

170.027	29	531	2.74E-02
170.0389	56	283	1.50E-02
170.9745	289	859	4.26E-02
170.9778	290	259	1.40E-02
170.9807	291	350	1.81E-02
171.0345	297	775	3.82E-02
174.0086	286	194	1.10E-02
174.0583	28	458	2.35E-02
174.9567	294	901	4.42E-02
177.0546	15	545	2.81E-02
177.0867	288	592	3.04E-02
177.1273	84	590	3.02E-02
178.0896	31	893	4.39E-02
179.0611	295	788	3.92E-02
179.1794	22	611	3.12E-02
180.0034	291	965	4.71E-02
180.056	295	319	1.69E-02
180.8945	8	918	4.50E-02
181.9794	296	763	3.79E-02
182.1257	286	599	3.06E-02
183.053	195	58	3.39E-03
183.1015	74	526	2.72E-02
184.0015	294	996	4.80E-02
184.1332	25	792	3.93E-02
184.1695	258	812	4.02E-02
184.9855	297	88	4.99E-03
185.0592	89	376	1.94E-02
185.0808	21	741	3.69E-02
185.9889	294	816	4.04E-02
186.0084	287	239	1.30E-02
186.981	297	134	7.82E-03
187.9906	96	782	3.88E-02
188.0498	295	902	4.42E-02
188.9786	293	208	1.15E-02
190.0291	297	119	7.03E-03
190.1074	286	499	2.53E-02
191.0243	295	767	3.80E-02
191.1027	294	690	3.48E-02
191.9535	294	63	3.78E-03

192.0243	92	643	3.24E-02
193.0277	93	568	2.89E-02
194.0326	297	683	3.44E-02
194.162	265	9	3.09E-04
195.1743	83	252	1.37E-02
196.0968	286	654	3.28E-02
196.8779	220	730	3.64E-02
198.1277	34	138	8.08E-03
208.1333	12	575	2.94E-02
209.013	294	899	4.40E-02
209.9645	294	714	3.59E-02
209.9743	295	827	4.11E-02
212.1726	280	225	1.24E-02
213.087	10	59	3.44E-03
213.1484	22	928	4.55E-02
214.0063	94	365	1.91E-02
216.1312	278	254	1.38E-02
218.0349	285	129	7.49E-03
218.1751	9	400	2.07E-02
219.9484	294	315	1.66E-02
221.1862	72	591	3.03E-02
223.1441	35	421	2.14E-02
224.1645	284	402	2.08E-02
224.2091	10	131	7.51E-03
225.0707	288	99	6.00E-03
225.0869	68	951	4.63E-02
225.2212	85	473	2.40E-02
226.1073	29	101	6.15E-03
227.9849	293	837	4.15E-02
228.1675	280	62	3.52E-03
229.9801	93	627	3.17E-02
231.0556	223	935	4.57E-02
231.1444	77	361	1.90E-02
232.0274	23	925	4.53E-02
232.9801	294	446	2.27E-02
233.0306	23	1005	4.83E-02
233.9541	90	1019	4.92E-02
235.1651	70	241	1.32E-02

236.1363	10	530	2.74E-02
236.1687	70	266	1.43E-02
238.2247	13	914	4.48E-02
239.1027	284	882	4.33E-02
241.1798	23	998	4.81E-02
241.2161	24	388	2.01E-02
241.9982	46	394	2.04E-02
241.9985	281	509	2.63E-02
242.2114	283	333	1.75E-02
246.0975	10	490	2.50E-02
246.9277	117	97	5.73E-03
247.9433	297	610	3.12E-02
248.0223	23	977	4.74E-02
249.202	76	937	4.58E-02
250.1439	273	964	4.70E-02
250.9559	298	820	4.05E-02
250.9923	296	427	2.16E-02
251.0228	298	310	1.64E-02
251.1641	21	844	4.16E-02
251.9393	297	805	4.00E-02
252.9972	281	335	1.76E-02
253.1646	233	389	2.02E-02
253.9355	296	73	4.48E-03
254.1023	29	861	4.27E-02
254.1832	10	823	4.08E-02
254.2195	88	581	2.99E-02
254.9938	297	600	3.07E-02
255.0046	297	496	2.51E-02
255.1227	14	587	3.01E-02
255.1227	289	184	1.05E-02
257.1384	15	263	1.42E-02
258.002	298	308	1.63E-02
258.1418	10	492	2.50E-02
259.9974	298	474	2.41E-02
260.0961	35	468	2.38E-02
261.1085	151	17	1.19E-03
261.901	128	974	4.73E-02
261.9591	294	465	2.37E-02

265.0837	138	210	1.16E-02
266.9448	92	766	3.80E-02
266.951	297	797	3.94E-02
266.9837	293	163	9.20E-03
268.0286	298	171	9.56E-03
269.1244	151	931	4.56E-02
269.1384	83	1006	4.84E-02
270.9778	103	379	1.95E-02
272.915	129	146	8.61E-03
274.8734	135	40	2.15E-03
274.9323	296	354	1.85E-02
275.1492	244	894	4.40E-02
275.9384	294	987	4.78E-02
275.9912	21	272	1.44E-02
276.0041	292	5	1.53E-04
277.061	82	569	2.91E-02
277.1588	297	660	3.31E-02
277.9828	143	240	1.32E-02
278.8696	134	848	4.19E-02
280.0968	12	65	3.91E-03
281.009	45	295	1.54E-02
281.1747	291	614	3.13E-02
281.9937	95	269	1.43E-02
282.0359	89	944	4.60E-02
283.0075	42	183	1.04E-02
284.0033	282	565	2.89E-02
284.1938	288	24	1.49E-03
284.2301	12	754	3.76E-02
284.8943	295	706	3.55E-02
284.9103	7	896	4.40E-02
286.165	18	250	1.36E-02
287.2411	280	41	2.27E-03
290.0198	291	121	7.09E-03
290.16	293	641	3.23E-02
291.0349	215	154	8.96E-03
291.1204	113	978	4.74E-02
293.9568	143	979	4.76E-02
295.1516	106	636	3.20E-02

295.1901	16	291	1.53E-02
295.2267	24	550	2.82E-02
297.1099	8	597	3.06E-02
297.2213	27	403	2.09E-02
297.9677	96	320	1.69E-02
297.9859	287	156	9.01E-03
298.1147	40	48	2.81E-03
298.2457	22	231	1.26E-02
299.0455	225	467	2.37E-02
300.0243	60	453	2.30E-02
300.2615	90	257	1.39E-02
300.2899	279	122	7.10E-03
301.2526	23	831	4.11E-02
302.1964	236	519	2.69E-02
302.9844	94	634	3.20E-02
303.1803	11	155	9.01E-03
304.2996	90	992	4.79E-02
305.978	143	76	4.69E-03
309.1311	12	814	4.03E-02
309.1311	278	917	4.50E-02
309.1464	225	576	2.94E-02
310.0018	297	273	1.45E-02
310.2093	246	934	4.57E-02
311.1244	80	537	2.76E-02
313.2372	220	667	3.34E-02
317.1786	12	880	4.32E-02
318.191	32	621	3.15E-02
319.0069	94	895	4.40E-02
319.1944	225	676	3.40E-02
320.0635	245	139	8.13E-03
321.0251	112	54	3.14E-03
323.1102	277	441	2.26E-02
323.1842	15	158	9.07E-03
323.258	232	152	8.81E-03
324.1878	12	870	4.30E-02
324.2248	12	637	3.21E-02
324.9688	94	452	2.29E-02
325.1129	234	838	4.15E-02

325.1525	82	265	1.42E-02
325.2009	83	1020	4.92E-02
325.2925	21	825	4.10E-02
326.086	36	439	2.25E-02
326.2957	286	438	2.25E-02
327.089	35	150	8.76E-03
328.082	36	285	1.50E-02
328.1024	35	765	3.80E-02
331.035	284	279	1.48E-02
331.9884	291	268	1.43E-02
332.0805	99	909	4.44E-02
332.2307	35	377	1.95E-02
334.0206	298	237	1.30E-02
335.0977	131	340	1.77E-02
335.1984	132	888	4.37E-02
336.2021	293	497	2.52E-02
337.0171	89	37	2.12E-03
337.1413	88	915	4.49E-02
337.31	229	779	3.84E-02
338.1291	275	946	4.60E-02
338.3421	73	989	4.78E-02
340.1449	24	309	1.63E-02
341.1205	11	642	3.23E-02
341.2752	26	670	3.36E-02
343.0594	96	51	3.03E-03
343.1882	11	34	2.07E-03
343.2259	23	952	4.63E-02
344.9932	103	182	1.03E-02
345.9762	298	865	4.28E-02
347.2179	72	622	3.15E-02
348.1593	56	631	3.18E-02
349.0932	297	1027	4.95E-02
349.1831	30	102	6.28E-03
350.2393	14	85	4.91E-03
351.1	188	161	9.13E-03
351.1569	143	188	1.07E-02
351.1934	9	866	4.29E-02
353.1578	280	423	2.15E-02

353.1728	20	518	2.68E-02
353.1942	286	546	2.81E-02
353.2307	172	422	2.15E-02
353.9763	99	316	1.67E-02
354.1975	10	367	1.91E-02
354.8946	103	434	2.23E-02
358.3679	32	606	3.09E-02
359.3714	32	549	2.82E-02
359.9834	291	198	1.11E-02
360.1808	276	351	1.81E-02
360.9784	95	707	3.55E-02
361.242	10	607	3.11E-02
361.3316	11	671	3.37E-02
363.221	233	993	4.79E-02
363.9127	296	1026	4.95E-02
365.0353	290	280	1.49E-02
365.3202	25	226	1.24E-02
365.9551	96	450	2.28E-02
367.0657	297	356	1.87E-02
367.3359	24	256	1.39E-02
368.1549	219	302	1.59E-02
368.3393	24	324	1.72E-02
370.1344	8	47	2.71E-03
370.1346	235	151	8.76E-03
371.1252	55	113	6.71E-03
371.1682	15	971	4.72E-02
371.2584	25	255	1.39E-02
372.1558	37	387	2.01E-02
373.1451	206	363	1.90E-02
373.9653	143	52	3.04E-03
375.9783	288	603	3.08E-02
376.0693	107	61	3.48E-03
376.9421	101	981	4.76E-02
379.3207	15	339	1.77E-02
379.9954	139	616	3.13E-02
381.2261	34	889	4.37E-02
381.3152	28	877	4.31E-02
382.1338	18	540	2.78E-02
382.1921	9	579	2.96E-02

383.1102	10	132	7.70E-03
383.1832	8	27	1.56E-03
383.2793	12	656	3.28E-02
383.3309	25	2	5.37E-05
384.1138	210	305	1.60E-02
384.2081	10	406	2.09E-02
384.3343	25	1	1.33E-05
384.9667	285	227	1.24E-02
387.9769	288	106	6.45E-03
387.9777	92	769	3.80E-02
388.3059	33	923	4.53E-02
389.3075	28	246	1.35E-02
389.9393	142	419	2.14E-02
390.176	240	286	1.51E-02
392.0882	87	959	4.67E-02
393.2403	7	871	4.30E-02
393.9476	280	943	4.60E-02
395.1313	11	401	2.07E-02
395.3308	25	903	4.42E-02
396.1499	33	567	2.89E-02
396.223	14	900	4.41E-02
397.2203	11	75	4.56E-03
397.2353	207	596	3.06E-02
397.8759	125	162	9.14E-03
399.1053	226	721	3.61E-02
400.3059	28	890	4.38E-02
400.8195	94	664	3.34E-02
401.3423	28	566	2.89E-02
401.8981	101	947	4.60E-02
402.3456	26	514	2.67E-02
403.0922	298	559	2.87E-02
403.2324	21	527	2.72E-02
403.3194	273	409	2.10E-02
403.973	291	323	1.71E-02
405.0298	73	511	2.66E-02
405.1886	264	461	2.36E-02
405.2253	265	919	4.51E-02
406.2074	245	90	5.13E-03
406.6832	49	196	1.10E-02

407.1312	13	945	4.60E-02
407.2046	263	244	1.34E-02
407.2205	284	713	3.59E-02
407.2409	272	857	4.25E-02
407.3671	23	460	2.36E-02
407.9531	288	829	4.11E-02
408.1087	87	578	2.96E-02
408.8901	123	949	4.61E-02
408.9307	95	462	2.36E-02
410.1646	22	108	6.45E-03
410.2688	27	740	3.68E-02
411.0469	96	517	2.68E-02
411.3238	24	217	1.20E-02
413.2664	15	508	2.62E-02
414.07	95	927	4.54E-02
414.2335	221	486	2.49E-02
415.9703	288	454	2.31E-02
416.1401	218	673	3.39E-02
417.1725	215	920	4.52E-02
417.2204	24	417	2.13E-02
417.2483	247	821	4.06E-02
417.2994	254	45	2.57E-03
417.9687	96	595	3.06E-02
419.1682	8	201	1.13E-02
421.1262	207	916	4.50E-02
421.9427	94	594	3.06E-02
421.9637	99	794	3.93E-02
422.0991	83	482	2.47E-02
422.2237	10	298	1.57E-02
422.29	13	832	4.13E-02
422.2974	11	560	2.87E-02
422.8822	102	125	7.25E-03
422.9856	95	675	3.40E-02
423.1028	25	200	1.11E-02
423.1992	162	179	9.94E-03
425.3628	13	243	1.33E-02
425.3779	23	577	2.95E-02
426.1605	13	936	4.57E-02
426.3945	10	694	3.49E-02

427.1726	91	798	3.94E-02
427.2086	36	758	3.76E-02
428.1976	273	177	9.80E-03
428.2343	10	988	4.78E-02
428.2645	251	408	2.10E-02
428.9553	286	31	2.02E-03
429.3005	25	912	4.47E-02
429.9322	144	253	1.37E-02
430.3528	33	366	1.91E-02
432.332	34	855	4.24E-02
432.9184	102	39	2.13E-03
433.2198	270	778	3.83E-02
433.9426	95	167	9.30E-03
434.2387	264	329	1.75E-02
434.275	251	349	1.81E-02
435.1413	223	818	4.04E-02
436.1143	90	23	1.43E-03
437.1181	90	236	1.29E-02
437.3439	27	661	3.32E-02
438.1193	91	735	3.66E-02
438.1606	205	277	1.48E-02
439.2307	10	1012	4.87E-02
440.2342	272	801	3.97E-02
440.2778	214	728	3.63E-02
441.1885	92	120	7.08E-03
441.3573	19	209	1.16E-02
441.3731	25	498	2.52E-02
444.1865	28	355	1.85E-02
444.2084	221	362	1.90E-02
444.9297	102	288	1.52E-02
445.1472	231	430	2.17E-02
445.2945	25	428	2.16E-02
451.4149	10	802	3.97E-02
452.322	271	199	1.11E-02
452.8423	117	261	1.42E-02
453.2828	272	304	1.59E-02
454.2929	51	796	3.94E-02
455.2404	226	95	5.37E-03
455.9148	285	133	7.73E-03

457.3156	29	601	3.07E-02
458.0067	282	126	7.31E-03
458.0964	91	863	4.27E-02
458.2454	10	416	2.12E-02
459.1632	10	879	4.32E-02
460.1025	93	12	4.71E-04
461.2362	281	985	4.77E-02
461.2514	259	114	6.73E-03
461.2735	16	334	1.75E-02
461.3391	187	777	3.83E-02
463.3036	269	752	3.76E-02
465.0678	89	13	5.95E-04
465.2465	267	487	2.49E-02
465.3938	241	1030	4.97E-02
465.8658	124	289	1.52E-02
467.3361	20	378	1.95E-02
468.1716	219	364	1.90E-02
468.3902	275	699	3.50E-02
468.4048	31	245	1.34E-02
469.1261	91	868	4.30E-02
470.3131	186	677	3.41E-02
471.272	17	437	2.25E-02
472.3634	245	544	2.81E-02
473.214	12	747	3.72E-02
473.236	279	556	2.85E-02
474.0707	90	145	8.46E-03
474.2187	210	663	3.33E-02
474.2697	22	4	9.78E-05
476.0682	91	449	2.28E-02
476.2982	22	53	3.04E-03
476.3216	242	1031	4.98E-02
477.3559	261	572	2.92E-02
479.1892	13	374	1.94E-02
479.9899	277	8	2.55E-04
480.2655	273	905	4.42E-02
480.3088	50	314	1.66E-02
481.2565	228	652	3.26E-02
481.3514	23	930	4.55E-02
482.2236	218	84	4.90E-03

482.313	39	278	1.48E-02
483.2472	150	739	3.67E-02
484.2817	44	306	1.61E-02
484.7823	181	249	1.36E-02
484.7827	93	628	3.17E-02
485.0817	56	722	3.61E-02
485.3256	25	924	4.53E-02
485.9563	96	1010	4.86E-02
489.2311	10	175	9.75E-03
489.9304	96	956	4.66E-02
489.9513	99	887	4.36E-02
491.1082	93	543	2.80E-02
492.2445	225	975	4.73E-02
492.2805	22	191	1.08E-02
493.293	32	420	2.14E-02
494.2969	237	22	1.33E-03
494.8101	135	506	2.60E-02
496.3856	168	353	1.82E-02
497.3221	170	166	9.24E-03
497.3644	176	815	4.03E-02
497.9933	97	290	1.52E-02
498.1845	207	558	2.87E-02
498.9536	205	336	1.76E-02
499.3768	25	66	3.96E-03
500.3504	170	192	1.09E-02
501.2308	273	79	4.80E-03
501.3046	267	424	2.15E-02
501.9303	95	463	2.36E-02
502.214	289	571	2.92E-02
502.6371	50	72	4.40E-03
503.0254	90	6	1.92E-04
503.3318	147	457	2.35E-02
504.333	148	347	1.80E-02
504.3421	160	358	1.88E-02
505.424	256	19	1.19E-03
506.385	23	819	4.04E-02
506.6724	50	768	3.80E-02
507.2591	34	1033	4.99E-02
508.2617	182	181	1.02E-02

509.3088	270	1013	4.87E-02
509.3336	148	573	2.92E-02
510.32	212	991	4.79E-02
511.0192	294	891	4.38E-02
511.215	272	840	4.16E-02
511.3035	33	983	4.76E-02
513.2311	274	717	3.59E-02
515.2467	274	176	9.77E-03
515.3729	23	233	1.27E-02
516.3035	149	60	3.46E-03
516.3104	152	287	1.52E-02
516.8795	112	864	4.28E-02
517.2042	237	813	4.03E-02
517.9015	95	186	1.06E-02
518.1131	36	317	1.68E-02
518.2455	213	808	4.01E-02
518.3458	152	804	3.99E-02
518.392	23	212	1.18E-02
519.2363	196	872	4.31E-02
519.2569	233	216	1.19E-02
519.2702	47	678	3.42E-02
519.3048	151	687	3.46E-02
520.3309	146	348	1.81E-02
521.2728	11	307	1.63E-02
521.2965	21	968	4.71E-02
521.3091	11	384	2.00E-02
522.2552	225	972	4.72E-02
522.2916	250	276	1.47E-02
522.379	164	684	3.44E-02
522.386	168	790	3.92E-02
522.528	58	718	3.60E-02
522.8183	123	624	3.16E-02
523.1537	63	426	2.16E-02
524.2547	12	655	3.28E-02
524.2704	220	803	3.98E-02
527.1895	229	15	9.44E-04
528.9441	183	723	3.61E-02
529.2253	272	190	1.08E-02
529.2257	12	404	2.09E-02

530.26	21	292	1.54E-02
532.2631	205	103	6.30E-03
532.2774	157	321	1.70E-02
532.3124	29	251	1.37E-02
533.236	150	885	4.35E-02
533.4187	267	219	1.22E-02
533.4541	259	25	1.53E-03
534.0083	91	493	2.50E-02
534.3173	161	435	2.24E-02
534.3417	150	807	4.00E-02
534.8847	199	281	1.49E-02
534.9559	104	562	2.88E-02
535.0092	90	851	4.22E-02
535.2872	268	429	2.16E-02
535.3228	266	1003	4.82E-02
535.8995	54	644	3.24E-02
536.3065	14	647	3.25E-02
536.7921	138	262	1.42E-02
537.3766	11	346	1.80E-02
538.2978	163	471	2.39E-02
538.4499	23	1029	4.97E-02
538.7922	135	475	2.42E-02
539.1892	225	296	1.56E-02
540.3031	262	242	1.33E-02
540.5179	47	222	1.22E-02
540.7997	126	32	2.04E-03
541.074	55	1014	4.88E-02
542.2816	201	229	1.25E-02
542.4407	13	604	3.08E-02
543.0396	33	701	3.51E-02
543.4031	22	83	4.88E-03
544.3374	150	619	3.15E-02
544.3629	149	632	3.18E-02
545.037	34	413	2.12E-02
545.1258	35	1016	4.91E-02
545.3377	142	178	9.87E-03
548.3085	23	451	2.28E-02
549.3097	152	415	2.12E-02
552.2655	231	922	4.52E-02

552.2869	195	691	3.48E-02
552.3019	263	77	4.77E-03
552.3354	227	469	2.38E-02
553.2989	272	856	4.25E-02
554.4421	23	480	2.45E-02
555.1735	91	657	3.28E-02
556.2224	45	111	6.54E-03
557.198	214	850	4.21E-02
557.2365	233	70	4.20E-03
557.2722	90	940	4.59E-02
557.9388	99	898	4.40E-02
557.9697	97	755	3.76E-02
558.3637	249	749	3.75E-02
559.9419	101	11	3.22E-04
560.3061	34	303	1.59E-02
563.3087	152	841	4.16E-02
564.3034	38	293	1.54E-02
564.4804	47	970	4.72E-02
565.4434	21	455	2.32E-02
565.9802	99	648	3.25E-02
566.8788	101	1001	4.81E-02
566.8997	103	709	3.56E-02
567.3511	10	170	9.48E-03
568.0388	57	328	1.75E-02
569.085	56	432	2.20E-02
569.913	94	381	1.99E-02
570.9257	92	665	3.34E-02
572.2957	157	221	1.22E-02
573.3425	30	94	5.30E-03
574.6159	50	300	1.58E-02
574.8361	112	933	4.57E-02
575.9917	90	948	4.61E-02
576.283	154	235	1.29E-02
576.2831	248	771	3.81E-02
576.3053	212	705	3.54E-02
577.3355	13	165	9.23E-03
577.3728	27	232	1.27E-02
579.2059	31	522	2.70E-02
580.2967	41	849	4.20E-02

580.9148	93	1000	4.81E-02
581.9627	105	220	1.22E-02
583.3459	10	583	3.00E-02
585.8889	94	91	5.13E-03
588.2702	152	817	4.04E-02
588.3104	146	345	1.80E-02
588.3747	31	370	1.92E-02
588.4445	21	828	4.11E-02
588.9063	98	780	3.84E-02
589.3185	141	311	1.65E-02
590.3358	31	672	3.38E-02
593.2936	264	147	8.67E-03
593.3421	139	440	2.26E-02
594.1588	282	712	3.58E-02
594.2514	207	986	4.77E-02
594.8516	54	724	3.62E-02
595.1383	103	942	4.60E-02
595.1481	135	738	3.66E-02
595.3827	25	731	3.64E-02
596.9167	96	135	7.89E-03
597.2686	153	187	1.06E-02
598.8285	122	958	4.66E-02
599.3198	205	373	1.93E-02
600.266	163	228	1.24E-02
600.3245	150	56	3.24E-03
601.2838	10	653	3.28E-02
601.3005	212	666	3.34E-02
601.321	275	520	2.69E-02
601.8618	94	260	1.42E-02
603.2952	37	525	2.71E-02
605.2404	31	907	4.43E-02
605.2844	152	489	2.50E-02
609.3108	149	593	3.05E-02
610.1845	288	481	2.46E-02
610.5408	25	835	4.14E-02
612.3171	142	284	1.50E-02
612.6044	50	202	1.13E-02
613.3947	30	528	2.72E-02
613.8858	95	357	1.87E-02

614.331	139	800	3.96E-02
614.4481	23	425	2.16E-02
614.4516	140	476	2.43E-02
616.2952	150	445	2.27E-02
616.4459	46	625	3.16E-02
617.2902	225	1004	4.83E-02
617.2963	232	772	3.82E-02
617.4939	31	842	4.16E-02
618.4441	45	93	5.22E-03
619.2477	256	729	3.63E-02
619.2718	226	523	2.70E-02
620.2561	173	605	3.08E-02
620.7565	94	612	3.12E-02
622.6332	50	957	4.66E-02
625.2573	209	700	3.50E-02
625.9247	99	869	4.30E-02
626.8447	103	96	5.70E-03
627.2957	39	555	2.85E-02
627.9281	101	100	6.05E-03
628.8785	138	215	1.18E-02
629.3539	13	153	8.91E-03
629.5529	26	950	4.62E-02
630.2972	156	86	4.94E-03
631.1075	128	976	4.74E-02
635.306	134	564	2.88E-02
635.3145	140	1022	4.93E-02
637.32	270	218	1.21E-02
637.3232	10	635	3.20E-02
637.8998	95	407	2.10E-02
638.2742	264	679	3.43E-02
639.974	88	789	3.92E-02
641.9294	97	510	2.65E-02
643.2759	229	785	3.90E-02
643.3264	15	795	3.94E-02
647.2919	146	853	4.24E-02
648.5098	26	472	2.40E-02
648.9509	103	516	2.67E-02
649.4372	144	330	1.75E-02
652.2824	145	395	2.04E-02

653.8532	110	584	3.00E-02
653.8794	93	78	4.80E-03
654.2924	140	999	4.81E-02
654.2979	142	380	1.98E-02
657.3237	203	325	1.73E-02
658.3109	126	776	3.83E-02
662.863	131	685	3.44E-02
668.2546	143	547	2.81E-02
672.2621	142	932	4.56E-02
672.269	150	375	1.94E-02
672.2785	139	1009	4.85E-02
672.8611	98	448	2.27E-02
675.0729	220	680	3.43E-02
677.5503	82	410	2.11E-02
679.0922	101	20	1.27E-03
681.3176	135	535	2.76E-02
682.3343	132	539	2.77E-02
682.5462	51	939	4.59E-02
683.5404	23	860	4.26E-02
684.2868	132	883	4.34E-02
685.8258	93	689	3.47E-02
685.9547	87	398	2.06E-02
686.2343	154	697	3.50E-02
687.2293	28	301	1.58E-02
687.2436	157	554	2.84E-02
688.2482	144	372	1.93E-02
689.7967	90	874	4.31E-02
691.9849	91	602	3.08E-02
693.9149	99	858	4.26E-02
694.363	33	143	8.41E-03
694.3739	40	515	2.67E-02
700.5617	48	331	1.75E-02
700.7774	129	270	1.44E-02
701.8005	92	774	3.82E-02
702.2998	132	477	2.45E-02
702.9596	102	82	4.87E-03
703.5376	39	466	2.37E-02
704.307	128	118	6.98E-03
704.3196	124	658	3.29E-02

710.1115	55	786	3.90E-02
712.2621	152	781	3.87E-02
714.7563	132	397	2.06E-02
716.9354	104	172	9.56E-03
718.269	140	908	4.43E-02
720.4904	50	117	6.96E-03
720.9796	54	686	3.45E-02
722.2821	138	973	4.72E-02
722.9768	54	513	2.66E-02
725.544	111	695	3.50E-02
726.5502	105	203	1.14E-02
733.9028	130	447	2.27E-02
734.8991	128	826	4.10E-02
738.2549	144	639	3.22E-02
738.6117	25	504	2.57E-02
739.2572	146	698	3.50E-02
746.5397	49	720	3.61E-02
748.2913	133	852	4.23E-02
749.1656	72	49	2.87E-03
751.7877	121	433	2.22E-02
752.1635	82	28	1.72E-03
753.5554	92	962	4.69E-02
754.2324	153	716	3.59E-02
755.2338	151	982	4.76E-02
756.2292	143	726	3.63E-02
756.5809	72	1023	4.93E-02
757.9036	95	322	1.70E-02
761.902	99	881	4.32E-02
763.1781	103	494	2.51E-02
764.8529	138	326	1.73E-02
766.2059	64	617	3.14E-02
768.8058	120	26	1.54E-03
772.8979	124	57	3.26E-03
775.9257	93	715	3.59E-02
779.4925	157	688	3.47E-02
780.2362	137	313	1.65E-02
782.527	44	682	3.44E-02
782.8516	112	390	2.02E-02
784.7214	132	168	9.40E-03

785.0933	58	50	2.99E-03
785.359	95	38	2.13E-03
785.6397	73	744	3.71E-02
785.9175	117	674	3.39E-02
793.8533	99	1021	4.92E-02
794.7682	122	784	3.89E-02
796.224	144	148	8.70E-03
796.5405	48	787	3.90E-02
797.791	98	294	1.54E-02
799.5751	49	757	3.76E-02
800.7748	48	626	3.17E-02
802.5433	94	411	2.11E-02
805.8066	116	44	2.56E-03
807.2451	143	875	4.31E-02
808.2496	136	921	4.52E-02
808.4409	50	793	3.93E-02
808.45	26	733	3.64E-02
808.7505	126	55	3.18E-03
810.4373	50	104	6.31E-03
810.5174	132	668	3.35E-02
810.7034	100	732	3.64E-02
810.79	119	483	2.47E-02
811.3528	106	551	2.83E-02
813.5202	89	806	4.00E-02
813.5265	47	809	4.01E-02
813.5398	92	532	2.75E-02
818.3147	124	548	2.81E-02
818.6194	45	651	3.26E-02
819.7758	121	42	2.37E-03
820.8652	109	46	2.60E-03
821.086	52	159	9.08E-03
822.2111	145	756	3.76E-02
824.0397	51	1024	4.94E-02
824.2143	140	128	7.46E-03
824.5701	89	141	8.34E-03
824.9198	108	157	9.05E-03
826.7609	127	762	3.79E-02
829.0583	53	488	2.49E-02
833.4941	110	500	2.54E-02

834.6515	47	542	2.79E-02
835.5326	97	791	3.93E-02
836.7522	128	344	1.79E-02
837.2955	119	464	2.37E-02
837.4246	103	80	4.84E-03
837.89	96	692	3.49E-02
837.9291	103	645	3.25E-02
838.2612	119	1025	4.94E-02
840.8856	122	16	1.07E-03
842.3504	100	1015	4.91E-02
844.7833	112	867	4.29E-02
845.5549	56	574	2.93E-02
846.5397	89	997	4.80E-02
848.771	218	64	3.90E-03
851.6354	93	443	2.27E-02
852.7204	131	479	2.45E-02
854.3836	50	623	3.15E-02
854.7961	103	87	4.97E-03
856.4222	50	995	4.80E-02
859.3652	22	105	6.45E-03
860.3869	155	371	1.92E-02
861.7537	126	938	4.58E-02
862.1475	55	830	4.11E-02
862.4054	152	748	3.74E-02
863.1672	56	393	2.04E-02
865.5048	94	174	9.75E-03
869.7352	131	318	1.69E-02
871.2886	118	89	5.05E-03
871.6108	44	507	2.60E-02
872.5127	52	955	4.66E-02
872.6312	79	711	3.58E-02
874.9142	54	264	1.42E-02
875.5499	53	954	4.64E-02
875.6485	66	491	2.50E-02
876.6511	70	650	3.26E-02
876.7384	127	969	4.71E-02
877.6468	73	1002	4.81E-02
877.6632	72	502	2.56E-02
878.1844	99	1028	4.95E-02

878.6629	72	436	2.25E-02
878.7738	116	773	3.82E-02
878.8517	108	834	4.14E-02
880.9883	51	649	3.26E-02
887.6615	42	1007	4.84E-02
887.8278	107	123	7.14E-03
891.2069	139	783	3.89E-02
892.2075	133	640	3.23E-02
892.3815	101	112	6.56E-03
892.7152	137	630	3.18E-02
894.7477	124	585	3.00E-02
900.3578	50	342	1.77E-02
903.6822	70	505	2.58E-02
903.7406	125	405	2.09E-02
904.4082	50	360	1.89E-02
904.6824	71	173	9.58E-03
905.3506	105	69	4.19E-03
906.192	141	910	4.46E-02
910.667	42	873	4.31E-02
911.8139	118	737	3.66E-02
912.3328	50	967	4.71E-02
912.7654	115	984	4.76E-02
913.2691	120	904	4.42E-02
913.7616	117	1011	4.86E-02
916.3359	50	839	4.15E-02
918.3281	101	207	1.15E-02
918.4238	50	312	1.65E-02
919.71	129	456	2.34E-02
920.3693	47	669	3.35E-02
920.714	131	137	7.97E-03
922.4899	47	990	4.78E-02
928.7368	122	36	2.10E-03
928.7374	121	761	3.78E-02
936.7262	125	745	3.71E-02
936.8603	124	966	4.71E-02
938.5636	92	368	1.92E-02
944.7172	126	693	3.49E-02
947.2632	119	529	2.73E-02
948.2809	47	98	5.96E-03

952.255	116	274	1.46E-02
952.8347	130	10	3.13E-04
954.7638	118	382	1.99E-02
957.3542	103	81	4.86E-03
958.4285	51	845	4.17E-02
962.2304	47	941	4.60E-02
963.8264	112	727	3.63E-02
970.7337	121	431	2.19E-02
971.7207	124	710	3.57E-02
972.1195	53	193	1.10E-02
972.7255	124	197	1.11E-02
976.2813	50	906	4.43E-02
978.3739	50	743	3.70E-02
982.3226	50	271	1.44E-02
985.1379	47	1017	4.91E-02
986.2258	133	297	1.56E-02
988.6992	129	189	1.07E-02
988.7452	119	846	4.19E-02
989.7347	119	753	3.76E-02
993.6782	166	897	4.40E-02
1001.0967	59	414	2.12E-02
1004.8392	96	74	4.55E-03
1005.8142	107	1032	4.99E-02
1008.0825	47	534	2.76E-02
1008.8518	108	598	3.06E-02
1013.6981	128	878	4.32E-02
1015.2461	116	130	7.50E-03
1016.3757	51	746	3.71E-02
1017.3851	50	884	4.35E-02
1018.2785	50	750	3.75E-02
1023.7552	121	570	2.92E-02
1024.3008	51	799	3.95E-02
1026.3098	51	633	3.19E-02
1028.2985	50	149	8.71E-03
1032.3327	50	14	6.77E-04
1034.6106	94	994	4.79E-02
1037.6582	153	459	2.35E-02
1049.2552	114	396	2.05E-02
1054.6994	125	418	2.13E-02

1062.5155	54	886	4.35E-02
1071.3539	35	736	3.66E-02
1074.6366	67	43	2.52E-03
1076.6248	66	33	2.04E-03
1080.2176	50	961	4.69E-02
1084.2118	50	524	2.71E-02
1084.2692	50	444	2.27E-02
1086.6397	134	1008	4.85E-02
1096.5807	63	638	3.21E-02
1097.6195	65	399	2.07E-02
1099.6328	67	343	1.78E-02
1099.7991	98	234	1.29E-02
1112.5562	86	35	2.10E-03
1112.5811	83	538	2.76E-02
1114.5856	85	115	6.74E-03
1119.5759	64	30	1.98E-03
1125.6379	68	211	1.17E-02
1135.7396	106	512	2.66E-02
1141.2764	92	282	1.50E-02
1144.7714	100	620	3.15E-02
1146.2076	50	67	3.98E-03
1146.6189	67	18	1.19E-03
1168.8083	102	618	3.14E-02
1169.8227	99	926	4.54E-02
1174.8483	52	662	3.33E-02
1185.7196	113	359	1.88E-02
1185.8567	51	659	3.30E-02
1186.7561	101	759	3.76E-02
1189.6803	75	7	2.44E-04
1211.8234	23	913	4.48E-02
1226.8106	23	71	4.24E-03

Raw p values < 0.05
ESI=electrospray ionization
HILIC= hydrophilic interaction liquid chromatography
MWAS= metabolome-wide association study
TPP = thiamine pyrophosphate

Supplemental table 3. Significant metabolic features linked to TPP concentrations from the C18 negative ESI column: lowest versus highest tertile

m/z	Time (sec)	Mean Highest tertile (normalized)	Mean Lowest tertile (normalized)	Differential expression rank	P value
85.9989	17	17.21	17.44	236	1.73E-02
89.673	16	17.17	17.38	280	2.01E-02
93.0206	299	15.59	16.01	529	3.72E-02
93.0457	288	17.75	17.56	716	4.98E-02
97.3239	239	18.31	17.98	38	2.58E-03
100.9645	146	16.12	17.04	138	9.81E-03
103.9202	15	17.38	17.62	588	4.06E-02
111.0199	296	19.87	19.34	185	1.30E-02
112.0736	261	17.44	16.55	410	2.84E-02
112.9372	16	19.57	20.17	512	3.59E-02
113.0141	26	16.08	16.52	125	8.71E-03
118.0411	23	19.43	18.94	424	2.98E-02
120.0123	24	15.28	15.93	157	1.10E-02
126.0308	21	17.05	17.68	57	4.31E-03
127.0398	164	19.79	19.62	264	1.94E-02
131.0461	22	18.56	18.71	505	3.55E-02
132.8678	18	15.98	16.67	131	9.18E-03
132.9463	165	16.94	17.38	656	4.54E-02
140.0203	17	16.51	16.67	598	4.15E-02
143.0825	16	18.89	19.2	233	1.71E-02
144.0152	16	15.6	16.04	216	1.54E-02
145.0281	294	16.84	16.65	387	2.70E-02
145.0379	15	18.12	18.37	510	3.58E-02
145.0981	15	16.5	16.86	443	3.08E-02
147.0586	299	15.6	14.89	671	4.66E-02
149.0244	296	15.44	15.92	195	1.37E-02
149.0455	18	19.93	20.33	695	4.80E-02
151.0705	281	18.92	18.62	184	1.30E-02
153.0668	286	17.53	17.05	116	8.30E-03
154.0186	16	15.61	15.78	154	1.08E-02
154.0507	156	17.67	17.18	119	8.46E-03
154.062	284	18.6	18.26	649	4.51E-02

155.9978	16	15.69	15.97	194	1.35E-02
156.0226	16	22.19	22.42	450	3.15E-02
156.0396	290	17.24	17.07	676	4.68E-02
156.5233	16	18.88	19.11	456	3.20E-02
157.0156	295	15.76	15.51	442	3.07E-02
157.0214	16	19.15	19.44	297	2.10E-02
157.0248	15	16.81	16.99	426	2.99E-02
157.0869	22	20.06	21	115	8.27E-03
157.0982	16	19.14	19.3	668	4.64E-02
157.5231	15	15.86	16.2	129	9.11E-03
158.0609	93	19.68	19.47	415	2.90E-02
159.0662	20	19.39	19.54	409	2.84E-02
159.1139	16	17.82	18.16	447	3.14E-02
160.0615	18	18.8	19.06	277	1.99E-02
160.7737	17	14.29	14.8	437	3.05E-02
164.8954	33	17.41	17.7	163	1.13E-02
165.0094	15	18.35	18.52	590	4.07E-02
165.0135	16	19.87	20.12	403	2.80E-02
166.0223	31	17.66	18.07	99	7.31E-03
170.1267	130	14.53	16	544	3.83E-02
172.0102	17	17.19	17.36	685	4.74E-02
172.0252	16	16.66	17.15	341	2.38E-02
172.088	282	18.57	18.17	266	1.95E-02
174.0303	24	17.47	17.04	488	3.42E-02
174.0309	283	19.37	19.2	718	4.99E-02
176.002	15	16.76	17.36	418	2.91E-02
178.0509	21	20.79	21.8	282	2.02E-02
178.0734	284	19.07	18.88	592	4.10E-02
180.0596	22	18.71	18.98	665	4.61E-02
183.1753	171	17.89	17.53	473	3.30E-02
186.0258	17	16.04	16.27	402	2.80E-02
187.0724	15	20	20.34	314	2.19E-02
188.0771	295	16.62	16.38	386	2.70E-02
189.088	18	17.15	17.73	52	3.65E-03
190.0355	16	21.13	21.62	372	2.61E-02
190.0544	19	17.02	17.49	434	3.02E-02
191.0388	16	16.81	17.23	172	1.22E-02
192.0527	290	17.95	17.84	226	1.66E-02
192.089	282	19.16	18.81	526	3.71E-02

192.9813	18	15.91	16.64	645	4.44E-02
195.0525	291	17.02	16.06	109	7.79E-03
196.9034	268	16.91	17.47	508	3.57E-02
197.0223	18	16.51	16.95	697	4.81E-02
197.0663	286	18.86	18.66	392	2.73E-02
197.1183	25	18.05	18.3	388	2.72E-02
198.0258	18	18.56	18.81	344	2.38E-02
200.0416	17	16.69	16.88	534	3.76E-02
200.0467	293	16.97	16.68	555	3.87E-02
201.035	20	17.41	16.9	607	4.22E-02
201.0882	16	20.2	20.73	93	6.78E-03
202.0816	287	17.83	17.27	71	5.27E-03
203.1039	17	17.92	18.39	439	3.05E-02
204.9786	60	17.21	17.7	571	3.94E-02
205.0522	294	16.54	16.03	120	8.47E-03
206.0573	287	18.12	17.89	146	1.03E-02
206.1049	288	18.03	17.71	337	2.34E-02
210.9317	32	20.28	19.64	633	4.39E-02
211.1341	217	18.99	19.5	623	4.34E-02
213.002	25	17.96	17.4	145	1.02E-02
213.0615	296	16.18	15.77	371	2.58E-02
214.0828	122	19.55	19.87	445	3.11E-02
216.0193	16	18.02	18.21	659	4.56E-02
216.0647	16	15.32	16.11	429	3.00E-02
216.0709	16	19.45	19.82	683	4.73E-02
218.0403	20	17.54	17.18	579	4.00E-02
218.0674	17	16.8	17.05	225	1.64E-02
220.1471	18	16.19	15.87	640	4.43E-02
221.0247	17	15.73	16.22	56	4.17E-03
222.1348	173	18.29	18.78	161	1.12E-02
227.0527	17	17.33	17.53	533	3.75E-02
227.1291	214	20.08	20.37	548	3.84E-02
229.0598	149	16.21	17.69	181	1.28E-02
229.0845	226	21.37	20.65	707	4.91E-02
230.0889	30	15.27	15.81	572	3.96E-02
231.0525	293	17.07	16.82	378	2.63E-02
231.099	18	16.54	17.14	228	1.67E-02
234.1	280	19.18	18.91	625	4.36E-02
236.0945	235	18.94	17.91	48	3.44E-03

237.02	16	18.64	18.84	653	4.54E-02
237.1137	294	15.01	15.97	118	8.41E-03
241.0687	18	20.46	20.67	687	4.75E-02
241.9998	25	16.1	16.51	45	3.01E-03
242.069	17	16.95	17.24	220	1.57E-02
243.0072	25	16.75	17.12	582	4.01E-02
244.0026	15	18.9	19.16	597	4.15E-02
244.2001	122	16.73	17.21	394	2.74E-02
245.0785	18	19.58	19.83	73	5.33E-03
245.1582	263	18.93	18.38	211	1.51E-02
247.0942	16	20.47	20.83	328	2.28E-02
248.0973	17	16.88	17.14	285	2.03E-02
249.9641	50	16.85	17.22	190	1.33E-02
250.0934	292	17.18	16.94	702	4.87E-02
251.053	17	18.03	18.27	616	4.32E-02
252.1088	284	18.61	18.26	299	2.11E-02
253.0319	20	18.58	18.74	690	4.76E-02
254.0883	294	16.7	16.38	617	4.33E-02
254.1246	291	17.05	16.57	231	1.70E-02
254.9614	263	18.95	18.46	366	2.53E-02
255.0824	237	16.3	16.81	704	4.88E-02
255.1424	236	15.91	16.71	460	3.23E-02
255.9066	261	17.75	17.25	102	7.40E-03
255.9715	170	16.41	17.3	237	1.77E-02
256.0157	17	18.67	18.9	615	4.32E-02
256.9111	144	17.12	17.49	646	4.46E-02
257.0365	19	18.46	18.9	188	1.32E-02
259.0038	15	15.94	16.46	637	4.42E-02
260.1772	117	17.26	16.79	322	2.22E-02
261.2228	214	20.35	20.92	221	1.58E-02
265.0674	105	18.28	17.89	501	3.50E-02
270.8993	34	15.23	15.93	422	2.94E-02
272.0288	16	16.17	16.38	345	2.40E-02
272.0555	19	18.4	18.65	626	4.36E-02
272.0658	15	17.91	18.23	164	1.13E-02
273.0924	21	13.93	16.15	373	2.61E-02
275.0904	296	15.94	16.16	569	3.93E-02
276.9706	34	15.84	16.49	127	9.04E-03
279.0635	214	17.52	18.1	77	5.81E-03

279.3865	217	19.13	19.63	490	3.44E-02
280.1159	295	16.78	16.51	176	1.25E-02
281.2493	241	32.51	32.32	258	1.87E-02
283.0434	16	16.6	16.88	192	1.34E-02
285.0587	17	18.68	19.01	288	2.05E-02
286.9933	24	18.31	18.68	96	7.13E-03
287.2235	25	16.51	17.4	351	2.45E-02
289.0631	295	17.04	17.37	63	4.49E-03
289.0634	20	18.61	18.87	368	2.55E-02
291.1592	125	17.57	18.2	186	1.31E-02
292.0969	16	16.37	16.63	383	2.67E-02
292.2003	162	18.18	18.38	566	3.91E-02
292.9464	263	18.66	18.26	642	4.43E-02
293.0884	15	23.01	23.21	367	2.54E-02
294.0918	17	19.56	19.71	202	1.44E-02
294.216	217	19.42	19.89	585	4.05E-02
295.1154	295	15.92	15.44	629	4.38E-02
295.2284	217	23.44	23.84	660	4.57E-02
296.0441	18	16.71	17.09	113	8.19E-03
296.2318	217	20.66	21.27	50	3.61E-03
296.9192	163	18.67	19.15	330	2.31E-02
297.041	18	17.43	17.42	648	4.49E-02
297.1492	122	18.23	19.2	380	2.64E-02
305.0631	18	18.24	18.47	715	4.98E-02
305.1165	293	15.71	16.24	61	4.40E-03
306.0134	24	15.94	15.41	253	1.85E-02
308.0312	93	17.61	16.99	375	2.62E-02
308.9856	175	16.34	14.01	310	2.16E-02
309.1743	170	17.36	17.18	713	4.96E-02
310.1526	134	11.97	13.69	669	4.65E-02
310.2393	218	18.51	19.13	112	7.96E-03
311.0384	16	17.2	17.45	155	1.09E-02
311.0504	25	16.12	16.68	263	1.94E-02
311.2416	185	18.4	18.13	509	3.57E-02
312.2267	218	19.29	20.17	40	2.77E-03
312.9591	44	17.57	17.78	201	1.44E-02
313.0659	21	23.31	23.66	586	4.06E-02
313.2394	217	20.83	19.92	147	1.03E-02

314.0693	21	20.28	20.55	311	2.17E-02
314.1192	20	12.11	13.88	663	4.59E-02
314.1688	133	16.5	16.75	219	1.56E-02
315.07	27	16.36	16.65	281	2.01E-02
315.1113	289	18.03	17.72	560	3.89E-02
315.2369	259	18.24	17.68	212	1.52E-02
316.0544	17	20.2	20.43	708	4.91E-02
317.0554	17	17	17.32	166	1.16E-02
319.0345	280	17.12	16.4	338	2.36E-02
319.1896	166	16.98	17.8	481	3.38E-02
320.0339	134	18.66	18.46	175	1.23E-02
321.1224	281	18.94	18.61	476	3.33E-02
322.0412	16	16.86	17.17	603	4.17E-02
323.8943	45	15.82	16.63	18	1.22E-03
323.9644	23	17.55	17.79	609	4.24E-02
325.2391	214	22.37	22.8	170	1.19E-02
325.2755	207	20.78	20.39	557	3.88E-02
326.2426	217	20.15	20.52	514	3.61E-02
330.0288	16	18.86	19.11	303	2.13E-02
330.1647	166	16.61	17.39	32	2.05E-03
331.9958	28	19.7	19.94	42	2.95E-03
332.0041	118	18.72	18.09	705	4.89E-02
332.0387	16	17.98	18.13	636	4.42E-02
332.9481	21	15.9	15.06	606	4.21E-02
335.0483	22	23.3	23.67	462	3.23E-02
335.1903	139	17.02	17.41	318	2.21E-02
336.9301	65	18.77	19	465	3.25E-02
339.2356	234	21.96	22.99	41	2.86E-03
339.255	246	18.81	19.22	229	1.67E-02
340.2378	226	19.84	20.89	160	1.12E-02
341.0028	298	15.22	15.8	309	2.16E-02
341.234	272	18.01	18.66	53	3.78E-03
346.0114	24	18.4	18.61	417	2.91E-02
346.8734	134	16.92	15.83	26	1.67E-03
346.9965	16	14.37	16.04	441	3.06E-02
347.1498	296	16.36	15.84	252	1.85E-02
348.1699	296	15.94	16.39	599	4.15E-02
349.1791	168	17.3	17.96	472	3.30E-02

351.1679	274	18.23	17.74	655	4.54E-02
355.2133	268	17.99	17.67	494	3.46E-02
357.1564	165	19.34	19.05	376	2.62E-02
358.2138	160	16.81	16.58	613	4.27E-02
359.0348	28	16.82	17.3	342	2.38E-02
360.0273	27	15.04	15.99	504	3.54E-02
360.9437	56	16.7	17.03	39	2.75E-03
360.9902	137	22.95	22.54	643	4.43E-02
363.1958	217	23.36	23.74	619	4.33E-02
365.1821	22	17.11	17.93	444	3.10E-02
366.0395	133	17.52	17.34	431	3.01E-02
367.1281	155	16.02	16.24	453	3.17E-02
367.2444	233	18.89	18.72	531	3.74E-02
369.1237	154	16.86	16.57	312	2.17E-02
372.2481	164	17.34	17.68	605	4.19E-02
372.9528	45	15.66	15.98	513	3.59E-02
375.0368	18	16.04	16.41	527	3.71E-02
375.2215	214	25.65	26.04	255	1.86E-02
376.1648	295	15.6	16.13	587	4.06E-02
376.2252	214	23.17	23.55	459	3.22E-02
378.9226	162	20.96	21.46	353	2.46E-02
380.8251	186	17.33	16.78	354	2.46E-02
381.214	208	17.42	17.13	294	2.09E-02
382.1812	152	16.77	17.13	85	6.30E-03
382.2043	157	16.97	16.21	104	7.45E-03
382.2436	168	17.5	18.04	379	2.64E-02
382.9162	157	18.65	18.32	650	4.51E-02
387.2028	267	18.83	18.39	641	4.43E-02
389.1817	265	17.12	17.51	717	4.99E-02
390.2686	149	18.57	17.78	33	2.18E-03
390.3138	192	17.4	17.96	500	3.49E-02
390.8951	149	19.73	19.96	634	4.39E-02
391.2356	186	20.43	19.45	9	7.33E-04
392.3065	264	18.73	19.1	240	1.78E-02
392.8523	192	17.49	17.97	694	4.80E-02
394.8408	181	17.37	16.83	385	2.69E-02
394.9888	58	17.36	16.47	22	1.30E-03
396.2582	176	17.13	17.82	69	5.03E-03

396.9318	183	17.77	17.22	106	7.59E-03
396.9724	122	21.95	21.39	489	3.44E-02
397.2371	219	19.14	18.32	218	1.55E-02
398.2378	159	17.46	16.79	12	8.51E-04
398.3366	190	17.35	16.86	359	2.48E-02
400.8894	142	15.96	16.41	532	3.75E-02
403.0354	31	16.48	17.15	11	8.19E-04
403.2162	146	16.81	17.31	28	1.81E-03
403.2463	142	15.7	16.7	168	1.18E-02
405.0293	15	17.87	18.76	538	3.79E-02
405.2296	31	16.6	15.38	672	4.66E-02
406.2177	146	17.01	16.35	348	2.41E-02
406.9102	156	17.2	16.67	251	1.84E-02
408.8765	174	17.58	18.03	295	2.09E-02
411.17	167	18.11	18.27	391	2.73E-02
411.2167	136	16.12	17.11	438	3.05E-02
411.9835	29	17.7	18.03	530	3.72E-02
413.0179	23	18.94	19.24	356	2.47E-02
414.8534	37	15.07	15.72	463	3.25E-02
414.9063	47	15.08	15.95	15	1.12E-03
415.2088	217	18.27	17.48	124	8.71E-03
417.0841	17	18.87	19.26	662	4.58E-02
419.1928	266	18.12	17.75	286	2.04E-02
423.3491	228	18.28	17.86	135	9.47E-03
424.0261	36	15.06	15.5	198	1.39E-02
424.2445	128	15.02	16.27	624	4.35E-02
425.1184	22	16.97	15.93	401	2.80E-02
429.3227	162	17.24	16.78	110	7.90E-03
430.2174	156	18.36	17.96	446	3.11E-02
430.2639	176	17.69	18.1	686	4.75E-02
431.1921	273	17.1	16.64	105	7.56E-03
431.1922	195	17.61	17.2	108	7.66E-03
431.3012	22	15.03	13.54	545	3.83E-02
432.8201	176	16.12	16.68	693	4.79E-02
432.9339	132	15.82	16.61	203	1.45E-02
434.9732	31	18.47	18.79	365	2.53E-02
435.2414	170	19.16	19.63	596	4.14E-02
435.3061	193	17	16.12	128	9.10E-03

436.02	128	16.91	15.99	639	4.43E-02
436.2553	146	16.8	16.43	631	4.39E-02
436.9641	114	16.08	14.65	482	3.39E-02
437.2218	151	17.26	17.85	573	3.96E-02
439.2824	197	18.39	17.99	511	3.58E-02
442.2094	161	17.66	17.47	132	9.29E-03
442.2826	144	12.84	14.43	335	2.34E-02
442.885	44	14.34	15.15	370	2.57E-02
444.9347	143	17.4	17.66	563	3.91E-02
445.2085	269	17.06	16.72	518	3.65E-02
446.8532	147	15.64	16.52	696	4.80E-02
447.2613	214	21.6	21.92	475	3.30E-02
448.2648	217	19.32	19.66	470	3.27E-02
449.1524	17	15.63	16.31	477	3.35E-02
452.2783	203	24.56	24.08	591	4.07E-02
453.2347	162	16.85	16.47	123	8.67E-03
453.2535	175	18.37	18.51	517	3.64E-02
453.2721	224	16.53	15.69	404	2.81E-02
453.3231	126	16.34	16.08	390	2.73E-02
456.9837	31	14.02	16.12	49	3.59E-03
459.2615	16	13.56	14.63	692	4.77E-02
460.2655	159	16.34	15.83	622	4.34E-02
462.2717	210	18.48	18.09	235	1.71E-02
462.9098	22	16.76	16.14	159	1.11E-02
462.9322	125	17.56	16.84	298	2.10E-02
463.2924	267	17.75	17.17	326	2.27E-02
464.8819	148	16.03	16.52	17	1.17E-03
465.2359	125	19.42	19.28	706	4.89E-02
469.3013	269	17.45	16.96	618	4.33E-02
470.2787	270	17.61	17.04	306	2.14E-02
472.8754	36	15.87	15.35	103	7.44E-03
473.3645	233	21.04	21.73	621	4.34E-02
474.3679	233	18.6	19.62	290	2.05E-02
478.882	147	16.88	17.3	568	3.93E-02
478.9058	141	18.05	17.6	65	4.67E-03
479.073	17	17.91	18.16	576	3.98E-02
479.5736	16	15.73	16.29	483	3.40E-02
480.9714	285	17.8	17.14	6	4.88E-04

480.9718	184	17.66	17.3	471	3.29E-02
481.2823	199	18.15	17.73	273	1.98E-02
481.3145	180	19.04	18.42	292	2.07E-02
483.0464	81	17.16	16.73	101	7.33E-03
483.9483	32	15.83	16.31	382	2.67E-02
484.3682	178	15.64	14.48	89	6.47E-03
486.8911	36	15.47	15.02	321	2.22E-02
489.2127	118	17.03	17.94	658	4.55E-02
489.2308	178	17.31	17.93	539	3.80E-02
491.8425	47	12.51	13.47	581	4.01E-02
492.8571	31	13.73	14.46	627	4.37E-02
493.0011	30	16.37	17.27	360	2.48E-02
493.2304	155	18.19	17.75	100	7.33E-03
493.3185	170	16.52	16.06	171	1.20E-02
493.9941	128	17.57	16.48	315	2.19E-02
494.2898	197	16.33	15.54	461	3.23E-02
495.243	269	19.5	19.23	245	1.80E-02
495.3338	165	17.83	17.49	492	3.45E-02
495.4429	222	16.8	16	183	1.29E-02
495.5507	16	12.76	13.38	182	1.29E-02
496.0498	176	19.2	18.83	430	3.00E-02
497.047	175	18.45	18.04	284	2.03E-02
497.2772	160	18.11	17.46	210	1.50E-02
501.235	169	18.34	17.89	358	2.47E-02
502.0325	24	15.73	15.11	528	3.72E-02
502.8763	146	18.16	17.83	217	1.54E-02
504.0086	70	15.58	16.21	452	3.15E-02
504.3109	258	20.53	18.06	158	1.11E-02
505.3373	168	21.21	20.58	565	3.91E-02
505.9011	175	14.73	15.41	543	3.82E-02
506.8481	52	12.9	13.95	70	5.13E-03
507.021	55	17.63	18	214	1.53E-02
507.2816	192	16.69	16.13	369	2.57E-02
507.3161	172	17.06	16.58	468	3.26E-02
507.3201	130	17.9	17.52	272	1.98E-02
507.3424	168	14.49	13.47	630	4.39E-02
508.3241	130	16.12	15.37	122	8.63E-03
509.2879	143	21.44	21.18	35	2.45E-03

509.3337	180	22.3	21.73	600	4.15E-02
510.2929	143	19.61	19.36	2	2.89E-04
510.3358	180	20.89	20.26	197	1.39E-02
511.2567	154	17.12	16.91	701	4.87E-02
511.2935	150	17.55	17.13	30	1.96E-03
511.3388	179	17.72	17.08	681	4.71E-02
512.3669	158	15.65	16.31	389	2.73E-02
517.2559	154	15.53	14.93	657	4.55E-02
520.2669	202	18.78	18.18	274	1.98E-02
521.0357	46	16.45	16.81	677	4.69E-02
523.3126	129	16.27	15.84	547	3.84E-02
523.365	193	17.68	17.9	493	3.46E-02
524.8455	157	15.28	15.92	323	2.22E-02
524.8471	34	11.86	13.37	151	1.07E-02
525.2408	217	18.04	18.28	680	4.71E-02
529.2753	225	19.73	19.09	416	2.90E-02
531.2254	173	15.86	15.5	523	3.70E-02
532.8227	43	13.26	13.84	562	3.90E-02
532.9812	127	16.59	15.85	408	2.83E-02
533.3132	143	15.26	13.95	664	4.61E-02
533.4198	67	14.68	15.18	324	2.25E-02
534.2828	202	18.83	18.09	420	2.91E-02
534.9202	167	15.25	16.3	425	2.99E-02
535.2878	200	16.26	15.48	336	2.34E-02
535.3157	208	20.15	19.19	269	1.97E-02
539.3197	205	20.08	18.99	682	4.71E-02
539.3437	133	16.99	16.47	455	3.18E-02
541.2162	212	17.16	16.5	714	4.97E-02
541.2674	21	20.74	21.45	98	7.28E-03
541.3039	198	17.14	16.81	464	3.25E-02
541.3752	187	17.67	17.43	320	2.22E-02
541.9672	82	15.9	15.05	331	2.31E-02
542.2704	22	18.56	19.52	111	7.93E-03
543.3388	170	15.41	12.96	14	1.03E-03
545.9496	76	16.06	15.69	558	3.88E-02
547.0141	29	14.24	14.72	177	1.25E-02
547.143	18	13.01	13.84	243	1.80E-02
551.3483	169	17.17	16.78	287	2.05E-02

552.8758	136	16.61	17.04	215	1.53E-02
555.2808	160	16.75	16.46	536	3.78E-02
555.9927	104	16.75	17.17	703	4.88E-02
556.2688	178	14.65	13.19	230	1.69E-02
557.3568	176	16.19	15.2	68	4.93E-03
557.3639	147	16.84	16.3	484	3.40E-02
558.3394	152	16.28	16.03	293	2.08E-02
561.4806	218	16.35	18.13	343	2.38E-02
563.1081	49	11.76	12.46	199	1.39E-02
563.3466	188	18.62	17.86	427	2.99E-02
563.3845	159	15.29	14.6	66	4.70E-03
564.9122	57	16.75	16.31	652	4.53E-02
567.2447	167	15.79	16.05	206	1.46E-02
568.9017	105	16.31	15.92	675	4.67E-02
569.8783	59	15.35	15.81	467	3.26E-02
570.8062	40	14.52	15.58	377	2.63E-02
573.9252	60	16.44	16.27	421	2.93E-02
575.0098	35	16.94	17.58	152	1.08E-02
576.4567	235	14.17	14.61	497	3.48E-02
576.873	125	16.55	15.87	327	2.27E-02
577.2772	144	15.22	14.31	667	4.63E-02
578.8061	52	14.64	13.67	148	1.04E-02
578.9667	109	17.82	18.09	204	1.45E-02
582.4805	89	15.29	14.59	673	4.66E-02
583.9153	108	15.65	15	232	1.70E-02
589.0252	42	16.06	16.42	254	1.86E-02
589.3246	151	17.18	16.83	516	3.61E-02
590.9213	106	15.34	16.06	307	2.14E-02
593.3983	168	17.38	16.49	432	3.02E-02
594.3016	194	13.66	12.47	567	3.93E-02
594.9202	80	17.09	16.74	196	1.37E-02
596.3578	158	16.11	14.85	262	1.94E-02
597.2444	135	14.89	13.09	304	2.13E-02
597.2722	214	15.47	15.89	480	3.38E-02
597.4315	214	21.5	22.35	678	4.69E-02
597.8522	58	15.49	15.14	413	2.88E-02
599.2278	22	13.96	14.91	334	2.34E-02
599.4346	218	14.55	15.42	346	2.41E-02

601.4132	162	17.99	17.45	227	1.67E-02
601.8397	55	16.41	15.32	223	1.62E-02
602.3265	147	15.69	16.06	308	2.15E-02
602.8881	63	16.05	15.67	524	3.71E-02
603.0432	40	14.92	15.37	83	6.20E-03
604.8418	40	15.49	14.51	137	9.65E-03
605.1543	15	18.91	19.36	352	2.46E-02
605.1573	54	14.37	15.37	86	6.32E-03
605.3194	152	17.05	16.83	595	4.13E-02
605.3512	210	14.92	13.7	300	2.11E-02
606.3777	208	13.28	14.87	428	2.99E-02
607.1461	15	14.35	15.03	62	4.46E-03
607.3494	123	16.77	15.75	405	2.81E-02
608.3202	194	16.95	16.07	180	1.27E-02
608.8279	149	14.71	15.23	440	3.06E-02
609.4086	73	15.07	15.71	79	5.93E-03
609.8935	69	15.22	15.71	67	4.85E-03
609.8975	68	15.54	15.91	88	6.41E-03
610.8891	50	15.78	15.1	117	8.37E-03
610.9603	59	15.69	16.08	647	4.48E-02
614.0011	90	15.9	15.1	479	3.38E-02
614.8718	54	15.61	14.97	553	3.87E-02
615.3735	59	13.37	13.86	614	4.32E-02
616.0289	24	13	14.08	498	3.48E-02
616.4176	78	15.49	16.18	59	4.36E-03
617.8271	53	14.2	14.46	684	4.73E-02
618.805	50	11.36	12.49	491	3.44E-02
619.8682	56	15.12	15.5	21	1.26E-03
619.9325	75	15.65	14.76	398	2.78E-02
621.4139	81	15.62	16.14	238	1.78E-02
621.4328	210	15.24	16.23	466	3.26E-02
622.332	184	17.42	16.53	92	6.55E-03
624.1339	16	13.03	13.6	16	1.12E-03
627.1164	17	21.97	22.33	604	4.19E-02
627.4361	85	15.98	15.2	340	2.37E-02
628.1165	17	20.75	21.07	620	4.33E-02
629.1168	17	20.62	20.89	140	9.88E-03
629.39	172	16.11	15.95	670	4.65E-02

629.92	80	17.63	17.32	469	3.27E-02
630.1174	17	18.64	19.04	58	4.32E-03
630.8651	47	14.48	15.05	400	2.80E-02
631.1075	17	12.52	13.12	174	1.23E-02
631.9145	84	15.94	16.22	94	6.81E-03
633.8997	108	16.04	15.34	485	3.41E-02
634.0928	16	13.6	14.55	150	1.06E-02
635.0966	16	13.46	14.06	561	3.90E-02
635.6081	17	13.64	14.19	248	1.82E-02
636.0882	15	19.95	20.39	149	1.05E-02
636.3507	182	19.22	18.49	412	2.87E-02
636.6029	17	12.53	13.37	169	1.19E-02
637.0921	15	18.01	18.53	74	5.48E-03
637.3493	180	15.94	15.45	5	4.55E-04
637.8807	109	16.17	15.75	381	2.66E-02
638.094	15	13.29	14.53	34	2.37E-03
638.8612	179	17.61	15.59	296	2.10E-02
640.542	214	15.99	16.43	305	2.14E-02
640.9215	68	15.84	15.52	242	1.79E-02
643.0776	17	14.16	14.52	247	1.81E-02
643.0956	17	23.81	24.08	698	4.81E-02
643.4848	84	16.18	15.63	136	9.47E-03
643.593	16	17.18	17.61	486	3.42E-02
644.5902	16	12.89	13.31	301	2.12E-02
644.8735	69	15.25	15.55	423	2.97E-02
644.8847	105	14.74	15.69	325	2.26E-02
646.823	156	14.08	14.82	27	1.73E-03
648.0854	17	13.74	14.09	234	1.71E-02
648.3479	188	15.74	14.41	78	5.93E-03
648.852	112	15.23	14.8	679	4.70E-02
648.9121	70	15.07	15.72	632	4.39E-02
650.9045	163	13.66	14.64	36	2.45E-03
652.0828	16	16.94	17.16	611	4.25E-02
652.8743	52	15.08	14.81	594	4.12E-02
652.9034	66	15.51	16.06	542	3.82E-02
653.0773	16	13.07	13.43	711	4.95E-02
653.5108	214	14.31	14.95	661	4.58E-02
653.5198	217	19.89	20.27	559	3.89E-02

653.9255	81	15.89	15.44	355	2.47E-02
654.0759	17	12.98	13.37	291	2.06E-02
655.0062	93	16.1	15.46	556	3.87E-02
655.0817	17	12.92	13.71	449	3.15E-02
655.4136	74	14.42	13.97	317	2.20E-02
655.4384	80	16.09	15.03	143	9.94E-03
657.4427	85	15.5	14.98	271	1.98E-02
657.4496	81	16.09	15.42	519	3.66E-02
657.8862	66	15.94	15.41	250	1.84E-02
658.0608	17	14.53	15.08	193	1.34E-02
658.0722	24	13.38	14.55	139	9.84E-03
659.8883	105	15.98	15.47	24	1.37E-03
661.9144	92	15.24	15.54	313	2.19E-02
662.8876	114	15.13	14.17	507	3.57E-02
663.3643	171	14.49	15.26	712	4.95E-02
663.4056	77	15.01	15.6	224	1.64E-02
664.9074	95	16.16	16.48	302	2.12E-02
665.0897	22	14.55	14.96	601	4.16E-02
665.4212	214	15.91	16.71	80	5.97E-03
667.0529	16	12.75	13.34	4	3.13E-04
667.3095	147	13.15	14.84	167	1.18E-02
668.4606	89	15.45	14.73	395	2.74E-02
670.3387	215	16.65	16	608	4.22E-02
671.5305	217	19.03	19.42	709	4.93E-02
672.0639	16	15.53	16.32	540	3.81E-02
672.1411	166	15.91	14.77	541	3.81E-02
674.0351	15	18.72	19.16	134	9.44E-03
675.0415	15	19.21	19.53	208	1.48E-02
675.2324	279	15.33	15.76	362	2.52E-02
675.9043	104	16.17	15.67	54	3.82E-03
676.3034	288	13.63	14.71	520	3.67E-02
676.9553	75	16.75	16.37	610	4.24E-02
678.3422	189	17.46	16.69	496	3.47E-02
678.397	208	17.01	16.39	451	3.15E-02
679.4297	214	15.09	16.29	13	8.57E-04
679.4415	217	14.31	15.54	156	1.10E-02
682.8507	143	15.76	16.04	487	3.42E-02
683.038	16	16.58	16.85	638	4.43E-02

684.3594	212	14.8	14.07	97	7.13E-03
685.354	213	16.78	15.37	578	3.99E-02
685.5263	217	26.67	27	260	1.90E-02
686.5307	214	25.5	25.84	396	2.75E-02
686.8433	54	15.46	15.19	173	1.23E-02
687.3751	283	13.35	13.92	506	3.55E-02
687.5287	217	23.34	23.7	580	4.00E-02
691.9055	139	14.69	15.15	499	3.48E-02
692.5508	269	12.97	13.86	200	1.43E-02
693.7752	57	13.3	14.87	47	3.31E-03
693.8758	103	15.91	16.25	91	6.50E-03
694.9579	141	16.98	17.08	546	3.84E-02
696.0048	89	16.04	15.01	23	1.35E-03
697.3153	231	14.63	14.2	688	4.75E-02
697.9406	89	13.78	14.89	268	1.97E-02
697.9656	58	15.03	14.51	347	2.41E-02
700.3348	213	13.42	14.39	574	3.97E-02
700.8823	103	15.8	15.29	319	2.21E-02
701.5203	213	15.95	16.42	239	1.78E-02
701.5303	217	15.28	15.88	209	1.50E-02
701.867	67	15	15.38	283	2.03E-02
702.7661	48	14.12	15.29	60	4.38E-03
703.0338	17	14.61	14.8	316	2.20E-02
703.9452	85	15.49	15.11	535	3.77E-02
703.9578	82	14.9	15.49	549	3.84E-02
711.9661	80	17.34	16.89	674	4.67E-02
712.8464	53	15.48	15.81	213	1.53E-02
712.9767	85	16.21	15.55	333	2.32E-02
714.8738	171	12.36	13.41	454	3.17E-02
714.8762	74	14.77	15.22	82	6.20E-03
714.8944	176	10.75	11.44	502	3.51E-02
716.4124	174	15.86	15.38	189	1.32E-02
716.9184	177	14.1	12.5	700	4.87E-02
717.4585	90	15.11	15.46	478	3.36E-02
720.8023	54	13.95	14.72	46	3.23E-03
721.9071	85	16.12	15.8	205	1.45E-02
727.919	70	16.01	16.57	3	2.93E-04
727.9552	106	16.72	16.35	51	3.64E-03

736.9322	96	15.94	16.53	584	4.04E-02
738.7975	156	15.32	15.78	87	6.34E-03
740.9229	57	15.93	15.35	458	3.22E-02
741.8872	75	15.16	15.68	259	1.90E-02
742.2752	121	11.84	12.75	384	2.69E-02
744.9448	79	14.44	15.16	270	1.97E-02
747.3326	187	12.38	10.48	349	2.45E-02
750.8605	153	15.38	15.78	178	1.26E-02
753.9449	94	15.94	15.78	153	1.08E-02
754.886	60	16.03	16.57	448	3.14E-02
757.5672	217	20.96	21.29	666	4.61E-02
758.029	15	11.47	12.02	435	3.03E-02
760.5759	245	15.87	16.07	552	3.86E-02
760.8711	68	16.07	15.35	43	2.97E-03
764.5149	215	16.51	16.8	249	1.84E-02
764.8356	53	14.73	15.28	419	2.91E-02
766.9204	83	15.91	15.67	515	3.61E-02
766.9834	127	17.53	15.88	133	9.43E-03
770.912	91	15.84	16.19	126	8.89E-03
771.9278	99	15.5	15.97	275	1.98E-02
772.9988	89	15.56	15.22	710	4.94E-02
773.5443	198	16.42	16	31	2.04E-03
776.5704	236	15.27	15.94	141	9.91E-03
776.9016	84	15.29	15.94	644	4.43E-02
777.5412	190	16.11	15.65	635	4.40E-02
779.0156	16	13.57	13.96	414	2.89E-02
780.936	82	15.78	16.61	583	4.02E-02
781.572	211	14.29	13.66	570	3.94E-02
781.9182	81	15.81	16.26	256	1.86E-02
785.9389	94	16.47	15.76	651	4.53E-02
786.9778	88	15.36	15.9	244	1.80E-02
788.8225	131	16.74	16.95	191	1.33E-02
788.8517	110	15.29	15.7	329	2.30E-02
790.8255	51	14.28	14.98	7	5.99E-04
790.9241	97	15.69	16.02	593	4.11E-02
796.8952	94	15.27	15.85	612	4.26E-02
798.7869	141	16	15.63	397	2.78E-02
798.9519	92	14.75	15.43	25	1.37E-03

802.8148	56	15.39	14.99	261	1.92E-02
802.8208	58	14.52	14.14	130	9.12E-03
802.8371	138	16.72	16.04	364	2.52E-02
802.8912	101	15.52	15.94	279	2.00E-02
804.9864	92	15.43	14.76	393	2.73E-02
808.6155	16	12.79	13.4	278	2.00E-02
810.5416	203	14.15	14.69	699	4.83E-02
816.5892	281	13.28	14.83	407	2.83E-02
822.7904	162	15.1	15.62	90	6.49E-03
823.0119	92	15.71	14.28	222	1.60E-02
824.0205	87	15.14	14.62	374	2.62E-02
824.8433	60	13.71	14.72	29	1.93E-03
824.9322	84	15.29	15.84	575	3.98E-02
826.9297	90	15.78	15.28	37	2.56E-03
828.5595	184	17.28	16.49	554	3.87E-02
829.9984	90	15.8	16.15	654	4.54E-02
830.8784	111	15.56	14.91	589	4.07E-02
832.92	71	14.04	14.97	363	2.52E-02
835.5491	214	21.48	21.86	495	3.47E-02
836.577	185	15.95	15.52	691	4.77E-02
836.9894	131	17.35	13.87	81	6.08E-03
839.0209	91	15.1	15.7	628	4.37E-02
842.8928	102	16.1	16.5	107	7.61E-03
846.831	62	14.07	14.8	357	2.47E-02
856.7894	55	13.66	14.39	602	4.17E-02
856.8638	105	15.42	16.11	187	1.32E-02
863.9283	84	16.17	15.9	525	3.71E-02
872.553	180	14.96	15.23	433	3.02E-02
876.8371	112	15.48	15.12	361	2.48E-02
892.6224	197	15.36	15.91	350	2.45E-02
892.9086	111	17.35	17.76	339	2.37E-02
895.0215	131	17.62	15.35	332	2.32E-02
904.9271	82	15.52	15.91	522	3.69E-02
907.5304	201	15.52	14.62	503	3.52E-02
908.9263	89	16.24	16.36	689	4.75E-02
911.5575	213	15.27	15.9	265	1.95E-02
914.8202	62	15.07	15.71	75	5.64E-03
918.5674	181	15.31	15.84	19	1.22E-03

918.5711	183	15.93	16.31	162	1.13E-02
920.2135	15	12.17	13.69	8	6.64E-04
924.5517	186	15.6	15.91	521	3.68E-02
924.6045	186	16.56	16.41	411	2.85E-02
934.8685	109	15.19	13.7	64	4.62E-03
938.57	188	14.82	15.17	550	3.85E-02
941.1837	17	13.12	13.87	121	8.48E-03
942.1803	17	12.82	13.98	76	5.76E-03
942.8715	72	14.43	15.08	551	3.86E-02
942.9412	91	15.7	16.21	165	1.16E-02
948.0445	90	15.47	14.98	537	3.79E-02
957.1631	16	15.19	15.74	289	2.05E-02
957.9742	87	15.41	15.13	577	3.99E-02
958.1614	16	15.04	15.62	55	4.13E-03
959.1572	16	14.99	15.52	72	5.32E-03
960.1586	16	13.74	14.3	144	1.01E-02
973.1129	16	11.71	12.56	241	1.79E-02
974.1402	15	15.53	15.99	267	1.95E-02
975.1244	15	14.83	15.27	84	6.24E-03
976.1385	15	13.27	14.21	10	7.50E-04
979.1373	17	13.19	13.98	1	1.62E-04
990.1165	15	15.11	15.47	276	1.98E-02
991.1164	15	13.67	13.99	257	1.87E-02
993.1085	15	12.78	13.22	114	8.24E-03
995.1149	16	14.06	14.39	142	9.92E-03
998.5658	181	15.71	14.7	179	1.27E-02
1011.0769	16	13.89	14.39	406	2.82E-02
1015.8179	236	14.12	15.03	436	3.03E-02
1018.9097	94	15.51	15.89	399	2.79E-02
1022.9456	91	15.52	15.3	457	3.22E-02
1037.806	217	17.91	18.49	474	3.30E-02
1038.7938	217	15.48	16.05	207	1.48E-02
1043.0524	93	15.93	14.84	95	6.82E-03
1064.8209	214	15.59	16.39	44	2.97E-03
1065.8331	214	16.13	16.96	20	1.25E-03
1071.0964	15	13.41	13.71	564	3.91E-02

Raw p values < 0.05
ESI=electrospray ionization
TPP = thiamine pyrophosphate

Supplemental Table 4. Significant metabolic features linked to TPP concentrations from the HILIC positive ESI column: lowest versus highest tertile

m/z	Time (sec)	Mean Highest tertile (normalized)	Mean Lowest tertile (normalized)	Differential expression rank	P value
85.0285	36	19.04	18.52	503	1.21E-04
85.0285	286	15.12	14.98	10837	4.03E-04
85.0285	104	18.32	18.4	12656	4.22E-04
85.0479	101	15.96	16.26	10680	4.67E-04
85.0588	33	16.13	15.8	11959	4.85E-04
85.0649	265	16.86	16.8	10733	5.27E-04
85.065	10	16.28	16.35	17081	5.38E-04
85.1714	34	15.7	15.99	11747	5.45E-04
85.1753	44	20.72	20.38	7679	5.76E-04
85.2867	42	18.24	18.36	3220	6.03E-04
85.3263	24	20.26	20.28	16783	6.23E-04
85.3953	34	17.15	17.12	9963	6.57E-04
85.3992	48	21.3	21.1	12830	6.76E-04
85.5106	49	19.39	19.28	12564	6.83E-04
85.5661	33	16.29	16.25	9313	7.16E-04
85.6507	100	18.84	18.16	1663	7.49E-04
85.843	47	19.81	19.62	14164	8.02E-04
86.0601	84	18.44	18.76	5161	8.80E-04
86.0602	281	15.49	15.67	4909	8.85E-04
86.0714	78	16.14	16.59	6871	8.99E-04
86.0965	65	23.47	23.27	7201	1.07E-03
86.4947	50	17.34	17.47	16439	1.18E-03
86.5023	59	16.48	16.38	17359	1.29E-03
86.7167	50	18.6	18.61	10154	1.37E-03
86.7285	51	23.42	23.26	11630	1.38E-03
86.7287	97	18.65	18.55	13313	1.39E-03
86.784	51	16.71	16.93	16502	1.40E-03
86.84	51	22.38	22.17	6883	1.45E-03
86.9387	50	18.88	18.87	7473	1.50E-03
86.9523	43	25.27	24.86	3509	1.52E-03
87.0264	68	17.59	17.79	13420	1.52E-03
87.0442	260	15.48	15.24	10841	1.67E-03
87.0442	11	14.85	15.1	2456	1.79E-03

87.0642	43	24.54	24.04	2513	1.81E-03
87.0999	64	19.07	18.86	7260	1.83E-03
87.1605	51	18.46	18.46	9965	1.83E-03
87.1766	45	24.39	24.11	4611	1.85E-03
87.2878	45	22.73	22.49	4204	1.88E-03
87.3821	51	17.21	17.25	13660	1.88E-03
87.4006	46	24.35	24.34	15764	1.94E-03
87.4255	104	17.35	17.35	16509	2.00E-03
87.5121	46	23.08	23.02	15516	2.08E-03
87.6236	47	21.41	21.14	2845	2.32E-03
88.0579	92	16.45	16.64	3733	2.37E-03
88.0616	50	17.27	16.65	6899	2.41E-03
88.0658	43	18.93	18.77	15167	2.53E-03
88.0758	283	15.7	15.97	17662	2.56E-03
88.0759	10	14.82	14.88	13703	2.73E-03
88.2899	46	21.73	21.54	9728	2.74E-03
88.4013	45	20.4	20.13	12106	2.79E-03
88.5035	89	18.8	18.87	13512	2.83E-03
88.5097	42	18.16	17.38	1952	2.90E-03
88.5136	49	19.04	18.84	12956	2.91E-03
88.6149	47	21.36	20.93	8242	2.91E-03
88.7256	47	18.24	18.14	12465	3.02E-03
88.7272	47	20.9	20.38	5092	3.07E-03
88.753	87	16.3	16.56	5285	3.17E-03
88.8387	47	18.87	18.56	4927	3.19E-03
88.9812	295	14.2	14.19	11323	3.26E-03
88.9931	33	19.77	19.89	14481	3.35E-03
89.042	68	17.69	17.41	8079	3.35E-03
89.0598	30	15.72	16.06	16453	3.36E-03
89.0599	276	14.8	14.41	5131	3.41E-03
89.1075	285	15.07	14.96	12831	3.50E-03
89.3953	94	16.36	15.31	3721	3.52E-03
89.5064	54	20.51	20.02	2379	3.53E-03
89.5069	290	16.17	16.53	3653	3.53E-03
89.6194	43	24.22	23.78	3266	3.77E-03
89.7309	43	22.96	22.46	2820	3.78E-03
89.8291	24	20.19	20.21	15108	3.78E-03
89.8433	44	23.33	22.95	527	3.84E-03
89.9547	44	22.02	21.62	541	3.85E-03

90.0374	10	14.32	14.26	9329	3.85E-03
90.0551	96	19.95	19.84	10470	3.88E-03
90.0603	48	17.26	17.34	12937	3.88E-03
90.0673	43	23.63	23.26	3797	3.94E-03
90.1788	44	22.48	22.08	2187	3.96E-03
90.2906	44	21.25	20.56	665	4.01E-03
90.4024	45	18.8	18.13	867	4.05E-03
90.5071	294	14.66	14.55	2046	4.06E-03
90.7316	100	17.87	17.34	3135	4.11E-03
90.9479	292	16.46	16.8	7321	4.13E-03
90.9768	106	24.39	24.25	7478	4.28E-03
90.9889	34	16.07	15.98	9544	4.36E-03
91.0292	89	16.86	16.69	15212	4.39E-03
91.0293	10	16.04	16.07	15917	4.44E-03
91.0398	68	18.41	18.41	16620	4.52E-03
91.0544	72	21.4	21.42	11069	4.57E-03
91.0575	10	15.18	15.2	9692	4.59E-03
91.1701	47	21.08	20.73	7828	4.72E-03
91.2815	47	19.69	19.18	4176	4.75E-03
91.394	47	19.83	19.51	5844	4.77E-03
91.5054	47	18.38	18.33	8632	4.80E-03
91.618	47	19.89	19.67	10052	4.88E-03
91.7293	47	18.65	18.5	7162	4.93E-03
91.9802	103	17.08	16.91	14142	4.99E-03
92.022	31	18.03	17.62	10281	5.09E-03
92.037	32	17.82	17.92	16089	5.13E-03
92.0495	30	18.24	17.8	3589	5.16E-03
92.0577	73	17.35	17.54	7757	5.17E-03
92.0577	287	15.76	15.91	15927	5.18E-03
92.0616	51	19.63	19.27	7505	5.20E-03
92.0699	33	16.61	16.47	9291	5.29E-03
92.173	52	17.77	17.41	12866	5.34E-03
92.2855	52	18.41	17.91	4080	5.35E-03
92.3381	24	20.66	20.64	12872	5.38E-03
92.5028	50	16.27	15.86	6263	5.54E-03
92.5097	49	20.24	19.71	2104	5.63E-03
92.5736	33	16.12	16.03	10685	5.66E-03
92.5888	25	18.18	18.12	13349	5.67E-03
92.621	50	18.29	18.07	11477	5.69E-03

92.7339	43	21.13	20.63	5454	5.73E-03
92.8451	42	19.35	18.81	4674	5.87E-03
92.9576	42	17.85	17.28	8728	5.97E-03
93.0368	281	15.1	15.03	12025	5.99E-03
93.0368	11	15.14	15.26	14374	6.04E-03
93.0449	10	14.95	14.99	15153	6.04E-03
93.0449	298	17.45	16.83	2451	6.08E-03
93.0574	29	17.71	18.11	4849	6.19E-03
93.07	62	18.87	18.95	17450	6.22E-03
93.07	172	19.29	19.2	16351	6.31E-03
93.0701	292	17.75	18.04	13413	6.44E-03
93.168	51	18.59	18.42	6911	6.60E-03
93.2049	100	17.94	17.48	5937	6.63E-03
93.2795	51	16.15	16.63	9756	6.66E-03
93.3785	50	15.67	15.49	3760	6.66E-03
93.8363	47	18.67	18.31	4159	6.73E-03
93.9479	47	17.62	17.38	13713	6.80E-03
94.0401	268	14.82	14.79	6317	6.81E-03
94.0402	10	14.28	14.32	8686	6.83E-03
94.0483	297	13.55	12.89	3263	6.90E-03
94.0527	10	13.6	13.58	11019	6.91E-03
94.0603	47	17.68	17.65	8302	6.92E-03
94.0652	64	18.81	19.14	2339	6.93E-03
94.0653	292	17.23	17.03	2071	6.96E-03
94.0734	10	13.99	14.15	16510	6.96E-03
94.2825	98	15.52	15.44	16593	7.06E-03
94.2843	47	18.27	18.44	4350	7.13E-03
94.3959	47	17.47	17.04	1708	7.16E-03
94.7251	112	17.19	16.97	6361	7.17E-03
94.9796	104	16.92	17	16584	7.20E-03
95.0241	73	18.52	18.76	4868	7.22E-03
95.0242	20	19.1	19.02	10802	7.34E-03
95.0493	39	19	19.38	9593	7.38E-03
95.0493	73	20.79	20.83	15528	7.38E-03
95.0493	293	17.86	18.26	2953	7.72E-03
95.0605	217	17.78	17.63	9149	7.74E-03
95.0605	11	16.53	16.53	13385	7.79E-03
95.0856	24	21.49	21.58	9889	7.82E-03

95.0856	71	21.15	20.83	878	8.01E-03
96.0213	28	19.35	19.43	6940	8.03E-03
96.0275	10	13.83	13.49	14220	8.14E-03
96.0445	205	20.01	20.13	3401	8.16E-03
96.0445	78	18.37	18.26	5691	8.19E-03
96.0445	27	19.02	18.86	9086	8.24E-03
96.0526	73	16.8	16.74	9592	8.24E-03
96.0527	10	14.32	14.38	16344	8.25E-03
96.0558	10	16.5	16.49	14762	8.26E-03
96.0639	297	14.49	14.24	15698	8.31E-03
96.0809	74	17.13	16.73	5690	8.32E-03
96.0809	281	14.92	15.36	9697	8.35E-03
96.0809	15	16.01	16.26	11058	8.36E-03
96.089	85	16.94	16.9	13531	8.40E-03
96.089	15	17.17	17.06	6694	8.43E-03
96.2715	28	18.71	17.96	4362	8.46E-03
96.5153	20	12.91	13.77	984	8.46E-03
96.7178	47	16.81	17.19	7957	8.55E-03
96.8478	44	17.44	17.62	16012	8.61E-03
96.9219	49	21.06	21.25	2609	8.71E-03
96.961	60	17.73	17.59	15417	8.76E-03
96.9611	289	17.56	17.73	420	8.76E-03
97.0078	297	18.06	18.33	6224	8.78E-03
97.0285	81	17.32	17.2	17300	8.88E-03
97.0286	288	16.11	16	611	8.91E-03
97.0286	10	16.23	16.24	17238	8.92E-03
97.0397	75	18.68	18.76	17452	8.96E-03
97.0398	223	20.58	20.56	16082	8.96E-03
97.0398	11	19.32	19.09	1139	9.02E-03
97.0479	281	14.52	14.84	12968	9.08E-03
97.0479	9	14.25	14.23	10109	9.11E-03
97.0649	26	20.95	20.69	2054	9.18E-03
97.0649	68	20.16	20.29	13893	9.22E-03
97.0762	9	16.59	16.65	5071	9.24E-03
97.1013	82	17.38	17.4	12150	9.26E-03
97.1013	23	20.15	20.28	9577	9.30E-03
97.9689	8	14.35	14.42	12034	9.42E-03
97.9915	298	18.78	18.74	14545	9.43E-03

98.0431	273	14.7	14.77	16589	9.43E-03
98.0432	9	14.41	14.4	15381	9.44E-03
98.0578	91	16.64	16.55	15433	9.59E-03
98.0602	32	18.76	18.52	11937	9.64E-03
98.0683	281	15.73	16.04	12898	9.65E-03
98.0683	10	16.37	16.42	15187	9.72E-03
98.0714	286	13.88	15.01	9	9.78E-03
98.0715	13	15.24	15.5	1898	9.83E-03
98.0965	71	19.3	19.94	1753	1.00E-02
98.5121	71	17.43	17.43	17492	1.00E-02
98.5122	284	15.42	15.21	4825	1.00E-02
98.5122	12	14.02	14.01	14549	1.01E-02
98.9189	50	19.8	19.96	4066	1.01E-02
98.919	134	17.32	17.48	13684	1.03E-02
98.9615	297	17.62	17.21	323	1.03E-02
98.9615	9	14.49	14.53	7908	1.04E-02
98.9843	75	18.63	18.49	4353	1.04E-02
99.0441	76	18.62	18.54	13335	1.05E-02
99.0554	85	17.71	17.78	10701	1.05E-02
99.0636	13	14.59	14.21	12596	1.05E-02
99.0636	277	14.91	14.53	2971	1.06E-02
99.0805	22	17.8	17.6	6712	1.07E-02
99.0806	83	17.51	17.91	6829	1.09E-02
99.0918	83	17.92	18.04	11424	1.09E-02
99.0918	283	16.83	16.81	14205	1.09E-02
99.0918	11	14.86	15.03	9893	1.09E-02
99.0999	72	16.46	16.95	17445	1.10E-02
99.5124	294	15.48	15.26	1607	1.10E-02
100.0194	25	19.34	18.93	6302	1.10E-02
100.0216	67	17.85	17.62	6663	1.10E-02
100.0394	87	16.61	16.21	8637	1.11E-02
100.0394	10	16.74	16.61	5978	1.11E-02
100.0475	11	14.92	15	13135	1.12E-02
100.0507	86	15.66	16.17	660	1.13E-02
100.0507	283	14.94	15.22	6260	1.15E-02
100.0507	11	15.14	15.25	13810	1.16E-02
100.058	10	14.38	13.98	7795	1.17E-02
100.0758	32	24.03	24.31	11936	1.18E-02

100.0855	32	17.58	17.46	6638	1.18E-02
100.1121	40	19.18	18.87	883	1.18E-02
100.1122	214	20.66	20.35	5359	1.19E-02
100.5101	295	14.73	15.2	1541	1.19E-02
100.7591	100	16.14	16.69	13972	1.20E-02
100.9569	295	16.51	16.19	691	1.21E-02
101.0347	87	17.28	16.99	4116	1.21E-02
101.0347	10	16.79	16.75	6861	1.22E-02
101.0598	82	17.53	17.76	5717	1.22E-02
101.0598	20	19.34	19.25	17240	1.22E-02
101.0711	30	17.08	17.22	12422	1.22E-02
101.0711	275	14.96	15.19	1912	1.23E-02
101.0711	101	17.14	17.28	12666	1.23E-02
101.0791	29	19.99	20.06	7958	1.24E-02
101.0792	276	16.08	16.41	335	1.24E-02
101.1156	264	15.37	15.53	4297	1.25E-02
101.1156	9	14.22	14.13	9081	1.25E-02
102.0131	67	18.44	18.77	72	1.26E-02
102.0132	298	21.75	21.97	4864	1.27E-02
102.0465	61	18.13	18.07	6120	1.27E-02
102.0551	91	19.92	20.11	7436	1.27E-02
102.0551	291	17.26	17.19	13442	1.28E-02
102.0632	11	14.34	14.56	4753	1.28E-02
102.0914	70	20.55	20.19	8615	1.28E-02
102.1278	38	18.23	18.63	14900	1.28E-02
102.1278	243	18.31	18.28	17539	1.29E-02
102.5339	105	16.57	16.29	4867	1.30E-02
102.9704	293	14.35	14.42	13212	1.30E-02
103.0165	294	15.75	16.05	7172	1.30E-02
103.0391	126	19.59	19.69	12097	1.31E-02
103.0391	13	15.45	15.67	14991	1.31E-02
103.0543	61	23.83	23.8	7428	1.32E-02
103.0585	91	16.08	16.17	17124	1.32E-02
103.0948	287	15.77	15.96	12863	1.32E-02
103.0948	11	15.77	15.85	14412	1.33E-02
103.5753	25	15.62	16.32	16700	1.34E-02
103.803	24	17.95	17.87	17548	1.34E-02
103.9557	34	19.72	19.66	16935	1.35E-02

103.9557	72	20.08	20.02	16624	1.35E-02
104.0088	297	17.23	17.3	16747	1.35E-02
104.037	258	15.89	15.8	4833	1.36E-02
104.037	10	15.22	15.28	16262	1.36E-02
104.0496	23	16.39	17.88	2226	1.36E-02
104.053	68	20.61	20.73	13404	1.37E-02
104.0577	61	20.53	20.32	2013	1.37E-02
104.061	288	14.44	14.55	13037	1.38E-02
104.061	11	14.17	13.89	4146	1.38E-02
104.0707	10	15.31	15.47	7157	1.39E-02
104.0707	74	21.17	21.51	881	1.39E-02
104.1072	41	24.75	25.14	470	1.40E-02
104.1091	107	18.97	19.93	20	1.40E-02
105.0336	28	20.02	20.96	415	1.41E-02
105.037	68	16.14	16.81	3488	1.41E-02
105.0449	14	17.33	17.55	796	1.42E-02
105.0699	25	20.54	20.42	1423	1.42E-02
105.0741	73	16.51	16.71	17815	1.43E-02
105.1009	108	20.5	20.44	15990	1.43E-02
105.1041	41	27.27	27.29	13850	1.44E-02
105.106	41	20.51	20.42	7146	1.44E-02
105.1101	42	20	20.18	6413	1.45E-02
105.9538	76	18.81	18.82	13813	1.45E-02
105.9539	34	18.46	18.68	649	1.46E-02
106.0369	28	15.4	17.42	1295	1.46E-02
106.0527	238	16.17	15.97	10628	1.46E-02
106.0527	10	15.46	15.5	16350	1.46E-02
106.0652	31	20.34	21.01	12069	1.46E-02
106.0684	92	16.69	16.79	4936	1.47E-02
106.0685	289	12.14	12.51	17190	1.47E-02
106.0734	281	14.97	15.11	17319	1.47E-02
106.0734	11	15.15	15.23	11814	1.48E-02
106.0857	33	17.76	18.04	8699	1.48E-02
106.0862	91	16.22	16.54	1402	1.49E-02
106.1074	41	22.99	23.01	14313	1.49E-02
106.1102	107	20.09	20.27	1108	1.50E-02
106.5892	32	16.98	17.22	10406	1.50E-02
106.9507	94	23.88	23.55	10172	1.50E-02

106.9921	297	18.65	18.9	4600	1.50E-02
107.0492	62	20.78	20.69	3568	1.50E-02
107.0493	294	16.89	17.25	4318	1.50E-02
107.0605	13	15.82	15.82	8493	1.51E-02
107.0605	287	14.94	15.24	13595	1.51E-02
107.0655	68	18.66	18.67	12539	1.51E-02
107.073	30	19.4	19.23	9980	1.51E-02
107.0856	71	19.2	19.2	15420	1.51E-02
107.0856	25	20.04	19.94	2861	1.51E-02
107.1083	107	20.03	20.2	1396	1.51E-02
107.1108	108	19.03	19.31	597	1.51E-02
107.3435	24	20.05	20.03	13671	1.52E-02
107.6744	50	16.09	16.59	10923	1.53E-02
107.7285	100	17.98	17.63	6497	1.53E-02
107.967	9	16.36	16.4	14800	1.54E-02
108.0114	58	18.11	17.92	10235	1.55E-02
108.0445	29	19.03	19.15	12038	1.56E-02
108.0558	281	17.17	17.13	8518	1.56E-02
108.0558	10	16.32	16.36	17850	1.56E-02
108.0684	10	14.1	14.14	11867	1.56E-02
108.0809	28	19.03	18.84	15144	1.56E-02
108.0809	280	17.16	17.53	960	1.57E-02
108.089	281	15.27	15.31	9825	1.59E-02
108.089	12	15.77	15.67	628	1.60E-02
108.1737	50	15.97	15.77	6713	1.60E-02
108.9488	107	19.77	19.97	1350	1.60E-02
109.0286	287	15.03	15.23	11892	1.61E-02
109.0286	10	15.02	15.1	11999	1.62E-02
109.0523	28	17.85	17.8	14202	1.64E-02
109.0634	45	19.04	18.99	14845	1.64E-02
109.0634	68	21.17	21.15	13678	1.65E-02
109.0649	238	21.46	21.9	7774	1.65E-02
109.0649	23	19.62	19.55	16996	1.65E-02
109.0762	284	14.93	15.38	2204	1.66E-02
109.0762	10	15.38	15.47	14734	1.66E-02
109.1012	71	20.56	20.95	3317	1.66E-02
109.1012	24	21.25	21.41	6799	1.66E-02
109.9892	290	15.99	16.22	708	1.66E-02

110.006	28	18.05	17.89	17342	1.67E-02
110.0202	284	15.7	15.57	4142	1.67E-02
110.035	39	17.08	17.12	13454	1.67E-02
110.0601	77	18.67	18.64	17767	1.69E-02
110.0601	29	20.85	20.75	17403	1.69E-02
110.0683	10	15.85	15.78	1305	1.69E-02
110.0686	294	15.91	15.66	981	1.69E-02
110.0714	67	19.5	18.99	4503	1.70E-02
110.0714	298	22.52	21.65	3047	1.70E-02
110.0736	297	15.55	15.12	8010	1.71E-02
110.0966	273	14.89	15.13	10483	1.71E-02
110.0966	10	14.28	14.68	1175	1.71E-02
110.1047	12	17.26	17.3	11502	1.71E-02
111.0204	295	15.09	15.11	10911	1.72E-02
111.0442	76	18.21	18.47	14465	1.72E-02
111.0442	21	18.89	18.88	13830	1.73E-02
111.0554	287	18.09	18.39	7662	1.74E-02
111.0554	10	18.39	18.45	12754	1.75E-02
111.0592	69	16.73	16.4	4668	1.75E-02
111.0635	28	16.91	16.98	17712	1.75E-02
111.0635	283	14.85	15.04	13903	1.76E-02
111.0684	298	16.04	15.36	2332	1.76E-02
111.0748	298	18.36	17.73	2793	1.77E-02
111.0805	24	19.82	20.08	5264	1.77E-02
111.0805	73	19.15	19.48	6219	1.77E-02
111.0918	10	14.95	15.08	9839	1.78E-02
111.0918	276	15.69	15.83	15301	1.78E-02
111.1169	24	19.77	19.9	7561	1.79E-02
111.117	287	15.49	15.61	15061	1.79E-02
111.722	114	16.93	17.22	10360	1.79E-02
111.9685	71	17.68	18.28	703	1.80E-02
111.9685	296	19.32	19.48	10626	1.80E-02
111.9685	13	15.69	15.85	11987	1.80E-02
111.9846	11	16.82	16.96	6245	1.81E-02
112.0181	295	14.67	15.2	3996	1.81E-02
112.0394	90	17.26	17.51	12675	1.81E-02
112.0395	12	16.68	16.74	5785	1.82E-02
112.0395	291	16.65	16.75	15602	1.82E-02

112.0475	11	14.31	14.47	5130	1.82E-02
112.0506	37	19.48	19.72	7150	1.82E-02
112.0507	286	17.47	17.58	14546	1.83E-02
112.0588	285	14.7	15.39	372	1.83E-02
112.0588	9	14.35	14.4	10045	1.84E-02
112.0619	86	17.72	17.69	16167	1.85E-02
112.0619	293	18.19	18.35	17031	1.87E-02
112.0619	13	18.86	18.82	7563	1.87E-02
112.0758	30	18.13	18.37	16310	1.87E-02
112.0839	284	15.65	15.53	3171	1.88E-02
112.084	10	16.08	16.09	11951	1.88E-02
112.087	297	15.9	15.75	14183	1.89E-02
112.0871	8	14.34	14.54	5635	1.89E-02
112.1122	39	17.94	17.18	2166	1.90E-02
112.1122	266	15.83	15.83	14143	1.91E-02
112.8958	47	25.15	25.38	2344	1.91E-02
112.898	47	16.78	17.36	10881	1.91E-02
112.9669	10	14.92	14.93	9387	1.91E-02
112.9669	296	17.67	17.7	15604	1.92E-02
112.9786	291	16.46	16.48	14335	1.93E-02
112.9824	10	14.9	15.08	9068	1.94E-02
112.9851	290	15.17	15.69	3080	1.94E-02
112.9879	289	15.23	15.13	5582	1.94E-02
113.0235	290	15.59	15.78	14642	1.95E-02
113.0235	11	15.59	15.64	15576	1.95E-02
113.0347	81	17.78	17.98	16224	1.96E-02
113.0347	12	18.08	18.14	12957	1.96E-02
113.0598	78	18.73	18.47	2518	1.96E-02
113.0598	22	19.81	19.87	14851	1.96E-02
113.071	131	19.2	19.21	7810	1.97E-02
113.0711	12	16.78	16.81	16656	1.97E-02
113.0792	10	14.41	13.97	1961	1.98E-02
113.0963	12	17.09	17.17	15539	1.98E-02
113.1075	287	16.53	16.67	11151	1.98E-02
113.1075	11	15.11	15.2	9905	1.98E-02
113.1326	22	17.25	17.61	9623	1.98E-02
113.9638	67	22.26	21.98	4420	1.98E-02
113.9638	14	20.41	20.27	6029	1.99E-02

114.03	10	14.62	14.75	8080	1.99E-02
114.03	284	14.82	15.32	2983	2.00E-02
114.0373	27	18.75	19.92	5481	2.00E-02
114.0374	282	14.56	15.15	4234	2.00E-02
114.0632	78	17.66	18.01	1623	2.00E-02
114.0663	43	27.56	27.73	6943	2.00E-02
114.0684	78	19.89	19.62	17460	2.00E-02
114.073	35	18.09	18.39	6080	2.01E-02
114.0914	29	22.69	22.94	11103	2.01E-02
114.1278	38	18.81	19.03	11179	2.01E-02
114.8929	47	23.79	24.04	4291	2.01E-02
114.9609	292	15.19	15.67	3187	2.02E-02
114.9643	293	17.63	17.91	2776	2.02E-02
114.9672	289	17.47	17.75	3522	2.03E-02
114.9716	63	18.3	18.2	8574	2.03E-02
114.9717	10	14.98	15.12	12412	2.04E-02
114.9717	295	19.19	19.32	965	2.04E-02
115.0288	11	14.45	14.65	10449	2.05E-02
115.0288	288	14.49	14.33	9806	2.05E-02
115.0388	19	16.25	16.75	8839	2.05E-02
115.0391	284	14.75	15.08	10138	2.05E-02
115.0503	34	19.94	20.03	11422	2.06E-02
115.0543	31	21.32	21.28	10468	2.06E-02
115.0544	292	16.69	16.76	15345	2.06E-02
115.0582	10	14.05	14	13444	2.07E-02
115.0633	78	20.18	20.41	3739	2.08E-02
115.0695	43	23.07	23.24	7495	2.08E-02
115.0724	78	16.66	17.07	2972	2.09E-02
115.0754	23	19.4	19.87	2973	2.10E-02
115.0867	23	17.2	17.53	12850	2.11E-02
115.0867	124	18.44	18.34	14881	2.11E-02
115.0867	294	16.03	16.39	1329	2.11E-02
115.0948	28	18.67	18.94	11163	2.12E-02
115.0948	278	17.04	17	13287	2.12E-02
115.9642	72	18.08	17.71	3880	2.12E-02
115.9642	297	19.6	19.28	901	2.13E-02
115.9642	12	16	16.03	15561	2.13E-02
115.9794	287	15.59	15.87	11355	2.14E-02

116.002	293	15.83	15.71	2142	2.14E-02
116.0166	9	14.09	14.26	7247	2.15E-02
116.0343	108	16.81	17.02	9417	2.15E-02
116.0528	85	17.97	17.52	2263	2.15E-02
116.0533	24	17.99	17.76	17045	2.15E-02
116.0577	32	17.51	17.15	8359	2.16E-02
116.0675	74	18.7	18.88	6110	2.16E-02
116.0707	72	27.53	27.6	12340	2.16E-02
116.073	73	20.62	20.78	6705	2.17E-02
116.0784	74	17.05	17.07	12613	2.17E-02
116.0802	286	15.26	14.98	8397	2.17E-02
116.0806	9	15.05	15.11	13829	2.18E-02
116.107	79	18.67	18.41	14717	2.18E-02
116.1071	39	19.09	19	15660	2.18E-02
116.8909	47	20.81	21.09	3947	2.19E-02
116.9531	59	18.4	18.51	6457	2.20E-02
116.972	297	19.12	18.75	408	2.20E-02
116.9721	9	16.08	16.09	16580	2.22E-02
116.9761	293	16.15	16.47	4743	2.23E-02
117.0557	283	14.99	15.01	15934	2.23E-02
117.0573	63	17.91	18.12	10798	2.24E-02
117.066	11	14.02	14.34	3767	2.25E-02
117.0677	73	19.28	19.32	16330	2.26E-02
117.0699	26	19.17	19.14	17500	2.29E-02
117.074	73	23.38	23.43	14606	2.29E-02
117.0768	73	18.34	18.38	17422	2.29E-02
117.9596	10	14.81	14.87	15963	2.30E-02
117.9596	297	18.45	18.1	221	2.30E-02
117.9798	295	14.49	14.69	10520	2.31E-02
118.05	10	13.73	13.89	5255	2.31E-02
118.0612	97	17.42	17.66	13909	2.32E-02
118.0652	65	22.89	22.77	3259	2.32E-02
118.0685	12	17.02	17.1	17778	2.32E-02
118.0823	70	19.71	20.49	216	2.33E-02
118.0863	72	28.27	28.82	439	2.34E-02
118.0887	69	21.43	22.01	663	2.34E-02
118.099	70	16.51	17.61	1410	2.34E-02
118.1227	40	21.57	21.41	8747	2.35E-02

118.9675	297	17.67	17.23	160	2.35E-02
118.9675	8	14.81	14.85	17199	2.35E-02
119.0161	69	20.39	20.25	14744	2.35E-02
119.0397	295	14.54	15.09	6019	2.36E-02
119.0491	73	23.21	23.16	8122	2.36E-02
119.0491	45	20.91	20.92	11944	2.37E-02
119.0622	26	20.64	20.78	10793	2.37E-02
119.0685	229	18.67	18.64	10147	2.37E-02
119.0685	63	19.72	19.62	2804	2.38E-02
119.073	61	18.01	18.07	16926	2.38E-02
119.0832	69	19.79	20.36	357	2.38E-02
119.0855	25	22.82	22.81	16182	2.38E-02
119.0896	71	23.97	24.56	391	2.39E-02
119.0923	69	18.64	19.18	531	2.39E-02
119.126	222	18.53	18.5	9955	2.40E-02
119.1261	11	17.64	17.65	15961	2.40E-02
119.9572	295	15.36	15.04	899	2.40E-02
120.0032	95	21.25	20.85	1045	2.40E-02
120.0112	11	14.74	14.75	15603	2.40E-02
120.0113	278	15.69	16.02	1530	2.40E-02
120.0237	298	23.14	23.4	4167	2.40E-02
120.0444	31	19.59	19.95	2860	2.41E-02
120.0445	65	18.34	18.37	9263	2.42E-02
120.0525	73	19.42	19.35	6273	2.42E-02
120.0526	45	17.15	17.1	17652	2.42E-02
120.0655	91	20.79	21.03	3369	2.45E-02
120.0769	61	18.47	18.52	17830	2.45E-02
120.0808	63	27.63	27.51	1420	2.46E-02
120.0832	61	20.98	20.57	275	2.46E-02
120.0904	68	20.11	20.69	345	2.46E-02
120.0929	69	18.32	18.78	2159	2.48E-02
120.965	295	15.01	14.82	3292	2.48E-02
120.9873	108	17.44	17.56	6767	2.50E-02
121.0077	297	16.99	17.26	4219	2.51E-02
121.0147	275	14.65	14.81	17721	2.52E-02
121.0189	294	15.26	15.73	1298	2.52E-02
121.0284	27	19.94	19.53	6378	2.53E-02
121.0397	246	19.18	19.19	12660	2.53E-02

121.0397	11	17.4	17.36	13379	2.54E-02
121.0648	72	19.89	19.91	15074	2.54E-02
121.0689	91	16.16	16.47	2173	2.54E-02
121.0761	12	13.69	14.16	1609	2.54E-02
121.0778	61	19.14	19.05	2403	2.54E-02
121.0841	61	24.2	23.98	338	2.55E-02
121.0867	61	17.83	17.79	8454	2.56E-02
121.1011	69	20.86	20.79	11555	2.57E-02
121.1012	24	20.41	20.45	15813	2.57E-02
121.9662	34	20.57	20.41	1276	2.58E-02
121.9662	79	21.58	21.58	16110	2.59E-02
122.0193	298	20.24	20.25	14840	2.59E-02
122.0274	286	16.5	16.8	2100	2.60E-02
122.06	69	19.68	19.62	13247	2.60E-02
122.0601	29	19.96	19.88	12136	2.61E-02
122.0682	74	16.05	16.07	13178	2.62E-02
122.0682	11	14.17	14.24	16631	2.62E-02
122.0713	291	17.33	17.26	17578	2.63E-02
122.0713	11	17.83	17.75	14132	2.63E-02
122.0812	284	15.22	15.55	10783	2.64E-02
122.0813	11	14.71	14.8	15946	2.65E-02
122.0875	61	18.92	18.91	6672	2.65E-02
122.0964	33	18.95	18.91	7244	2.66E-02
122.0964	87	17.83	17.45	1931	2.66E-02
122.0965	240	19.72	19.8	8160	2.67E-02
122.1045	85	17.26	17.1	14571	2.67E-02
122.1046	10	17.28	17.32	12362	2.67E-02
122.9246	130	24.95	25.29	707	2.68E-02
123.0305	278	15.45	15.22	9368	2.68E-02
123.0404	293	15.88	15.8	2915	2.68E-02
123.044	46	21.54	21.44	3853	2.69E-02
123.044	74	23.87	23.9	16454	2.69E-02
123.0553	36	19.41	19.75	5360	2.69E-02
123.0553	88	18.06	18.41	9939	2.70E-02
123.0635	286	15.07	15.19	10972	2.70E-02
123.0635	10	15.32	15.45	10151	2.70E-02
123.0674	32	16.39	15.56	4640	2.70E-02
123.0702	39	18.93	19.11	15849	2.71E-02

123.0804	69	20.95	21.18	9158	2.72E-02
123.0805	25	22.08	22.19	11844	2.72E-02
123.0917	36	17.62	17.36	9249	2.74E-02
123.0998	11	14.41	14.6	4975	2.75E-02
123.1168	24	20.64	20.69	17572	2.76E-02
123.1168	70	19.99	19.99	15844	2.76E-02
123.9279	131	17.01	17.85	483	2.77E-02
123.9408	8	15.52	15.61	8681	2.77E-02
123.9644	34	19.33	19.21	5003	2.78E-02
123.9644	77	20.36	20.45	5510	2.78E-02
124.0393	27	18.89	19.18	4955	2.79E-02
124.0427	29	17.18	17.53	9721	2.79E-02
124.0474	73	20.05	19.97	7301	2.79E-02
124.0475	11	15.22	15.55	10101	2.80E-02
124.0506	14	15.74	16.03	11294	2.80E-02
124.0506	283	15.13	15.47	16447	2.81E-02
124.0586	34	15.41	15.95	14359	2.82E-02
124.0757	32	20.08	19.91	17102	2.84E-02
124.0839	20	18.16	17.97	9205	2.84E-02
124.0843	243	18.92	19.08	12658	2.84E-02
124.0868	281	17.39	17.33	17737	2.84E-02
124.087	10	16.73	16.76	9278	2.85E-02
124.1121	284	15.41	15.29	13158	2.86E-02
124.1121	11	16.47	15.87	4548	2.88E-02
124.1203	11	16.53	16.6	15775	2.88E-02
124.1203	288	15.91	15.89	9740	2.88E-02
124.2107	171	17.25	16.82	4944	2.91E-02
124.7253	101	17.15	16.51	3240	2.91E-02
124.9227	132	22	22.33	1189	2.91E-02
124.9564	281	15.52	15.15	9300	2.91E-02
125.0026	296	18.39	18.64	4401	2.92E-02
125.0209	123	20.22	20.27	16408	2.93E-02
125.0234	11	14.91	14.95	10033	2.93E-02
125.0234	283	15.5	15.38	11307	2.93E-02
125.0346	281	14.79	15.19	11743	2.93E-02
125.0346	10	14.64	14.67	14593	2.93E-02
125.0427	287	16.14	16.33	9907	2.94E-02
125.0427	10	15.48	15.57	17748	2.94E-02

125.0597	77	19.26	19.08	5190	2.95E-02
125.0597	288	18.59	18.04	5759	2.96E-02
125.0598	18	19.74	19.75	14577	2.96E-02
125.0672	39	17	17.68	2625	2.96E-02
125.071	88	17.6	17.98	3699	2.96E-02
125.071	199	19.27	18.94	16133	2.96E-02
125.071	22	18.15	18.39	13787	2.97E-02
125.0822	284	15.49	15.38	7538	2.97E-02
125.0823	10	15.72	15.82	15792	2.98E-02
125.0961	25	20	19.79	2692	2.99E-02
125.0961	74	19.29	19.05	10309	3.00E-02
125.1074	31	21.06	21.22	9271	3.00E-02
125.1325	21	18.77	18.95	6895	3.00E-02
125.1325	249	16.1	16.18	10903	3.00E-02
125.9642	10	15.14	15.23	12518	3.01E-02
125.9642	290	17.56	17.72	12063	3.01E-02
125.9723	295	16.67	16.34	396	3.02E-02
126.0219	56	22.81	22.56	10785	3.03E-02
126.0464	16	16.11	16.15	10838	3.03E-02
126.055	80	18.26	18.16	9162	3.03E-02
126.055	25	18.82	18.75	12959	3.04E-02
126.0631	284	15.24	15.3	17255	3.04E-02
126.0632	10	15.72	15.76	11012	3.04E-02
126.0662	83	18.02	17.87	11895	3.05E-02
126.0744	269	15.73	16.15	2203	3.05E-02
126.0744	15	14.71	14.65	10787	3.06E-02
126.0775	280	15.77	16.14	14236	3.06E-02
126.0775	11	15.89	15.71	16425	3.07E-02
126.0914	32	23.26	22.94	16504	3.08E-02
126.0995	12	16.36	16.16	17060	3.08E-02
126.0995	285	15.49	15.58	15555	3.09E-02
126.1107	31	17.13	17.57	2383	3.09E-02
126.7885	155	17.33	16.54	2779	3.09E-02
126.9209	134	17.54	17.57	10657	3.10E-02
126.9555	12	14.68	14.72	13681	3.10E-02
126.959	290	15.1	15.35	4616	3.11E-02
126.9676	295	14.8	14.68	16093	3.12E-02
126.9721	292	16.55	16.68	17908	3.13E-02

127.0323	168	18.03	17.96	16024	3.13E-02
127.039	72	19.75	19.75	14692	3.13E-02
127.039	22	19.36	19.15	4518	3.13E-02
127.0447	289	17.52	17.79	2950	3.13E-02
127.0502	29	18.2	18.02	3293	3.13E-02
127.0584	10	14.33	14.26	10145	3.16E-02
127.0584	283	15.31	14.71	864	3.16E-02
127.0615	10	14.56	14.78	6350	3.16E-02
127.0616	284	15.52	15.39	10448	3.16E-02
127.0727	288	18.42	18.45	17914	3.16E-02
127.0727	11	16	16.02	15089	3.16E-02
127.0754	80	18.96	18.59	911	3.16E-02
127.0754	16	19.37	19.38	12851	3.17E-02
127.0754	294	18.35	18.25	16688	3.17E-02
127.0867	288	15.79	15.96	12676	3.17E-02
127.0868	13	16.49	16.05	13260	3.18E-02
127.0947	31	19.46	19.09	17885	3.19E-02
127.1117	24	18.74	18.69	5714	3.19E-02
127.1118	87	18	18.02	14080	3.19E-02
127.123	38	17.86	17.97	17058	3.20E-02
127.275	172	17.09	16.5	4126	3.20E-02
127.954	288	15.65	15.57	14919	3.21E-02
127.9668	290	18.23	18.39	1058	3.21E-02
128.0177	56	18.17	18.24	12625	3.21E-02
128.0343	279	15.38	15.38	6419	3.22E-02
128.0343	10	14.71	14.91	8957	3.22E-02
128.0424	11	14.74	14.96	11530	3.22E-02
128.0424	289	15.1	15.14	12186	3.23E-02
128.0455	86	17.21	17.26	10503	3.24E-02
128.0455	11	16.82	17.15	5097	3.24E-02
128.0456	294	17.24	17.04	3004	3.25E-02
128.0568	12	15.93	15.91	13618	3.25E-02
128.062	25	20.65	20.5	5798	3.25E-02
128.0706	72	19.82	20.1	410	3.26E-02
128.0788	287	15.28	15.18	6146	3.26E-02
128.0788	10	15.2	15.08	8145	3.26E-02
128.0818	39	18.44	17.6	7005	3.26E-02
128.0819	273	15.15	15.81	2140	3.27E-02

128.107	33	19.85	19.53	5807	3.27E-02
128.1071	262	18.71	19	731	3.27E-02
128.1152	11	14.43	14.64	13073	3.28E-02
128.1434	270	16.4	16.42	16936	3.28E-02
128.1435	10	15.46	15.44	12292	3.28E-02
128.6988	163	18.02	17.09	1717	3.28E-02
128.9508	61	21.66	21.6	10608	3.29E-02
128.9508	16	19.02	18.95	14647	3.29E-02
128.9712	291	16.2	16.35	6228	3.29E-02
129.0404	293	16.18	16.26	15116	3.30E-02
129.0547	22	17.76	17.98	13739	3.30E-02
129.0659	111	24.44	24.57	9235	3.30E-02
129.0699	24	18.68	18.39	6319	3.30E-02
129.07	289	15.5	15.42	3203	3.31E-02
129.0741	9	14.4	14.37	6806	3.31E-02
129.0741	283	15.08	15.16	10169	3.32E-02
129.0911	12	17.69	17.77	15156	3.33E-02
129.1023	94	15.96	16.48	1716	3.34E-02
129.1023	11	15.46	15.64	4714	3.34E-02
129.1023	283	17.85	17.91	8617	3.34E-02
129.1105	10	14.32	14.58	17290	3.34E-02
129.1387	297	15.33	15.88	5488	3.34E-02
129.8313	152	17.34	16.26	2147	3.34E-02
129.9513	290	16.85	17.01	792	3.35E-02
129.9542	290	16.64	16.78	1951	3.35E-02
129.9587	7	14.83	15.05	8475	3.36E-02
129.9587	288	17.73	18.01	7234	3.36E-02
129.9791	292	15.64	15.75	17138	3.36E-02
129.9951	290	15.49	15.22	8905	3.36E-02
130.008	77	18.77	19.04	8412	3.38E-02
130.008	15	17.23	17.43	6490	3.38E-02
130.008	298	22.98	23.21	4651	3.38E-02
130.0322	286	15.29	15.02	16303	3.39E-02
130.0322	15	15.57	15.61	6493	3.39E-02
130.0499	30	22.41	22.38	11511	3.40E-02
130.0499	97	25.55	25.66	11131	3.40E-02
130.0595	11	17.6	17.62	12436	3.41E-02
130.0595	82	18.71	18.75	5000	3.41E-02

130.0651	63	19.98	19.97	7279	3.42E-02
130.0692	42	19.22	19.86	4805	3.42E-02
130.0692	108	20.22	20.38	6925	3.42E-02
130.0861	70	25.08	25.06	10830	3.43E-02
130.1227	25	17.99	18.03	17215	3.44E-02
130.159	84	18.06	18.12	16943	3.44E-02
130.159	35	19.77	19.59	2385	3.45E-02
130.3357	167	17.7	17.49	8103	3.45E-02
130.8396	164	17	16.66	6454	3.47E-02
130.9664	64	19.53	19.31	8950	3.47E-02
130.9665	11	16.87	16.89	17727	3.48E-02
130.9918	295	14.26	14.68	10149	3.48E-02
131.0051	295	14.54	14.36	3638	3.49E-02
131.0114	296	17.75	18.03	5338	3.50E-02
131.047	101	16.76	17.06	2555	3.51E-02
131.0492	61	21.8	21.67	1634	3.51E-02
131.053	25	18.15	18.12	13308	3.52E-02
131.0533	99	21.19	21.24	16465	3.52E-02
131.0638	40	19.54	18.82	5643	3.52E-02
131.0702	113	15.78	15.97	6954	3.53E-02
131.0704	12	17.15	17.37	7092	3.53E-02
131.0818	71	18.75	19.5	2651	3.55E-02
131.0833	296	15.12	15.03	16170	3.55E-02
131.0834	10	13.35	11.92	3109	3.56E-02
131.0855	25	19.3	19.32	11914	3.56E-02
131.0896	70	21.12	21	9165	3.57E-02
131.0929	154	19.43	18.45	89	3.57E-02
131.093	249	13.87	12.88	6685	3.57E-02
131.118	278	17.4	17.67	6500	3.58E-02
131.118	89	15.09	15.12	12816	3.59E-02
131.118	10	15.32	15.22	12757	3.60E-02
131.1625	10	14.06	14.41	1030	3.60E-02
131.9298	290	15.98	16.25	5532	3.60E-02
131.967	292	15.13	14.87	11140	3.60E-02
131.9699	292	14.62	15.14	1935	3.61E-02
131.9743	13	16.35	16.51	10651	3.61E-02
131.9743	296	20.16	20.32	5860	3.62E-02
132.0037	298	20.12	20.1	8167	3.63E-02

132.0123	295	14.93	15.28	7271	3.63E-02
132.0444	28	21.12	21	15592	3.63E-02
132.0445	288	15.88	15.72	11191	3.64E-02
132.0525	61	17.76	17.76	7183	3.64E-02
132.0542	100	17.41	17.5	17412	3.64E-02
132.0557	10	17.03	17.06	17605	3.65E-02
132.0656	83	23.61	23.88	4407	3.65E-02
132.0767	45	24.43	24.23	8330	3.65E-02
132.0808	63	20.31	20.4	16628	3.66E-02
132.1019	65	26.18	26.06	17789	3.67E-02
132.1048	64	18.04	17.44	5386	3.67E-02
132.9872	103	18.14	17.91	4205	3.67E-02
132.9873	277	15.24	15.47	14686	3.68E-02
132.9873	9	15.53	15.56	14163	3.68E-02
133.007	295	14.81	15.1	11655	3.69E-02
133.0318	68	23.99	24.15	8776	3.70E-02
133.0478	27	17.46	17.75	9071	3.70E-02
133.0523	21	17.34	17.31	15664	3.70E-02
133.0591	71	18.91	18.71	3726	3.71E-02
133.0592	12	17.94	17.89	3531	3.71E-02
133.0648	24	17.8	17.92	17470	3.71E-02
133.0689	83	19.28	19.52	5461	3.71E-02
133.0739	100	18.52	18.23	7996	3.72E-02
133.0761	267	15.79	15.82	14024	3.72E-02
133.0761	13	15.27	15.42	16835	3.73E-02
133.0802	101	20.84	20.78	11935	3.76E-02
133.086	30	17.03	17.67	9122	3.76E-02
133.086	255	16.18	15.78	1798	3.76E-02
133.0972	294	15.37	15.67	9354	3.76E-02
133.0989	64	16.65	17.03	7652	3.77E-02
133.1012	24	20.34	20.31	8116	3.78E-02
133.1012	74	18.76	18.61	10032	3.78E-02
133.1053	64	22.29	22.09	10919	3.78E-02
133.1082	65	17.88	16.96	1208	3.78E-02
133.8247	158	18.2	17.49	1084	3.79E-02
133.9747	295	16.35	16.02	498	3.79E-02
133.9825	282	15.52	15.43	14515	3.79E-02
133.9825	9	15.36	15.42	10239	3.79E-02

134.0189	91	20.67	20.35	2939	3.80E-02
134.0271	283	16.45	16.8	523	3.80E-02
134.0312	68	16.83	17.16	13919	3.80E-02
134.0351	68	19.48	19.67	8216	3.80E-02
134.06	24	20.98	20.65	6665	3.82E-02
134.0601	77	18.21	18.3	15098	3.82E-02
134.0633	93	15.87	16.11	2539	3.82E-02
134.0698	84	15.1	15.55	6844	3.83E-02
134.0713	286	15.55	16.12	1231	3.83E-02
134.0714	10	16.02	16.07	16336	3.83E-02
134.081	44	17.12	17.44	15653	3.84E-02
134.0812	86	19.01	19.11	17797	3.84E-02
134.0812	289	15.68	15.71	17414	3.84E-02
134.0908	287	16.89	16.63	9029	3.84E-02
134.0908	10	14.83	14.82	14512	3.84E-02
134.1046	288	15.04	15.13	11496	3.84E-02
134.1046	11	15.68	15.56	11927	3.85E-02
134.1061	64	17.61	17.5	17084	3.85E-02
134.1086	65	18.32	17.65	4507	3.85E-02
134.1176	40	20.96	20.72	3245	3.86E-02
134.9869	295	14.72	15.42	5763	3.87E-02
135.0029	8	16.05	16.14	14026	3.87E-02
135.0276	68	19.2	19.39	7850	3.87E-02
135.0441	31	18.72	19.02	17758	3.88E-02
135.0553	25	18.27	18.46	4438	3.88E-02
135.0554	288	16.31	16.25	4051	3.88E-02
135.0634	21	16.3	16.03	6573	3.88E-02
135.0666	281	15.78	16.06	4514	3.89E-02
135.0666	9	15.69	15.71	17498	3.89E-02
135.0667	91	20.05	20.22	4480	3.90E-02
135.0804	24	20.35	20.33	11587	3.90E-02
135.0804	76	19.05	18.69	848	3.91E-02
135.1168	24	21.09	21.12	14225	3.91E-02
135.1168	71	20.37	20.41	13598	3.92E-02
135.1209	216	18.8	18.64	7781	3.92E-02
135.121	12	16.83	16.82	5700	3.93E-02
135.5804	151	17.95	17.37	7767	3.93E-02
135.9701	295	14.93	14.61	1060	3.94E-02

135.9772	97	19.32	19.18	6213	3.95E-02
136.0215	27	20.05	19.72	4963	3.96E-02
136.0216	75	19.32	18.78	380	3.96E-02
136.0393	31	20.61	20.72	7097	3.96E-02
136.0393	284	16.09	16.25	8005	3.96E-02
136.0427	75	16.74	16.3	3231	3.97E-02
136.0482	86	21.46	21.39	16583	3.98E-02
136.0506	287	15.42	15.63	13863	3.99E-02
136.0506	10	16.13	16.03	378	3.99E-02
136.0618	260	20.43	20.47	16428	4.01E-02
136.0618	82	18.45	18.5	13099	4.01E-02
136.0619	10	19.1	19.21	13858	4.01E-02
136.0757	41	22.67	22.63	7004	4.02E-02
136.0757	74	24.6	24.53	7584	4.02E-02
136.0839	11	15.6	15.54	6379	4.02E-02
136.0845	147	18.66	18.1	1822	4.03E-02
136.087	286	15.04	15.35	7834	4.03E-02
136.087	10	15.55	15.82	14141	4.04E-02
136.1121	279	16.27	16.59	10465	4.05E-02
136.1121	10	15.48	15.78	14311	4.05E-02
136.1203	11	16.69	16.87	8524	4.05E-02
136.1203	289	15.89	15.78	10976	4.06E-02
136.5883	141	17.37	16.72	2631	4.06E-02
137.0001	286	15.78	15.61	8324	4.07E-02
137.0249	280	15.14	15.36	17528	4.07E-02
137.025	10	15.02	15.03	17018	4.07E-02
137.0458	39	24.12	24.56	2421	4.08E-02
137.0515	84	17.17	17.1	7558	4.09E-02
137.0597	30	19.76	19.54	7445	4.10E-02
137.0597	73	18.95	18.98	15614	4.10E-02
137.0652	10	14.48	14.59	17558	4.10E-02
137.071	122	21.11	22.04	4111	4.11E-02
137.0727	74	23.74	23.69	16040	4.11E-02
137.079	73	21.11	21.02	4496	4.12E-02
137.079	41	20.27	20.03	4087	4.12E-02
137.0822	10	15.08	15.06	16709	4.14E-02
137.0961	25	19.82	20.11	4324	4.15E-02
137.1325	24	20.17	20.22	14231	4.15E-02

137.1325	68	20.37	20.46	17722	4.15E-02
137.9753	98	15.06	15.44	1055	4.15E-02
137.9875	64	17.75	17.79	7822	4.16E-02
137.9875	290	18.18	18.31	872	4.16E-02
138.0008	28	17.64	17.23	8818	4.16E-02
138.0174	9	14.35	14.79	11792	4.18E-02
138.022	284	15	15.18	4883	4.18E-02
138.0342	297	18.2	18.43	6443	4.18E-02
138.0428	38	17.44	17.96	2051	4.18E-02
138.0486	67	24.22	24.22	11973	4.19E-02
138.0549	74	21.37	21.46	12247	4.19E-02
138.0632	13	15.15	14.87	2171	4.20E-02
138.0662	73	21.8	21.71	7897	4.20E-02
138.0662	162	23.55	22.97	12171	4.20E-02
138.0662	24	21.58	21.39	13515	4.20E-02
138.0759	44	17.83	17.14	3825	4.21E-02
138.0913	31	19.23	19.33	14742	4.21E-02
138.0995	10	16.45	16.47	11534	4.22E-02
138.0995	289	15.83	15.93	11044	4.23E-02
138.1278	10	14.52	14.6	15033	4.23E-02
138.1359	10	16.61	16.6	9556	4.23E-02
138.1359	289	16.18	15.97	3327	4.24E-02
138.9063	50	19.09	19.15	16201	4.24E-02
138.9954	10	16.48	16.5	17710	4.25E-02
139.018	294	18.7	18.65	3968	4.26E-02
139.0254	282	13.02	13.84	4131	4.26E-02
139.0295	296	16.76	17.12	1379	4.26E-02
139.039	283	15.32	15.8	9610	4.26E-02
139.039	12	16.13	15.87	5858	4.26E-02
139.0501	40	19.2	19.28	17833	4.28E-02
139.0503	13	17.44	17.45	17911	4.28E-02
139.054	32	18.62	18.46	16801	4.28E-02
139.0583	28	20.22	19.96	13544	4.28E-02
139.0625	25	18.94	18.69	10723	4.29E-02
139.0696	88	17	16.8	15050	4.29E-02
139.0696	11	17.11	17.17	14191	4.30E-02
139.0754	70	20.35	20.18	8285	4.30E-02
139.0866	29	17.13	17.67	1894	4.32E-02

139.0866	288	17.04	16.97	9358	4.32E-02
139.1117	25	20.12	20.12	15734	4.33E-02
139.1118	70	22.01	22.06	15325	4.34E-02
139.5739	155	18.18	17.62	4220	4.34E-02
139.9147	216	16.78	16.59	4062	4.34E-02
139.9148	8	14.01	14.16	12102	4.35E-02
139.9633	289	18.05	18.26	3811	4.35E-02
139.9823	293	15.8	15.55	4508	4.36E-02
139.9879	68	20.46	20.19	238	4.36E-02
139.9879	8	20.29	20.18	10636	4.37E-02
139.988	297	22.78	22.47	751	4.37E-02
139.9987	288	15.25	15.28	17810	4.37E-02
140.03	295	14.52	14.35	1428	4.38E-02
140.0343	10	15.89	15.87	7766	4.38E-02
140.0343	290	16.34	16.4	15650	4.40E-02
140.0443	68	19.45	19.39	17097	4.41E-02
140.0455	283	15.61	15.81	10026	4.42E-02
140.0456	10	15.1	15.07	7269	4.43E-02
140.0495	35	18.3	18.34	12138	4.43E-02
140.0706	74	18.04	18.08	11141	4.43E-02
140.0706	31	19.48	20.05	1206	4.43E-02
140.0729	29	19.21	19.3	12346	4.43E-02
140.078	147	16.64	15.93	8542	4.44E-02
140.0788	13	16.9	16.65	574	4.44E-02
140.0788	289	16.61	16.16	568	4.44E-02
140.0818	37	17.19	16.54	11597	4.44E-02
140.0819	287	17.06	17.21	13943	4.45E-02
140.107	26	18.97	18.65	6846	4.46E-02
140.1151	86	17.36	17.64	9105	4.47E-02
140.1151	13	17.49	17.56	15293	4.47E-02
140.5817	146	16.54	15.96	7634	4.47E-02
140.9034	50	18.41	18.47	15267	4.48E-02
140.9513	293	17.44	17.71	7995	4.50E-02
140.9513	10	15.18	15.28	13511	4.50E-02
140.9618	292	17.24	17.23	5830	4.50E-02
140.9618	9	14.56	14.74	6931	4.51E-02
140.9858	291	16.17	16.06	2710	4.51E-02
140.9858	10	14.76	14.87	3435	4.51E-02

140.9913	8	15.17	15.22	15453	4.52E-02
140.9913	297	17.8	17.44	384	4.52E-02
140.9949	127	21.07	21.23	7318	4.52E-02
140.9958	9	15.88	15.9	17306	4.53E-02
140.9958	297	18.88	18.59	219	4.53E-02
141.0546	83	17.28	17.01	12643	4.53E-02
141.0546	19	17.69	17.56	6296	4.53E-02
141.0659	106	19.86	20.45	1259	4.54E-02
141.0659	11	16.8	17.63	4924	4.54E-02
141.0699	25	19.6	19.27	1507	4.55E-02
141.0699	285	15.64	15.81	16432	4.56E-02
141.0715	76	17.44	18.18	7412	4.56E-02
141.074	10	14.45	14.13	8118	4.56E-02
141.091	76	19.38	19.07	10801	4.56E-02
141.091	22	20.23	20.12	13526	4.56E-02
141.1023	285	15.49	15.76	10159	4.56E-02
141.1023	10	14.92	14.93	12700	4.56E-02
141.1274	87	17.46	16.92	862	4.57E-02
141.1275	13	17.13	17.21	12035	4.57E-02
141.1386	37	17.95	18.31	14230	4.57E-02
141.7227	101	17.67	17.12	1960	4.57E-02
141.9586	70	18.88	18.78	6942	4.58E-02
141.9586	8	19.18	19.34	6761	4.58E-02
141.9833	69	18.94	18.79	9816	4.58E-02
141.9834	296	21.52	21.18	393	4.59E-02
141.9834	7	18.72	18.71	17358	4.59E-02
141.9983	135	16.7	16.77	9316	4.59E-02
142.0247	52	18.09	17.9	12805	4.60E-02
142.0499	292	16.8	16.85	13750	4.60E-02
142.0499	11	16.64	16.65	6860	4.60E-02
142.0611	37	18.69	18.78	4654	4.61E-02
142.0611	238	18.54	18.53	15277	4.61E-02
142.0651	63	18.54	18.2	10120	4.62E-02
142.0692	107	15.52	16.77	2019	4.62E-02
142.0777	22	17.26	17.5	13190	4.62E-02
142.0862	49	21.83	22.04	13393	4.62E-02
142.0863	263	18.36	18.52	8618	4.62E-02
142.0944	283	15.96	16.01	14227	4.63E-02

142.0944	10	16.1	16.2	6523	4.63E-02
142.0975	281	15.62	15.59	6397	4.64E-02
142.1226	28	19.48	18.63	2455	4.66E-02
142.1227	225	19.84	19.55	6109	4.66E-02
142.9386	11	15	15.06	9242	4.66E-02
142.9386	278	17.47	17.05	9459	4.67E-02
142.9481	10	16.44	16.59	2081	4.67E-02
142.9481	281	16.91	16.74	5972	4.67E-02
142.9591	289	16.61	16.82	5314	4.67E-02
142.962	289	17.07	17.26	5446	4.68E-02
142.9669	290	17.32	17.75	2912	4.69E-02
142.967	10	15.13	15.22	17508	4.69E-02
142.9837	295	16.95	16.68	444	4.69E-02
142.9868	295	16.82	16.47	193	4.69E-02
142.9912	8	14.61	14.55	9430	4.69E-02
142.9912	295	17.38	17.04	214	4.70E-02
143.0339	85	17.57	17.48	7657	4.70E-02
143.034	12	16.79	16.76	14634	4.71E-02
143.0342	295	18.06	17.94	9236	4.71E-02
143.0396	268	24.07	24.13	16156	4.72E-02
143.0429	296	15.74	16.11	8892	4.72E-02
143.0646	9	14.2	14.03	6458	4.72E-02
143.0703	19	17.99	18.06	14344	4.72E-02
143.0815	30	23.36	22.75	16322	4.73E-02
143.0851	281	15.66	15.47	10444	4.73E-02
143.0853	25	18.92	18.53	8695	4.73E-02
143.0896	56	17.86	18	13710	4.73E-02
143.1066	25	19.52	19.41	17216	4.74E-02
143.1179	33	20.02	19.98	15010	4.75E-02
143.1179	279	16.35	16.42	13971	4.76E-02
143.1179	104	19.34	19.62	86	4.77E-02
143.126	9	14.48	14.57	12559	4.77E-02
143.959	295	17.66	17.39	526	4.78E-02
143.9809	297	18.29	17.91	330	4.78E-02
143.9969	293	18.42	18.26	553	4.78E-02
144.0236	298	20.38	20.57	5017	4.78E-02
144.0349	296	17.1	17.38	3855	4.80E-02
144.0429	298	19.44	19.67	5055	4.80E-02

144.0655	229	21.27	21.3	16632	4.80E-02
144.0655	62	22.08	22.09	13350	4.81E-02
144.0807	62	20.93	20.84	9953	4.81E-02
144.0848	26	19.24	19.04	12932	4.81E-02
144.1019	73	27.19	26.82	8736	4.82E-02
144.1096	73	15.45	14.92	9363	4.82E-02
144.1383	284	16.69	16.76	17483	4.83E-02
144.1383	11	16.69	16.75	11631	4.83E-02
144.1747	250	15.82	15.68	9826	4.83E-02
144.1747	9	14.86	14.94	17350	4.83E-02
144.966	290	15.45	15.56	10307	4.83E-02
144.982	78	23.95	23.93	15314	4.83E-02
144.9821	33	23.06	22.9	1505	4.84E-02
144.9886	295	14.54	14.47	17686	4.84E-02
145.027	295	15.26	15.6	4235	4.84E-02
145.0351	80	18.05	17.87	1546	4.85E-02
145.0353	298	21.92	21.91	11270	4.87E-02
145.0438	295	15.48	15.8	5647	4.88E-02
145.0495	95	18.98	19.4	3093	4.89E-02
145.0495	11	16.82	16.78	13709	4.89E-02
145.0552	62	19.79	19.79	3992	4.89E-02
145.0552	235	18.44	18.56	1065	4.89E-02
145.0648	284	15.23	15.35	6958	4.92E-02
145.0648	19	17.13	17.89	11624	4.92E-02
145.0679	83	17.45	17.97	3208	4.92E-02
145.068	11	17.75	17.84	13674	4.93E-02
145.0848	38	19.18	18.82	5241	4.93E-02
145.0859	265	16.08	15.87	7472	4.93E-02
145.0972	283	17	17.17	9052	4.94E-02
145.1011	24	20.17	20.11	12891	4.94E-02
145.1052	72	23.34	23.08	10891	4.95E-02
145.1078	72	17.25	16.7	8275	4.95E-02
145.9544	294	16.23	15.99	1878	4.96E-02
145.9805	294	16.46	16.08	209	4.97E-02
145.9855	34	18.66	18.77	14232	4.97E-02
145.9855	76	19.22	19.34	8488	4.98E-02
146.0193	297	18.73	18.77	17269	4.99E-02
146.027	65	20.22	20.35	3409	4.99E-02
146.0305	296	13.56	13.81	9908	4.99E-02

146.0386	296	17.21	17.3	12262	4.99E-02

Raw p values < 0.05
ESI=electrospray ionization
HILIC= hydrophilic interaction liquid chromatography
TPP = thiamine pyrophosphate

Supplemental Table 5. C18/ESI- and HILIC/ESI+ lowest versus highest TPP concentration: tertile analysis

Metabolic Pathway	*m/z*	Time (sec)	Adduct	Putative Annotation	Metabolite p-value	Association with TPP levels	ID score
Aspartate & Asparagine	231.0990	18	M-H[-]	Succinyl-ornithine	0.016	negative	3
Aspartate & Asparagine; Pyrimidine	131.0460	21	M-H[-]	Asparagine	0.035	negative	3
Lysine; Biotin	145.0981	15	M-H[-]	Lysine	0.030	negative	2
Lysine	128.0706	72	M+H[+]	Piperidine-carboxylate	0.019	negative	3
Methionine & Cysteine	190.0543	19	M-H[-]	Acetylmethionine	0.030	negative	1
Branched Chain Amino Acid	157.0982	16	M+ACN-H[-]	Valine	0.046	negative	2
Amino acid pathways; Pyrimidine; Microbiome	143.0825	16	M+ACN-H[-]	Aminobutyrate	0.017	positive	2
Urea Cycle/Amino group; Microbiome	178.0509	20	M-H[-]	Hippurate	0.020	positive	1
Pyrimidine	157.0248	15	M-H[-]	Dihydroorotate	0.029	positive	1
Pentose phosphate	149.0455	17	M-H[-]	Xylose/ Ribose	0.047	negative	1
Linoleate	295.2264	72	M+H[+]	OxoODE	0.028	negative	3
	297.2424	23	M+H[+]	EpOME/ HODE	0.045	negative	3
	329.2324	222	M+H2O+H[+]	HPOT	0.013	positive	3
	546.3318	30	M+K[+]	Lyso-PC	0.029	positive	2
Squalene/ Cholesterol	367.3359	23	M-H20+H[+]	Dehydro-cholesterol	0.007	positive	2
	383.3308	25	M+H[+]	Dehydro-desmosterol	0.002	positive	3

Raw p values < 0.05
ESI=electrospray ionization
HILIC= hydrophilic interaction liquid chromatography
EpOME: Epoxyoctadecenoic acid
HPOT: Hydroperoxy-octadecatrienoic acid
HODE: Hydroxyoctadecadienoic acid
ID= identification
TPP= thiamine pyrophosphate
Lyso-PC: Lysophosphatidylcholine
OxoODE: Oxooctadecadienoic acid
